# siAbasic: a comprehensive database for potent siRNA-6Ø sequences without off-target effects

**DOI:** 10.1093/database/bay109

**Published:** 2018-10-12

**Authors:** Jongyeun Park, Seung Hyun Ahn, Kwang Moon Cho, Dowoon Gu, Eun-Sook Jang, Sung Wook Chi

**Affiliations:** 1 Department of Life Sciences, Korea University, Seoul, Korea; 2 EncodeGEN Co. Ltd., Seoul, Korea

## Abstract

Small interfering RNA (siRNA) is widely used to specifically silence target gene expression, but its microRNA (miRNA)-like function inevitably suppresses hundreds of off-targets. Recently, complete elimination of the off-target repression has been achieved by introducing an abasic nucleotide to the pivot (position 6; siRNA-6Ø), of which impaired base pairing destabilizes transitional nucleation (positions 2–6). However, siRNA-6Ø varied in its conservation of on-target activity (∼80–100%), demanding bioinformatics to discover the principles underlying its on-target efficiency. Analyses of miRNA–target interactions (Ago HITS-CLIP) showed that the stability of transitional nucleation correlated with the target affinity of RNA interference. Furthermore, interrogated analyses of siRNA screening efficiency, experimental data and broadly conserved miRNA sequences showed that the free energy of transitional nucleation (positions 2–5) in siRNA-6Ø required the range of stability for effective on-target activity (−6 ≤ ΔG[2:5] ≤ −3.5 kcal mol^−1^). Taking into consideration of these features together with locations, guanine-cytosine content (GC content), nucleotide stretches, single nucleotide polymorphisms and repetitive elements, we implemented a database named ‘siAbasic’ that provided the list of potent siRNA-6Ø sequences for most of human and mouse genes (≥ ∼95%), wherein we experimentally validated some of their therapeutic potency. siAbasic will aid to ensure potency of siRNA-6Ø sequences without concerning off-target effects for experimental and clinical purposes.

## Introduction

Small interfering RNA (siRNA) has been developed to specifically repress expression of an intended target gene via RNA interference (RNAi) (1). siRNA generally forms a duplex of ∼21 nucleotides (nt), among which one strand, referred to as the ‘guide strand’ or ‘antisense strand’, is designed to be perfectly complementary to the target mRNA sequence (2). When introduced into the cell, the guide strand majorly loads onto Argonaute (Ago), a core effector protein in the RNA-induced silencing complex (RISC), and hybridizes and cleaves the target mRNA, resulting in post-transcriptional gene silencing (3). Because customizing its target specificity requires only a simple procedure, siRNA has been widely used to study genes for loss of function and to apply therapeutics for the knockdown of disease-causing genes. However, not all perfect complementary sequences are equally efficient, requiring the consideration of the critical features that determine the activity of siRNA. Bioinformatics approaches have helped in designing effective siRNA sequences (4). By avoiding the targeting of introns, untranslated regions (UTRs) and the vicinity (∼100 bp) of the start and end codons of open reading frames (ORFs) (5), potent siRNA sequences have been designed and further improved by taking into consideration of the appropriate GC contents and other sequence features (5–7) such as lack of inverted repeats (6) and at least five A/U residues in the 5′ terminal one-third of the guide strand (7).

Endogenously, RNA silencing is triggered by microRNAs (miRNAs). By sharing the same machinery of RISC, siRNAs loaded onto Ago perform the same function as miRNAs, resulting in unintended miRNA-like off-target repression (2). The off-target effect is potentially detrimental, often leading to unwanted phenotypes, such as cell death or the inhibition of cell growth (8). To avoid these deleterious side effects, several approaches have been applied to siRNAs, including bioinformatics based on sequence analysis (9, 10) and chemical modifications (11) such as 2′OMe (12), but their impacts on preventing miRNA-like activity have been limited (13).

**Figure 1:**
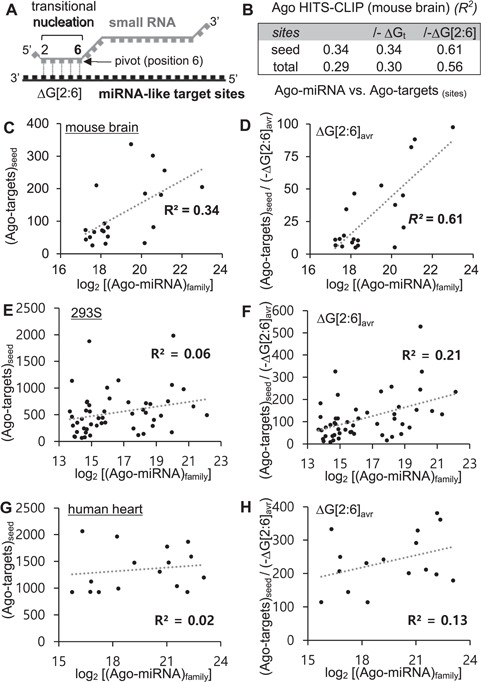
The stability of transitional nucleation correlated with the target binding affinity of RNAi. (A) The transitional nucleation (ΔG[2:6]) is prerequisite to initiate functional miRNA-like target interaction, in which a small RNA loaded into Ago was geometrically tilted (based on Ago–miRNA structure studies), represented as a model (see Supplementary Figure S1). Of note, base pairing in position 6, named pivot, critically functions as transition to triggering RNAi. (B) Considering free energy of transitional nucleation, −ΔG[2:6] was used to denominate number of target sites (seed or total) in Ago HITS-CLIP data from mouse brain, compared with the expression level of cognate miRNAs (log_2_[Ago–miRNA]_family_) by calculating the correlation coefficient (*R*^2^), of which values were indicated in the table. (C–D) *R*^2^ was further improved by considering free energy of transitional nucleation (ΔG[2:6]_avr_). (E–H) The same analyses used in panels C–D except applied to Ago HITS-CLIP data from 293S cells (E–F) or human hearts (G–H).

**Figure 2:**
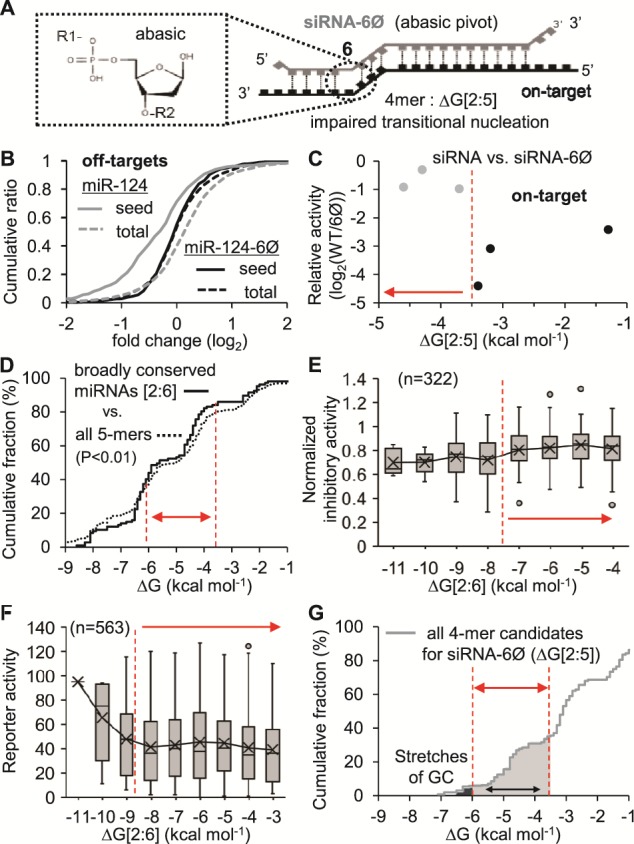
The range of transitional nucleation required to induce potent on-target activity of siRNA. (A) The functional interaction of siRNA-6Ø with on-target mRNA. Notably, siRNA-6Ø has impaired transitional nucleation (ΔG[2:5]) by harboring abasic pivot but possibly compensated by near-perfect matches in the rest of regions. (B) The cumulative fraction of fold changes (log_2_) showed that miRNA-dependent transcripts with high-confidence seed target sites (seed) were significantly downregulated by miR-124 (*P* < 0.01) but completely derepressed by miR-124-6Ø (*P* = 0.54, seed vs. total, KS test). (C) On-target activity of siRNA-6Ø relative to the unmodified siRNA (WT), experimentally derived from six siRNAs (log_2_(IC_50_[WT]/IC_50_[6Ø]). Conserved (gray dots) and non-conserved siRNAs (black dots) are indicated. (D) Free energies from transitional nucleation (ΔG[2:6]) of broadly conserved miRNAs (*n* = 107) are compared with those of all 5-mers by cumulative fraction analysis, showing significant differences (*P* < 0.01, KS test). Distribution of results comprising the cumulative fraction is represented in Supplementary Figure S4A–B. (E) High-confidence siRNAs and their reported normalized inhibitory activities from siRNA experiments (*n* = 322, Supplementary Table S4) within the displayed range of free energy in this figure were plotted against the free energy of transitional nucleation. (F) The same analysis conducted in (E) except for compiled siRNA sequences (*n* = 563) from 15 different siRNA studies and y-axis with reporter activity, which is negatively correlated with normalized inhibitory activity in general. (G) All 4-mer candidates for the impaired transitional nucleation region (position 2–5) of siRNA-6Ø were plotted as analyzed in (D). The appropriate free energy range (solid light gray area) and the cutoff value (−6 ≤ ΔG[2:5] ≤ −3.5 kcal mol^−1^) are indicated. Determined range of transitional nucleation was indicated with red dotted line and solid double arrow. Red dotted line and solid arrow denote the determined cutoff. Distribution of results comprising the cumulative fraction is represented in Supplementary Figure S4C.

It has been proposed that miRNA-like target recognition is initiated by transitional nucleation, i.e. base pairing in positions 2–6 (Figure 1A and Supplementary Figure S1A ); this was experimentally elucidated by the transcriptome-wide identification of miRNA target sites *in vivo* (Ago HITS-CLIP) (14) and a following bioinformatics analysis of noncanonical target sites, referred to as ‘nucleation bulge sites’ (15, 16). In this model, position 6, named the ‘pivot’, has been implicated in critical base pairing that plays a decisive role in the functional miRNA-like target interactions. Based on this concept, the transitional nucleation of siRNA was intentionally destabilized by impairing a base pair in pivot with abasic nucleotide substitution (siRNA-6Ø) (17), thereby achieving complete elimination of the miRNA-like off-target repression (Figure 2A and Supplementary Figure S1B) (18). However, siRNA-6Ø showed variability in its conservation of on-target activity that depends on its sequence composition (∼80–100%). Thus, to minimize the sacrifice of on-target activity, bioinformatics prediction and a database for the results of potent siRNA-6Ø sequences were needed.

By analyzing miRNA–target interactions (Ago HITS-CLIP data), siRNA screening data and miRNA sequences, here, we discovered that the free energy of impaired transitional nucleation (positions 2–5) in siRNA-6Ø required a specific range of stability for effective on-target activity (−6 ≤ ΔG[2:5] ≤ −3.5 kcal mol^−1^). This enabled the design of siRNA-6Ø, of which impaired pivot can potentially be overcome by selected sequences in the transitional nucleation region. To provide opportunities to use this design scheme, we generated comprehensive lists of putative siRNA-6Ø sequences that can target human and mouse genes (covering ≥95% of protein-coding genes, annotated by RefSeq and official gene symbols), and we incorporated them into a database called ‘siAbasic’. Such functionality for siRNA-6Ø sequences would be of use to researchers who need to silence the expression of any gene of experimental or clinical interest with guaranteed on-target activity but without any miRNA-like off-target repression.

## Materials and methods

### Dataset

Clustered Ago-bound mRNA regions (Ago–targets) and the frequency of miRNA sequences associated with Ago (Ago–miRNAs) were retrieved from Ago HITS-CLIP data derived from mouse brain tissues (http://ago.rockefeller.edu/rawdata.php or http://clip.korea.ac.kr/Ago_Clip_data/) (14). Expression level of Ago-loaded top 20 miRNAs in mouse brain was estimated by calculating log_2_ value of normalized sequencing frequency from the sequencing data as previously described (14). To calculate the frequency of miRNA families in Ago HITS-CLIP sequencing data, accumulate sequencing frequency of all members in a given miRNA family was used ( Supplementary Table S1A).

Ago HITS-CLIP reads performed in 293S cell lines were retrieved from the previous publication (19), selecting a subset performed with no arsenite treatment. Abundance of top 50 miRNA families, associated with Ago, was derived from reported expression (calculated from base mean values, Supplementary Table S2A) (19). Ago HITS-CLIP peak regions, identified in 293S (19), and their target abundance, estimated by peak height, were obtained from starBase v2.0 (20) and used to search corresponding seed sites (6-mers, position 2–7).

Ago HITS-CLIP data from human heart tissues (21) were downloaded from GEO database (GSE83410) and mapped using Bowtie2 (http://bowtie-bio.sourceforge.net/bowtie2/) with the local alignment option, allowing one mismatch in each seed (-local -N1) and removing polymerase chain reaction (PCR) duplicates in each experiment. All mapped reads were compiled and selected only within the reproducible clusters in every replicate (*n* = 6). Peak signals of Ago HITS-CLIP were analyzed and normalized by *in silico* random CLIP method (*P* < 0.01) as previously described (14). Abundance of miRNA families was obtained from supplementary data of previous publication (21). Although Ago peak signals from human heart were normalized, seed sites were relatively sparse compared to other Ago HITS-CLIP data. Thus, for the correlation analysis, we further selected 16 miRNA families, of which both Ago-associated abundance and corresponding seed sites were enriched within top 50 (Supplementary Table S2B).

miRNA sequences were obtained from miRBase (http://www.mirbase.org/). High-confidence miRNA target sites, including seed (seed) and/or nucleation bulge sites (nuc), were obtained from miRTCat (http://clip.korea.ac.kr/mirtcat) by setting the PhastCons score cutoffs as 0.9 (16).

### Calculation of thermodynamic stability

To estimate the stability of transitional nucleation, free energies for base pairing via specified regions of small RNAs were calculated with RNAduplex (version 2.1.9) using default parameters (22). The free energy values were calculated by adding an unpaired nucleotide (additional ‘N’ sequence) at both the 5′ and the 3′ ends of specified short sequences (4 or 5 nt) to minimize the miscalculation caused by artificial 5′ or 3′ dangling nucleotides. Intact siRNA or miRNA sequences were used when calculating total free energy (ΔG_t_, ΔG[2:19] between small RNA and target). The free energy values for siRNA-6Øs were calculated after substituting the sixth position into the unpaired nucleotide (‘N’ sequence). For calculating ΔG_t_ of miRNA family, average values of ΔG_t_ were calculated depending on sequencing frequencies of members (Ago–miRNA) as follows (Supplementary Table S1 and S2).

$$\varDelta Gt=\frac{\sum_{i=1}^n\left\{ \left( Ago\_ miRNA\right)i\times \left(\varDelta Gt\right)i\right\}}{\sum_{i=1}^n\ \left( Ago\_ miRNA\right)i}$$

ΔG[2:6]_avr_ was also calculated by the same method if the values from member are different.

### Linear correlation analysis and conservation of on-target activity

Linear regression was used to analyze correlations between the numbers of target sites in the Ago-bound target regions [(Ago–target)_sites_] and the expression levels of the corresponding miRNA families [(Ago–miRNA)_family_] (Supplementary Table S1 and S2), with or without considering free energies ( Supplementary Figure S2). To examine the effect of free energy on correlation coefficient (*R^2^*) value, the number of target sites was normalized by using negative free energy values as denominator in these analyses (Figure 1C–H and Supplementary Fig S2). Of note, Ago HIT-CLIP data contain sequences of all Ago-associated RNAs. In Ago–miRNA–target ternary complex, Ago–miRNA interactions were direct, but mRNA target sites were rather indirectly associated with Ago through base pairing with miRNA. Thus, sequencing frequency of seed target sites tended to be proportionally correlated with frequency of target mRNAs in Ago complex depending on target affinity of each miRNA. Any factor affecting target affinity of miRNA could be applied to normalize frequency of target sites by being used as denominator, expecting to increase the correlation coefficient (*R^2^*) between the abundance of miRNA and the number of target sites.

The efficiency of on-target repression, previously measured as half-maximal inhibitory concentration (IC_50_) ( Supplementary Table S3), was analyzed (Figure 2C and Supplementary Figure S3). For estimating the conservation of on-target activity in siRNA-6Ø, log_2_ ratio of relative activity [IC_50_ value from unmodified siRNA (WT) was divided by siRNA-6Ø (6Ø)] was calculated (Figure 2C and Supplementary Figure S3C).

### Retrieval of siRNA screening data

siRNA sequences and their estimated inhibitory activities from siRNA screening experiments were retrieved from the previous studies. In order to reduce compounding effects from selective Ago loading, siRNA sequences and their estimated inhibitory activities from siRNA screening experiments (23) were further selected by limiting only siRNAs with U in position 1 and lower ΔG[2:6] from antisense strand than that from sense strand (ΔΔG < 0), used as a high-confidence siRNA set (*n* = 322) based on the finding of previous study (24) ( Supplementary Table S4). Additionally, nine high-confidence siRNAs ( Supplementary Table S5), which were selected by the same strategy (*n* = 25; A or U in position 1 and ΔΔG < 0) from the previous publication (25), were analyzed together ( Supplementary Figure S5A) after the activity value was converted to normalized inhibitory activity as previously described (23). The normalized inhibitory activity was examined together with the stability of transitional nucleation (ΔG[2:6], calculated by RNAduplex program). Independently, siRNA results (*n* = 563), which were previously compiled from 13 different publications (26) ( Supplementary Table S6B) and within the displayed range of free energy in Figure 2F, were also retrieved. By selecting ones with U or A in position 1 (*n* = 332) and further narrowing ones with ΔΔG < 0 (*n* = 176) ( Supplementary Table S6B), high-confidence compiled siRNAs ( Supplementary Table S6A) were yielded.

### Distribution of nucleation stability in miRNAs

To measure nucleation stability of miRNAs, human and mouse miRNA sequences were derived from miRBase (http://www.mirbase.org/) and their free energy values of transitional nucleation (ΔG[2:6]) were calculated. Especially, broadly conserved miRNAs (*n* = 107) were retrieved from TargetScan (http://www.targetscan.org). Distribution of the miRNAs depending on ΔG[2:6] was examined and used for cumulative fraction analysis depending on free energy values. To estimate the range of free energy values for transitional nucleation, which may be biologically selected to gain stability, the cumulative distribution was compared with all combination of 5-mers using Kolmogorov–Smirnov (KS) test as previously described (14). The distribution from miRNAs was further compared with the distribution from all combination of 4-mers (4^4^ = 256), which can be served as transitional nucleation in siRNA-6Ø.

### Searching potent target sites for siRNA-6Ø

Following RefSeq annotation, we downloaded only coding sequences (CDSs) in human and mouse from UCSC genome databases (hg19, mm9) (http://genome.ucsc.edu/), where single nucleotide polymorphisms (SNPs) (dbSNP144, dbSNP128) and repeats (RepeatMasker) were masked based on the genome coordination. Only a unique target site of siRNA-6Ø was allowed. In the case where mRNA from RefSeq has multiple genomic coordination from UCSC genome browser, we only followed the annotation represented in NCBI (http://www.ncbi.nlm.nih.gov/). However, when the length of their coding sequence is the same, we merged all marked region of SNPs and repeats for this analysis. Finally, 94 nucleation sequences (4-mers) for siRNA-6Ø were selected because their free energy values are in the discovered range (−6 ≤ ΔG[2:5] ≤ −3.5 kcal mol^−1^) (Figure 2G, Supplementary Figure S6 and Supplementary Table S7), initially used to find the matches in coding sequences excluding the masked region (SNP or repeats). Furthermore, target sites must fulfill the following criteria generally used for siRNA sequences: U in position 1 (for efficient Ago loading), avoiding the region within 100 bp of the first or the last nucleotide of ORFs and nucleotide stretches including GC stretches (>4 nt), GC contents between 32 and 52% (6–10 G or C in total length of 19 nt siRNA-6Øs) and G or C in position 19 (prevent passenger strand from loading onto Ago). When predicted siRNA-6Ø has target sites in mRNAs (RefSeq) other than the intended target gene (official gene symbol), we removed it from our final list in siAbasic. The method was implemented and the results were displayed on the web interface of siAbasic database (http://clip.korea.ac.kr/siabasic/).

### siAbasic infrastructure

siAbasic was implemented mainly by using Python and PHP, set-up in a 12-core server. Especially, web interface was implemented using HTML, CSS and Javascript components. The Javascript component utilized the JQuery library (http://jquery.com/) and the JSON data interchange format (http://www.json.org/). The user is assisted in formulating a query by automatic completion of gene names (official gene symbols), of which requests are processed on the server side by custom Python scripts.

### siRNA synthesis and modification

Custom synthesis and modification services of ST Pharm (Korea) and Bioneer (Korea) were used to chemically synthesize siRNAs. dSpacer (abasic deoxynucleotide) was introduced to position 6 for producing siRNA-6Ø. siRNA duplexes were produced *in vitro* by following the reaction (90°C for 2 min, 30°C for 1 h and 4°C for 5 min). The miRNAs (mmu-miR-124-3p) that we used here were synthesized and duplexed with the same sequences in miRBase (http://www.mirbase.org/). A non-targeting siRNA (NT), derived from cel-miR-67 (*Caenorhabditis elegans*–specific miRNA provided as a negative control by Dharmacon), contains 6Ø in both guide and passenger strands and used as control.

### Cell culture and transfection

The human cervical adenocarcinoma cell line HeLa (ATCC CCL-2) and mouse neuroblastoma cell line Neuro-2a (N2a, ATCC CCL-131) were maintained in Dulbecco’s modified Eagle’s medium (Gibco) supplemented with 10% fetal bovine serum (Gibco), 100 U/ml^−1^ penicillin and 100 μg/ml^−1^ streptomycin at 37°C with 5% CO^2^ incubation. The cells were transfected by using Lipofectamine 2000 (Invitrogen) or RNAiMAX (Invitrogen) with 50 nM RNA duplexes according to the general protocol provided by the manufacturer, unless otherwise indicated. The cells were generally collected 24 h after transfection in all experiments.

### RNA-Seq experiments and cumulative fraction analysis

RNA-Seq experiments were performed and further analyzed as cumulative fractions, as previously described (18). In brief, RNA-Seq experiments were performed in neuroblastoma cell line, N2a, transfected with miR-124 or miR-124-6Ø. A non-targeting control siRNA (NT-6Ø) that was derived from cel-miR-67 (*C. elegans*–specific miRNA) was further modified by introducing abasic pivot substitution (6Ø) in both guide and passenger strands (guide strand: 5′p-UACUCØUUCUAGGAGGUUGUGAdTdT-3′, passenger strand: 5′ p-UCACAØ CCUCCUAGAAAGAGUAdTdT-3′) and used as control. Total RNAs were purified by RNeasy Mini Kit (Qiagen) according to the protocol provided by the manufacturer. By using the poly-adenylation cDNA method, RNA-Seq libraries were constructed, sequenced and de-multiplexed by Omega Bio-tek (Norcross, GA, USA) using a HiSeq 2500 platform for 100 nt pair-end reads. The demultiplexed sequencing reads were aligned to the mouse genome (mm9) using TopHat2 (pair-end reads, tophat2 -a 4 -g 1 --b2-sensitive -r 100 --mate-std-dev = 50 --no-discordant) under a supply of RefSeq gene annotations. Transcript levels were quantified using Cufflinks (cufflinks -N -b) and differential transcript profiles were analyzed by Cuffdiff (Cuffdiff --FDR = 0.1 -b -N --min-alignment-count = 10 --library-norm-method = geometric). Only those values with a valid status were selected. The cumulative fraction depending on fold change (log_2_ ratio) relative to control was analyzed as described previously (18). Cumulative fraction analyses depending on fold change (log_2_ ratio) were performed only for coding transcripts (RefSeq IDs start with NM) and were analyzed, where the high-confidence miR-124 sites were used to select miR-124-dependent transcripts. KS tests were performed by using Scipy (scipy.stats.ks_2samp()). The Fastq files can be accessed through our project website (http://clip.korea.ac.kr/n2a-124/).

### Construction of luciferase reporters

To measure the on-target activity, the psiCheck-2 vector (Promega) was used. In the 3′-UTR of synthetic Renilla luciferase, we inserted a perfect match site for measuring on-target repression. In general, synthetic duplex oligos (Bioneer, Korea) containing various target sites (siE6/E7, UAACCUGUGUAUAUUGCAA; siE6/E7-d, GCAGAGAAACACAAGUAUA; PCS-B1 or B2, UUCUAGACCUGUUUUGCUU; siE6, GUGCCAGAAACCGUUAAU; siA-E6-1, CAGACUCUGUGUAUGGAGA; siA-E6-2, GAA-ACCGUUGAAUCCAGCA; siA-E6-7, CCAGAAACCGUUGAAUCCA) were cloned into the psiCheck-2 plasmid through XhoI and NotI sites. For measuring off-target repression, two-seed match site (position 2–8) of PCS-B1 or B2 (UCUAGACUCUAGAC) is inserted into psiCheck-2 vector. siE6/E7 was previously developed as a therapeutic siRNA treating cervical cancer (27).

### Luciferase reporter assays

The efficiency of on-target activity was estimated by measuring IC_50_ with luciferase reporter assays as described previously (18). In brief, psiCheck-2 plasmids (Promega) were co-transfected with duplexed siRNAs by using Lipofectamine 2000 (Invitrogen). Twenty-four hours after transfection into HeLa cells, relative activity (Renilla luciferase activity normalized to firefly luciferase) was measured by Dual-Luciferase Reporter Assay System (Promega) with the GloMax-Multi Detection System (Promega) with replicates (*n* = 6) according to the manufacturer’s protocol. In general, half inhibitory concentration (IC_50_) was calculated by performing nonlinear least squares fitting for the sigmoid function using Scipy (scipy.optimize.curve_fit()). To examine the extent of preserving on-target activity, relative activity of the unmodified siRNA (WT) was calculated by comparing with IC_50_ values after applying abasic pivot substitution (6Ø) and represented as log ratio (log_2_(WT/6Ø)).

## Results

### The stability of transitional nucleation correlated with the target affinity of RNAi

Functional miRNA–target interaction triggering RNAi has been proposed to undergo ‘transitional nucleation’ (15), an initiation mode mediated by base pairs from position 2 to 6 in miRNA, with targets (Figure 1A). Especially, base pairing with position 6 of miRNA, named ‘pivot’, seems to be crucial because of discovering noncanonical targets forms a bulge, where nucleotide in the bulge (position 5–6) should be competent to pair with pivot (Supplementary Figure S1A) (15). We therefore hypothesized that the free energy of transitional nucleation (ΔG[2:6]) may correlate with binding affinity between miRNA and the target sites (Figure 1A).

To investigate this, miRNA–target interactions from Ago HITS-CLIP (14) were analyzed (Supplementary Table S1), where considering any factor that affects the target recognition should increase the correlation of sequencing frequency between Ago-associated miRNA and target sites. Initially, Ago HITS-CLIP data from mouse brain were examined by taking ΔG[2:6] into consideration (Figure 1B–D). As results, the numbers of seed target sites (seed) were shown to become more correlated with the expression level of corresponding miRNAs (*R^2^* = 0.34 vs. 0.61, Figure 1C–D). The improvement was significant, showing a 0.27 increment in correlation (*R^2^*), relative to considering the free energy of the entire sequences (ΔG_t_; no increment in *R^2^*). We also observed the same results with total target sites (total), which took both seed and noncanonical nucleation bulge sites (nuc) into consideration (Figure 1B and Supplementary Figure S2). The increment of *R^2^* was also shown in other Ago HITS-CLIP data, derived from 293S cells (0.15 increment, Figure 1E–F) and human brain (0.11 increment, Figure 1G–H), albeit their *R^2^* values were generally lower than the one from mouse brain. Overall, we confirmed that the stability of transitional nucleation is an important feature that correlates with target affinity of RNAi.

### The potency of siRNA-6Ø required a range of stability of transitional nucleation

Although the on-target affinity of siRNA largely depended on transitional nucleation (ΔG[2:6]), in order to prevent miRNA-like off-target interactions, ΔG[2:6] should be impaired to prevent initiation of miRNA-like target recognition in the transitional nucleation model (Figure 1A and Supplementary Figure S1). For this, the base in the pivot was intentionally substituted with the abasic spacer (siRNA-6Ø), which functions like a nucleotide backbone with no base, resulting in disruption of pivot pairing (18) and further limiting the stability of transitional nucleation to ΔG[2:5] (Figure 2A). Since siRNA-6Ø has been demonstrated to eliminate miRNA-like off-target repression (18), we initially examined the effect of siRNA-6Ø on global miRNA-like repression by performing RNA-Seq experiments, validating with cumulative analysis that miR-124-dependent transcripts (seed vs. total, *P* < 0.01, KS test) became completely derepressed after introducing abasic pivot substitution to miR-124 (miR-124-6Ø; seed vs. total, *P* = 0.54, KS test) in neuroblastoma cell line, N2a (Figure 2B).

Nevertheless, siRNA-6Ø inevitably sacrificed on-target activity albeit variable depending on its sequence composition (conservation of on-target activity = ∼80–100%) (18). Indeed, siRNA-6Ø reduced the number of base pairs in transitional nucleation, limiting from 5 to 4 base pairs (positions 2–5). Thus, to exert on-target activity like the unmodified siRNA, siRNA-6Ø has to overcome this insufficiency presumably through compensatory base pairing (Figure 2A). Not all sequences in impaired transitional nucleation (position 2–5) could efficiently overcome the disadvantage of siRNA-6Ø, postulating to examine whether the conserved potency of siRNA-6Ø depended on ΔG[2:5] (Figure 2C). Initially, the relative activities of siRNA and siRNA-6Ø were compared based on experimentally determined on-target activity (IC_50_), measured by luciferase reporter assays (Supplementary Figure 3A-B) or reported by previous studies (18) (Supplementary Table S3). As results, ∼ −3.5 kcal mol^−1^ was revealed to be the crucial threshold classifying siRNA-6Øs into conserved and non-conserved ones (Figure 2C and Supplementary Figure 3C).

Since there were only limited number of experimental results (*n* = 6) available for siRNA-6Øs, we decided to confirm the observed cutoff value of transitional nucleation by examining miRNA sequences, to see whether their transitional nucleation sequences had the tendency to be evolutionally conserved within the same threshold of thermodynamic stability (Supplementary Figure S4A–B)—if certain stability of transitional nucleation was required for miRNAs to interact with targets, only the sequences of miRNA capable to exert the corresponding stability would be remained as evolutionally conserved in biological systems. Indeed, cumulative fraction analysis showed that broadly conserved miRNAs (*n* = 107) was significantly more enriched than that of all possible 5-mers (*P* < 0.01, KS test) depending on the free energy of transitional nucleation (ΔG[2:6]), especially evident within a specific range, − 6 ≤ ΔG[2:6] ≤ −3.5 kcal mol^−1^ (Figure 2D). Intriguingly, miRNAs with the extremely stable transitional nucleation (ΔG[2:6] < −6 kcal mol^−1^) exhibited to be less enriched (Figure 2D), showing the range rather than the cutoff.

To confirm this observation, large-scale siRNA screening results (23) were utilized by examining high-confidence siRNAs (*n* = 322, Supplementary Table S4), which were selected to exclude compounding effects from selective Ago loading based on the previous strand bias study (the siRNAs with U in position 1 and lower ΔG[2:6] from antisense strand than that from sense strand) (24). Similarly, the high-confidence siRNAs (*n* = 322) showed less potent on-target activity (normalized inhibitory activity) when their transitional nucleation became too stable (ΔG[2:6] < −7 kcal mol^−1^) (Figure 2E). Moreover, additional nine high-confidence siRNAs (Supplementary Table S5), experimentally proven to have superior on-target activity (normalized inhibitory activity >0.7) in the study of siRNA potency (25), were all no more stable than this threshold of transitional nucleation (−7 kcal mol^−1^) (Supplementary Figure S5A). Expanded to a larger siRNA screen set, compiled siRNAs (*n* = 563) derived from 13 different publications (19) were analyzed for their potency on suppression of reporter activity, similarly observing the loss of suppression when free energy of transitional nucleation (ΔG[2:6]) was more stable than −8 kcal mol^−1^ (Figure 2F). High-confidence results of the compiled siRNA (*n* = 176, Supplementary Table S6) also supported the same cutoff value (Supplementary Figure S5B).

Although siRNA-6Ø has only 4 nt for transitional nucleation, 94 4-mer sequences (potent 4-mers, Supplementary Table S7) enabled to afford free energy of transitional nucleation in this discovered range (Figure 2G and Supplementary Figure S4C). Notably, most 4-mers with free energy less than −6 kcal mol^−1^, at which we also observed the reduced potency in the analyses of siRNA screening data (Figure 2E-F), were composed of GC stretches implicated in causing problems of target repression and specificity (5–7) (Figure 2G). Taken together, we were able to finally demonstrate that the free energy of transitional nucleation in siRNA-6Ø prefers the range of stability −6 ≤ ΔG[2:5] ≤ −3.5 kcal mol^−1^ for effective on-target repression.

### Designing of potent siRNA-6Ø sequences by siAbasic

**Figure 3:**
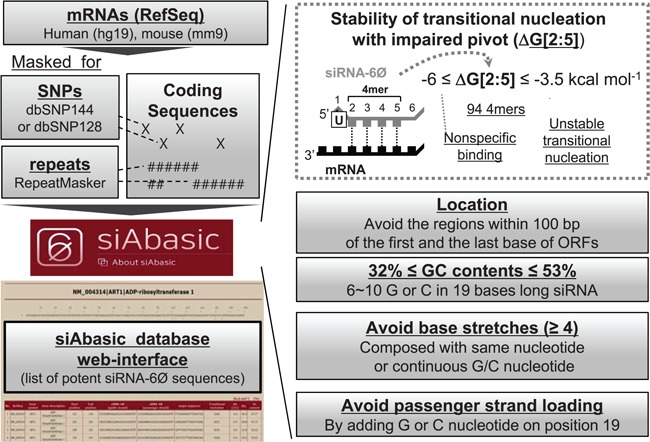
siAbasic designed potent siRNA-6Ø sequences**.** The workflow of siAbasic database is represented. The siAbasic designed potent siRNA-6Ø sequences targeting mRNAs in human and mouse genome. The results were offered through siAbasic database web-interface. Details are described in Materials and Methods section and in Supplementary Figure S6.

From all possible combinations of nucleation sequences in siRNA-6Ø (position 2–5; 4-mers, 4^4^ = 256), 94 sequences were selected as potent 4-mers (Figure 2G and Supplementary Table S7) and used in siAbasic to design potent siRNA-6Ø sequences (Figure 3A). To enhance the efficiency and specificity, siAbasic also took into consideration known general features for siRNA: (i) U in position 1 for efficient loading onto Ago (7, 2); (ii) avoidance of targeting to SNPs; dbSNP144 and dbSNP128 (4) or repeat sequences (RepeatMasker) (6); (iii) exclusion of the region within 100 bp of the first or last nucleotide of the ORFs (5); and (iv) no nucleotide stretches, including GC stretches (≥4 nt) (5–7), but with appropriate GC content (32–53%, 6–10 G or C in the total length) (Figure 3A and Supplementary Figure S6).

siAbasic generated comprehensive lists of potent siRNA-6Øs: 3 421 929 sequences targeting 18 472 genes in humans (95.8%) and 2 704 726 sequences for 19 728 genes in mice (95.4%). It provides search functionality (Supplementary Figure S7A) for each specified gene (official gene symbol or RefSeq accession number), returning candidate siRNA-6Øs as diagrams indicating the location of target sites in coding sequences. It also offers a table with sorting options, providing the location, siRNA-6Ø sequences, sequence of transitional nucleation, ΔG[2:5], ΔG_t_ and GC content (Figure 3B and Supplementary Figure S7B). Users are informed to decide further cutoff values for their purpose of search considering estimated on-target potency depending on the free energy value of transitional nucleation (Figure 2C–G). Therefore, users can further narrow down potentially effective siRNA-6Ø sequences for their interesting genes. In addition, siAbasic offers functionality of searching siRNA-6Ø sequences in their own sequences, possibly used to design siRNA-6Ø sequences for targeting a gene in species other than human and mouse (Supplementary Figure S7C). Using advanced options for this, users also can freely design siRNA-6Ø sequences by changing targeting location, range of ΔG[2:5], length of base or GC stretches and GC content.

### Validation of siRNA-6Øs designed by siAbasic

**Figure 4:**
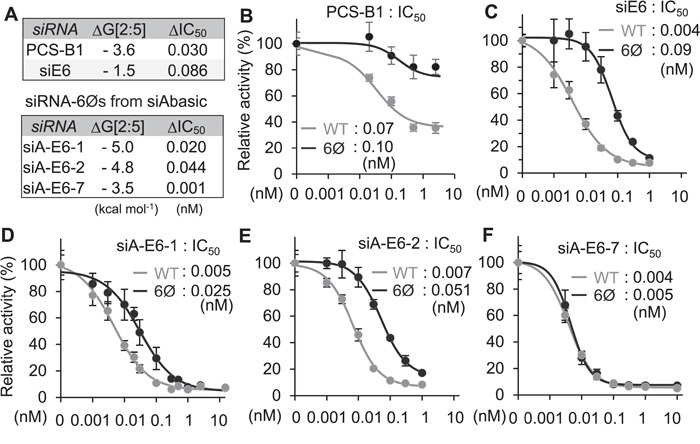
Experimental validation of siRNA-6Ø. (A) Validated list of siRNAs. (B–F) Repressive potency (IC_50_) was measured for siRNA-6Ø targeting PCSK9 (B), or HPV18 E6/E7 mRNA designed by the conventional method (4) (C) or by siAbasic (D–F).

To validate siAbasic and its potential therapeutic applications, the stability of transitional nucleation was examined for siRNA-6Ø against PCSK9 (PCS-B1, −3.6 kcal mol^−1^), developed for treating hypercholesterolemia (Figure 4A and Supplementary Table S8A) (28). As results of luciferase reporter assays, the elimination of miRNA-like off-target repression was confirmed for its therapeutic usage form (PCS-B2-6Ø; Supplementary Figure S8). Furthermore, by performing luciferase reporter assays, the expected conservation of on-target activity was further validated for PCS-B1 (ΔIC_50_ = 0.030, unmodified (WT) = 0.07 vs. 6Ø = 0.10 nM; Figure 4A and B). However, when siRNA-6Ø targeting E6/E7 mRNA of the human papillomavirus type 18 (siE6) was designed by a conventional method (4) (Supplementary Table S8A), it exhibited unstable transitional nucleation (−1.5 kcal mol^−1^; Figure 4A), which resulted in insufficient maintenance of on-target activity (ΔIC_50_ = 0.086, WT = 0.004 vs. 6Ø = 0.09 nM; Figure 4C). Finally, siAbasic was applied to design new siRNA-6Øs against E6/E7 mRNA (siA-E6; Supplementary Table S8B), and their expected on-target potencies were also experimentally validated as conserved (ΔIC_50_ ≤ 0.044) in two siRNA-6Øs from the top list (siA-E6-1, siA-E6-2) and one from the bottom (siA-E6-7) (Figure 4A, 4D–F and Supplementary Table S8B). Overall, we could demonstrate that siRNAs designed by siAbasic enabled to exert potent on-target activity, which is superior enough to be used for therapeutic purposes.

## Discussion

RNAi has been widely used as a method to repress expression of a desired target gene, but it also causes non-specific repression of other genes as an inevitable disadvantage, raising the serious concern of leading to faulty research results or side effects in therapeutic treatments. Such off-target effects have occurred since Ago protein treats siRNA, which is artificially introduced in order to induce RNAi, as an endogeneously expressed miRNA in a cell. The miRNA recognizes a target gene majorly through base pairing with a seed region (positions 2–7 from the 5′ end) for suppression, and the off targets caused by siRNAs are also induced depending on sequences of the seed regions as well (Supplementary Figure S1A). To avoid off-target effects, several methods including bioinformatics and chemical modifications have been applied to the seed region, but the effects were limited or dramatically sacrificed on-target activity.

Nevertheless, based on the model of seed-mediated miRNA–target interaction, which requires transitional nucleation (base pairs from position 2 to the pivot with targets, Figure 1A), abasic pivot substitution has been recently developed to destabilize base pair in pivot by introducing abasic nucleotide to siRNA (siRNA-6Ø), achieving complete elimination of miRNA-like off-target repression (0%) while preserving superior on-target activity (~80–100%) (Supplementary Figure S1B and Figure 2A). The transitional nucleation majorly functions in miRNA-target recognition; its thermodynamic stability constantly improves the correlation between abundance of miRNA and corresponding target sites in all Ago HITS-CLIP data analyzed here (results from mouse brain (14), 293S (19) and human heart (21); Figure 1). Because it is important for recognizing both off targets and an on target (Supplementary Figure 1A), defect in transitional nucleation of siRNA-6Ø not only results in abrogating miRNA-like off-target repression, but also inevitably sacrificing on-target activity by limiting the number of base pairs to only 4 (position 2–5).

To minimize this defect, impaired nucleation region (position 2–5) of siRNA-6Ø should be competent to be overcome by other consecutive pairing with on target. But, there was no information about the extent of thermodynamic stability required in transitional nucleation. By integrating results from siRNA screening, luciferase reporter assays and comparative sequence analysis of conserved miRNA sequences, we revealed the range of stability required in transitional nucleation (−6 ≤ ΔG[2:6] ≤ −3.5 kcal mol^−1^) to exhibit potent on-target activity (Figure 2). Direct comparison of siRNA activity through luciferase reporter assays between absence vs. presence of abasic pivot substitution (siRNA-6Ø) showed that ∼ −3.5 kcal mol^−1^ served as threshold. Consistent with these results, all of large scale siRNA results continuously showed the same cutoff value. Moreover, sequences of miRNAs comprising transitional nucleation regions (position 2–6) were evolutionally conserved evidently throughout the discovered range of free energies. Unexpectedly, extremely stable transitional nucleation (ΔG[2:6] < −6 kcal mol^−1^) were observed to be less potent, where majority of their sequences consisted of GC stretches that were reported to cause problems of target suppression (5–7) presumably due to affecting strand bias or harboring low complexity in the sequences. More importantly, substantial number of 4-mer sequences (*n* = 94) can afford thermodynamic stability in this range, fortunately enough to cover most of genes in human and mouse in combination with other criteria generally used in siRNA design (2–6). Thus, siAbasic database and its web-interface were able to be established and offer siRNA-6Ø sequences with guaranteed specificity (Figure 3). Moreover, the siRNA-6Øs, designed by siAbasic, were experimentally confirmed to have conserved on-target activity and further validated for their therapeutic potency (Figure 4).

Pre-existing methods or programs, developed for designing conventional siRNAs, have no option to consider defective pivot pairing in siRNA-6Ø, expecting their inferior performance for determining siRNA-6Ø sequences due to untold crucial changes in seed pairing. In support, when abasic pivot substitution was applied to therapeutic siRNA targeting E6/E7 mRNA (siE6), which had been designed by conventional strategy (4) for clinical purpose, siE6-6Ø showed deficiency in on-target activity compared with the unmodified (siE6, Figure 4C). Thus, although abasic pivot substitution has been developed and available for avoiding off-target effects, uncertainty of potent sequences and inability to apply general methods to designing sequences of siRNA-6Ø prohibited the usage of siRNA-6Ø with concern. In contrast, all siRNAs designed by siAbasic showed efficient preservation of on-target activity (Figure 4D–F). siAbasic also adopted most of critical and general criteria, which have been established for designing siRNAs in other methods, but the uniqueness of siAbasic is that it takes care not only the extent of transitional nucleation, but also the shortening of transitional nucleation pairing (4 base pairs; position 2–5). By preselecting candidates of sequences in impaired transitional nucleation (positions 2–5) that accommodated the discovered range of potent stability (−6 ≤ ΔG[2:5] ≤ −3.5 kcal mol^−1^), siAbasic easily enabled to determine list of siRNAs targeting most of genes in mouse and human genome. Of note, siAbasic is specifically developed for determining siRNA-6Ø sequences, not designed to use for general siRNA without abasic modification. Because we suspected that there will be not so much need to design conventional siRNAs, by using siAbasic, siRNA-6Ø sequences could be easily designed and widely used with guaranteed potency and no off-target effects.

In summary, here we provide a comprehensive list of potentially effective siRNAs with abasic pivot substitution (siRNA-6Øs) majorly considering stability of transitional nucleation, which have not been offered by any other current approach for designing siRNAs. By discovering the range of stability of transitional nucleation that allows potent on-target repression, we can provide list of potentially effective siRNAs with abasic pivot substitution (siRNA-6Øs). Because siRNA-6Ø completely abrogates off-target effects (with 0% off-target repression), optimized on-target potency designed by siAbasic ensures target specificity. siAbasic will facilitate the use of siRNA-6Ø for silencing target genes with assured efficiency and specificity, assisting the study of genes for loss of function and the development of therapeutics for various diseases.

## Supplementary Material

Supplementary Data

## Notes

**Database URL**: http://clip.korea.ac.kr/siabasic/

## References

[1] Hannon G.J. and Rossi J.J. (2004) Unlocking the potential of the human genome with RNA interference. Nature, 431, 371–378. 15372045 10.1038/nature02870

[2] Seok H. et al. (2016) MicroRNA target recognition: insights from transcriptome-wide non-canonical interactions. Mol. Cells, 39, 375–381. 27117456 10.14348/molcells.2016.0013 PMC4870184

[3] Bottini S. et al. (2017) Recent computational developments on CLIP-seq data analysis and microRNA targeting implications. Brief Bioinform., bbx063, 10.1093/bib/bbx063. PMC6291801 28605404

[4] Yuan B. et al. (2004) siRNA Selection Server: an automated siRNA oligonucleotide prediction server. Nucleic Acids Res., 32, W130–W134. 15215365 10.1093/nar/gkh366 PMC441504

[5] Elbashir S.M. et al. (2002) Analysis of gene function in somatic mammalian cells using small interfering RNAs. Methods, 26, 199–213. 12054897 10.1016/S1046-2023(02)00023-3

[6] Reynolds A. et al. (2004) Rational siRNA design for RNA interference. Nat. Biotechnol., 22, 326–330. 14758366 10.1038/nbt936

[7] Ui-Tei K. et al. (2004) Guidelines for the selection of highly effective siRNA sequences for mammalian and chick RNA interference. Nucleic Acids Res., 32, 936–948. 14769950 10.1093/nar/gkh247 PMC373388

[8] Jackson A.L. and Linsley P.S. (2010) Recognizing and avoiding siRNA off-target effects for target identification and therapeutic application. Nat. Rev. Drug Discov., 9, 57–67. 20043028 10.1038/nrd3010

[9] Birmingham A. et al. (2007) A protocol for designing siRNAs with high functionality and specificity. Nat. Protoc., 2, 2068–2078. 17853862 10.1038/nprot.2007.278

[10] Alkan F. et al. (2017) RIsearch2: suffix array-based large-scale prediction of RNA–RNA interactions and siRNA off-targets. Nucleic Acids Res., 45, e60. 28108657 10.1093/nar/gkw1325 PMC5416843

[11] Bramsen J.B. et al. (2009) A large-scale chemical modification screen identifies design rules to generate siRNAs with high activity, high stability and low toxicity. Nucleic Acids Res, 37, 2867–2881. 19282453 10.1093/nar/gkp106 PMC2685080

[12] Jackson A.L. et al. (2006) Position-specific chemical modification of siRNAs reduces `off-target' transcript silencing. RNA, 12, 1197–1205. 16682562 10.1261/rna.30706 PMC1484422

[13] Seok H. et al. (2017) Evaluation and control of miRNA-like off-target repression for RNA interference. Cell. Mol. Life Sci., 12, 797–814. 10.1007/s00018-017-2656-0 PMC11105550 28905147

[14] Chi S.W. et al. (2009) Argonaute HITS-CLIP decodes microRNA–mRNA interaction maps. Nature, 460, 479–486. 19536157 10.1038/nature08170 PMC2733940

[15] Chi S.W., Hannon G.J. and Darnell R.B. (2012) An alternative mode of microRNA target recognition. Nat. Struct. Mol. Biol., 19, 321–327. 22343717 10.1038/nsmb.2230 PMC3541676

[16] Kim K.K., Ham J. and Chi S.W. (2013) miRTCat: a comprehensive map of human and mouse microRNA target sites including non-canonical nucleation bulges. Bioinformatics, 29, 1898–1899. 23709495 10.1093/bioinformatics/btt296

[17] Seok H., Jang E.S. and Chi S.W. (2016) Rationally designed siRNAs without miRNA-like off-target repression. BMB Rep., 49, 135–136. 26839153 10.5483/BMBRep.2016.49.3.019 PMC4915226

[18] Lee H.S. et al. (2015) Abasic pivot substitution harnesses target specificity of RNA interference. Nat. Commun., 6, 10154. 10.1038/ncomms10154 PMC4703836 26679372

[19] Karginov F.V. and Hannon G.J. (2013) Remodeling of Ago2-mRNA interactions upon cellular stress reflects miRNA complementarity and correlates with altered translation rates. Genes Dev., 27, 1624–1632. 23824327 10.1101/gad.215939.113 PMC3731550

[20] Li J.H. et al. (2014) starBase v2.0: decoding miRNA–ceRNA, miRNA–ncRNA and protein–RNA interaction networks from large-scale CLIP-Seq data. Nucleic Acids Res., 42, D92–D97. 24297251 10.1093/nar/gkt1248 PMC3964941

[21] Spengler R.M. et al. (2016) Elucidation of transcriptome-wide microRNA binding sites in human cardiac tissues by Ago2 HITS-CLIP. Nucleic Acids Res., 44, 7120–7131. 27418678 10.1093/nar/gkw640 PMC5009757

[22] Lorenz R. et al. (2011) ViennaRNA Package 2.0. Algorithms Mol. Biol., 6, 26. 22115189 10.1186/1748-7188-6-26 PMC3319429

[23] Huesken D. et al. (2005) Design of a genome-wide siRNA library using an artificial neural network. Nat. Biotechnol., 23, 995–1001. 16025102 10.1038/nbt1118

[24] Khvorova A., Reynolds A. and Jayasena S.D. (2003) Functional siRNAs and miRNAs exhibit strand bias. Cell, 115, 209–216. 14567918 10.1016/s0092-8674(03)00801-8

[25] Ui-Tei K. et al. (2008) Thermodynamic stability and Watson–Crick base pairing in the seed duplex are major determinants of the efficiency of the siRNA-based off-target effect. Nucleic Acids Res., 36, 7100–7109. 18988625 10.1093/nar/gkn902 PMC2602766

[26] Shabalina S.A., Spiridonov A.N. and Ogurtsov A.Y. (2006) Computational models with thermodynamic and composition features improve siRNA design. BMC Bioinformatics, 7, 65. 16472402 10.1186/1471-2105-7-65 PMC1431570

[27] Jung H.S. et al. (2012) The synergistic therapeutic effect of cisplatin with Human papillomavirus E6/E7 short interfering RNA on cervical cancer cell lines in vitro and in vivo. Int. J. Cancer, 130, 1925–1936. 21630254 10.1002/ijc.26197

[28] Frank-Kamenetsky M. et al. (2008) Therapeutic RNAi targeting PCSK9 acutely lowers plasma cholesterol in rodents and LDL cholesterol in nonhuman primates. Proc. Natl. Acad. Sci. U. S. A., 105, 11915–11920. 18695239 10.1073/pnas.0805434105 PMC2575310

