# Supplementary material for: siAbasic: a comprehensive database for potent siRNA-6Ø sequences without off-target effects
Source: Database (Oxford). 2018 Oct 12;2018:bay109. doi: 10.1093/database/bay109 (PMC6183071; doi:10.1093/database/bay109)
Supplement: Supplementary Data [file supplementary_figures_090518_jyp_chi_new2.pptx]

## Slide 1
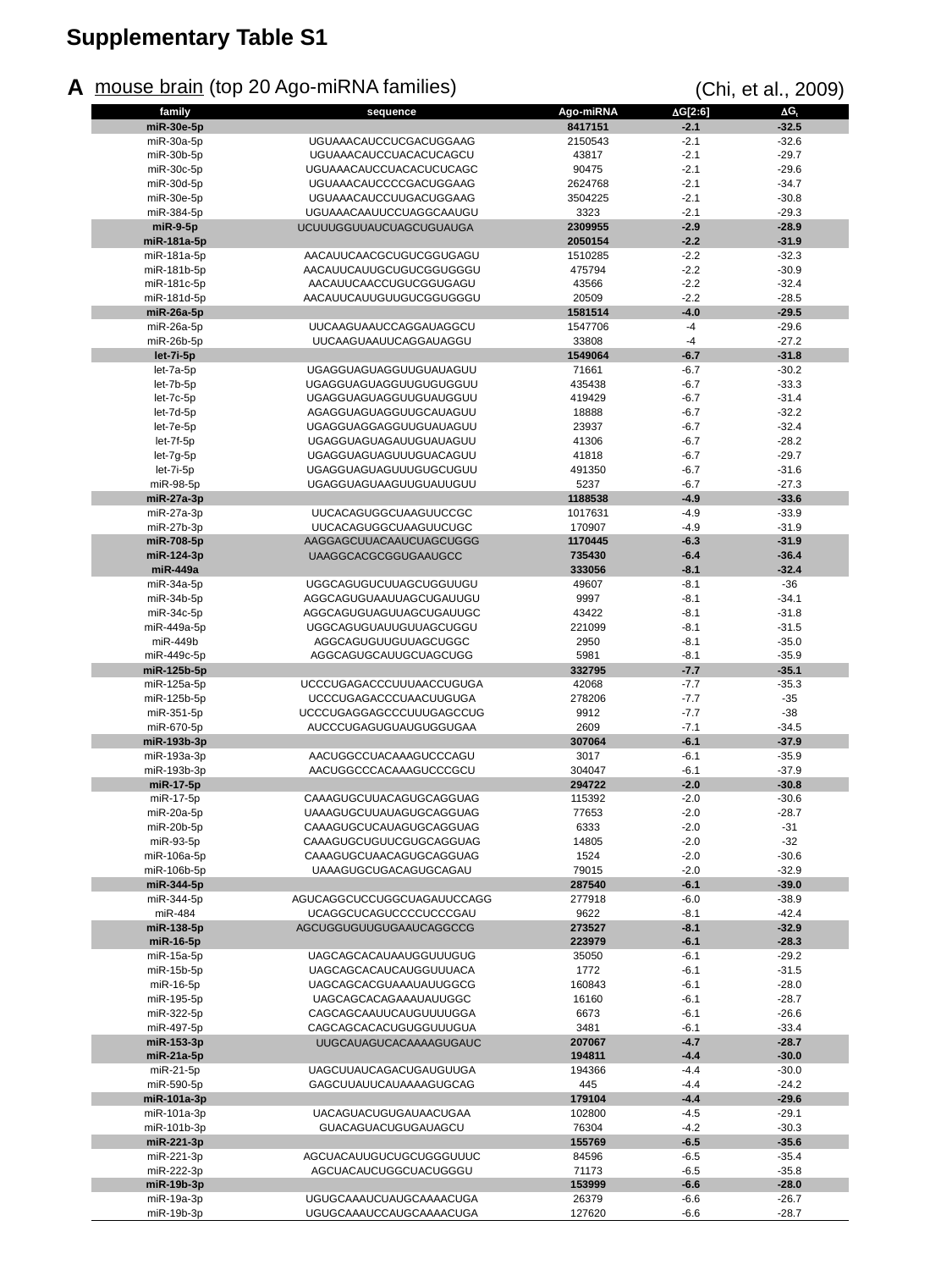

Supplementary Table S1
A
mouse brain (top 20 Ago-miRNA families)
(Chi, et al., 2009)
| family | sequence | Ago-miRNA | DG[2:6] | DGt |
| --- | --- | --- | --- | --- |
| miR-30e-5p | | 8417151 | -2.1 | -32.5 |
| miR-30a-5p | UGUAAACAUCCUCGACUGGAAG | 2150543 | -2.1 | -32.6 |
| miR-30b-5p | UGUAAACAUCCUACACUCAGCU | 43817 | -2.1 | -29.7 |
| miR-30c-5p | UGUAAACAUCCUACACUCUCAGC | 90475 | -2.1 | -29.6 |
| miR-30d-5p | UGUAAACAUCCCCGACUGGAAG | 2624768 | -2.1 | -34.7 |
| miR-30e-5p | UGUAAACAUCCUUGACUGGAAG | 3504225 | -2.1 | -30.8 |
| miR-384-5p | UGUAAACAAUUCCUAGGCAAUGU | 3323 | -2.1 | -29.3 |
| miR-9-5p | UCUUUGGUUAUCUAGCUGUAUGA | 2309955 | -2.9 | -28.9 |
| miR-181a-5p | | 2050154 | -2.2 | -31.9 |
| miR-181a-5p | AACAUUCAACGCUGUCGGUGAGU | 1510285 | -2.2 | -32.3 |
| miR-181b-5p | AACAUUCAUUGCUGUCGGUGGGU | 475794 | -2.2 | -30.9 |
| miR-181c-5p | AACAUUCAACCUGUCGGUGAGU | 43566 | -2.2 | -32.4 |
| miR-181d-5p | AACAUUCAUUGUUGUCGGUGGGU | 20509 | -2.2 | -28.5 |
| miR-26a-5p | | 1581514 | -4.0 | -29.5 |
| miR-26a-5p | UUCAAGUAAUCCAGGAUAGGCU | 1547706 | -4 | -29.6 |
| miR-26b-5p | UUCAAGUAAUUCAGGAUAGGU | 33808 | -4 | -27.2 |
| let-7i-5p | | 1549064 | -6.7 | -31.8 |
| let-7a-5p | UGAGGUAGUAGGUUGUAUAGUU | 71661 | -6.7 | -30.2 |
| let-7b-5p | UGAGGUAGUAGGUUGUGUGGUU | 435438 | -6.7 | -33.3 |
| let-7c-5p | UGAGGUAGUAGGUUGUAUGGUU | 419429 | -6.7 | -31.4 |
| let-7d-5p | AGAGGUAGUAGGUUGCAUAGUU | 18888 | -6.7 | -32.2 |
| let-7e-5p | UGAGGUAGGAGGUUGUAUAGUU | 23937 | -6.7 | -32.4 |
| let-7f-5p | UGAGGUAGUAGAUUGUAUAGUU | 41306 | -6.7 | -28.2 |
| let-7g-5p | UGAGGUAGUAGUUUGUACAGUU | 41818 | -6.7 | -29.7 |
| let-7i-5p | UGAGGUAGUAGUUUGUGCUGUU | 491350 | -6.7 | -31.6 |
| miR-98-5p | UGAGGUAGUAAGUUGUAUUGUU | 5237 | -6.7 | -27.3 |
| miR-27a-3p | | 1188538 | -4.9 | -33.6 |
| miR-27a-3p | UUCACAGUGGCUAAGUUCCGC | 1017631 | -4.9 | -33.9 |
| miR-27b-3p | UUCACAGUGGCUAAGUUCUGC | 170907 | -4.9 | -31.9 |
| miR-708-5p | AAGGAGCUUACAAUCUAGCUGGG | 1170445 | -6.3 | -31.9 |
| miR-124-3p | UAAGGCACGCGGUGAAUGCC | 735430 | -6.4 | -36.4 |
| miR-449a | | 333056 | -8.1 | -32.4 |
| miR-34a-5p | UGGCAGUGUCUUAGCUGGUUGU | 49607 | -8.1 | -36 |
| miR-34b-5p | AGGCAGUGUAAUUAGCUGAUUGU | 9997 | -8.1 | -34.1 |
| miR-34c-5p | AGGCAGUGUAGUUAGCUGAUUGC | 43422 | -8.1 | -31.8 |
| miR-449a-5p | UGGCAGUGUAUUGUUAGCUGGU | 221099 | -8.1 | -31.5 |
| miR-449b | AGGCAGUGUUGUUAGCUGGC | 2950 | -8.1 | -35.0 |
| miR-449c-5p | AGGCAGUGCAUUGCUAGCUGG | 5981 | -8.1 | -35.9 |
| miR-125b-5p | | 332795 | -7.7 | -35.1 |
| miR-125a-5p | UCCCUGAGACCCUUUAACCUGUGA | 42068 | -7.7 | -35.3 |
| miR-125b-5p | UCCCUGAGACCCUAACUUGUGA | 278206 | -7.7 | -35 |
| miR-351-5p | UCCCUGAGGAGCCCUUUGAGCCUG | 9912 | -7.7 | -38 |
| miR-670-5p | AUCCCUGAGUGUAUGUGGUGAA | 2609 | -7.1 | -34.5 |
| miR-193b-3p | | 307064 | -6.1 | -37.9 |
| miR-193a-3p | AACUGGCCUACAAAGUCCCAGU | 3017 | -6.1 | -35.9 |
| miR-193b-3p | AACUGGCCCACAAAGUCCCGCU | 304047 | -6.1 | -37.9 |
| miR-17-5p | | 294722 | -2.0 | -30.8 |
| miR-17-5p | CAAAGUGCUUACAGUGCAGGUAG | 115392 | -2.0 | -30.6 |
| miR-20a-5p | UAAAGUGCUUAUAGUGCAGGUAG | 77653 | -2.0 | -28.7 |
| miR-20b-5p | CAAAGUGCUCAUAGUGCAGGUAG | 6333 | -2.0 | -31 |
| miR-93-5p | CAAAGUGCUGUUCGUGCAGGUAG | 14805 | -2.0 | -32 |
| miR-106a-5p | CAAAGUGCUAACAGUGCAGGUAG | 1524 | -2.0 | -30.6 |
| miR-106b-5p | UAAAGUGCUGACAGUGCAGAU | 79015 | -2.0 | -32.9 |
| miR-344-5p | | 287540 | -6.1 | -39.0 |
| miR-344-5p | AGUCAGGCUCCUGGCUAGAUUCCAGG | 277918 | -6.0 | -38.9 |
| miR-484 | UCAGGCUCAGUCCCCUCCCGAU | 9622 | -8.1 | -42.4 |
| miR-138-5p | AGCUGGUGUUGUGAAUCAGGCCG | 273527 | -8.1 | -32.9 |
| miR-16-5p | | 223979 | -6.1 | -28.3 |
| miR-15a-5p | UAGCAGCACAUAAUGGUUUGUG | 35050 | -6.1 | -29.2 |
| miR-15b-5p | UAGCAGCACAUCAUGGUUUACA | 1772 | -6.1 | -31.5 |
| miR-16-5p | UAGCAGCACGUAAAUAUUGGCG | 160843 | -6.1 | -28.0 |
| miR-195-5p | UAGCAGCACAGAAAUAUUGGC | 16160 | -6.1 | -28.7 |
| miR-322-5p | CAGCAGCAAUUCAUGUUUUGGA | 6673 | -6.1 | -26.6 |
| miR-497-5p | CAGCAGCACACUGUGGUUUGUA | 3481 | -6.1 | -33.4 |
| miR-153-3p | UUGCAUAGUCACAAAAGUGAUC | 207067 | -4.7 | -28.7 |
| miR-21a-5p | | 194811 | -4.4 | -30.0 |
| miR-21-5p | UAGCUUAUCAGACUGAUGUUGA | 194366 | -4.4 | -30.0 |
| miR-590-5p | GAGCUUAUUCAUAAAAGUGCAG | 445 | -4.4 | -24.2 |
| miR-101a-3p | | 179104 | -4.4 | -29.6 |
| miR-101a-3p | UACAGUACUGUGAUAACUGAA | 102800 | -4.5 | -29.1 |
| miR-101b-3p | GUACAGUACUGUGAUAGCU | 76304 | -4.2 | -30.3 |
| miR-221-3p | | 155769 | -6.5 | -35.6 |
| miR-221-3p | AGCUACAUUGUCUGCUGGGUUUC | 84596 | -6.5 | -35.4 |
| miR-222-3p | AGCUACAUCUGGCUACUGGGU | 71173 | -6.5 | -35.8 |
| miR-19b-3p | | 153999 | -6.6 | -28.0 |
| miR-19a-3p | UGUGCAAAUCUAUGCAAAACUGA | 26379 | -6.6 | -26.7 |
| miR-19b-3p | UGUGCAAAUCCAUGCAAAACUGA | 127620 | -6.6 | -28.7 |

## Slide 2
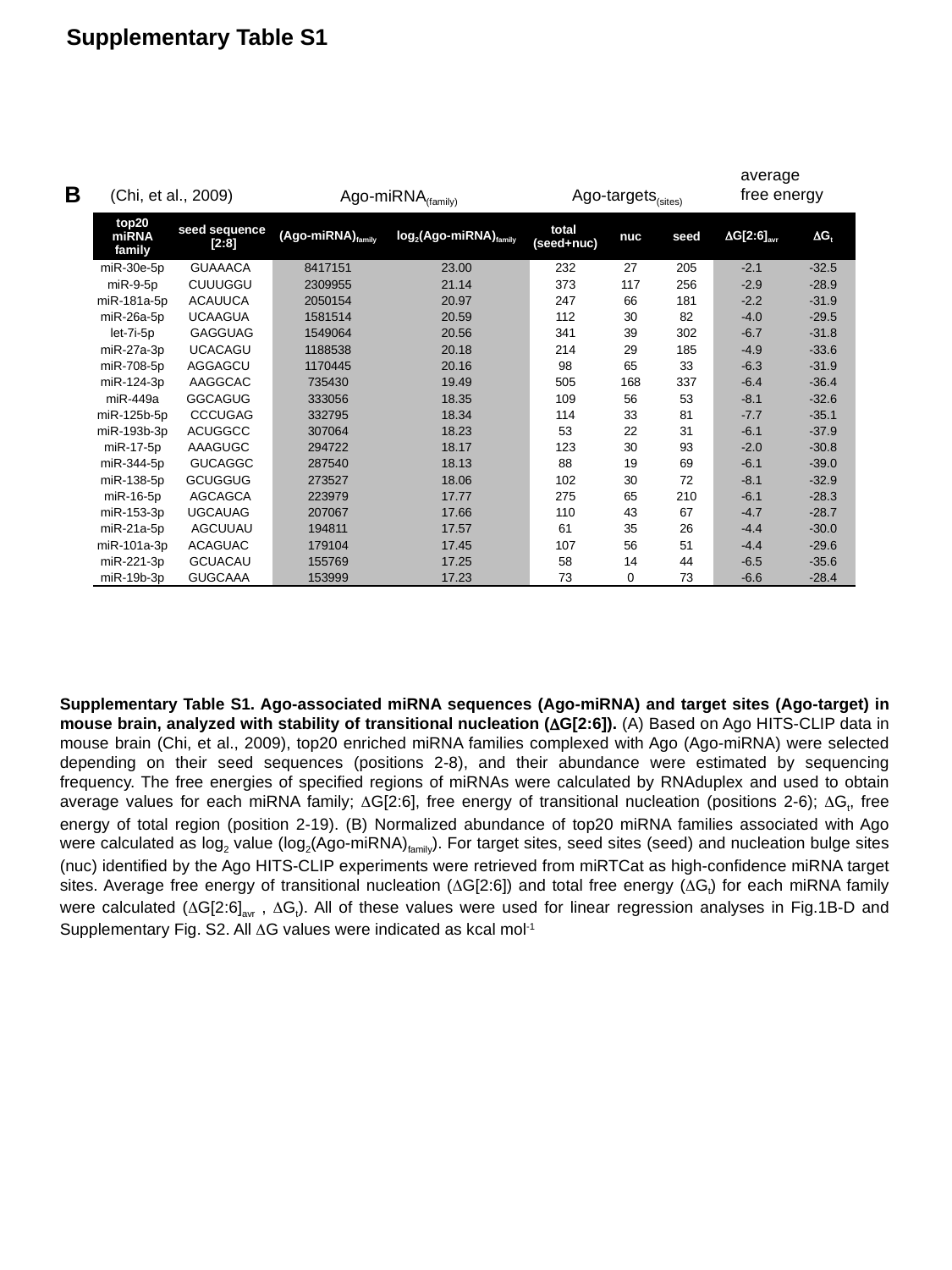

Supplementary Table S1
average free energy
B
Ago-targets(sites)
Ago-miRNA(family)
(Chi, et al., 2009)
| top20 miRNA family | seed sequence [2:8] | (Ago-miRNA)family | log2(Ago-miRNA)family | total (seed+nuc) | nuc | seed | DG[2:6]avr | DGt |
| --- | --- | --- | --- | --- | --- | --- | --- | --- |
| miR-30e-5p | GUAAACA | 8417151 | 23.00 | 232 | 27 | 205 | -2.1 | -32.5 |
| miR-9-5p | CUUUGGU | 2309955 | 21.14 | 373 | 117 | 256 | -2.9 | -28.9 |
| miR-181a-5p | ACAUUCA | 2050154 | 20.97 | 247 | 66 | 181 | -2.2 | -31.9 |
| miR-26a-5p | UCAAGUA | 1581514 | 20.59 | 112 | 30 | 82 | -4.0 | -29.5 |
| let-7i-5p | GAGGUAG | 1549064 | 20.56 | 341 | 39 | 302 | -6.7 | -31.8 |
| miR-27a-3p | UCACAGU | 1188538 | 20.18 | 214 | 29 | 185 | -4.9 | -33.6 |
| miR-708-5p | AGGAGCU | 1170445 | 20.16 | 98 | 65 | 33 | -6.3 | -31.9 |
| miR-124-3p | AAGGCAC | 735430 | 19.49 | 505 | 168 | 337 | -6.4 | -36.4 |
| miR-449a | GGCAGUG | 333056 | 18.35 | 109 | 56 | 53 | -8.1 | -32.6 |
| miR-125b-5p | CCCUGAG | 332795 | 18.34 | 114 | 33 | 81 | -7.7 | -35.1 |
| miR-193b-3p | ACUGGCC | 307064 | 18.23 | 53 | 22 | 31 | -6.1 | -37.9 |
| miR-17-5p | AAAGUGC | 294722 | 18.17 | 123 | 30 | 93 | -2.0 | -30.8 |
| miR-344-5p | GUCAGGC | 287540 | 18.13 | 88 | 19 | 69 | -6.1 | -39.0 |
| miR-138-5p | GCUGGUG | 273527 | 18.06 | 102 | 30 | 72 | -8.1 | -32.9 |
| miR-16-5p | AGCAGCA | 223979 | 17.77 | 275 | 65 | 210 | -6.1 | -28.3 |
| miR-153-3p | UGCAUAG | 207067 | 17.66 | 110 | 43 | 67 | -4.7 | -28.7 |
| miR-21a-5p | AGCUUAU | 194811 | 17.57 | 61 | 35 | 26 | -4.4 | -30.0 |
| miR-101a-3p | ACAGUAC | 179104 | 17.45 | 107 | 56 | 51 | -4.4 | -29.6 |
| miR-221-3p | GCUACAU | 155769 | 17.25 | 58 | 14 | 44 | -6.5 | -35.6 |
| miR-19b-3p | GUGCAAA | 153999 | 17.23 | 73 | 0 | 73 | -6.6 | -28.4 |
Supplementary Table S1. Ago-associated miRNA sequences (Ago-miRNA) and target sites (Ago-target) in mouse brain, analyzed with stability of transitional nucleation (DG[2:6]). (A) Based on Ago HITS-CLIP data in mouse brain (Chi, et al., 2009), top20 enriched miRNA families complexed with Ago (Ago-miRNA) were selected depending on their seed sequences (positions 2-8), and their abundance were estimated by sequencing frequency. The free energies of specified regions of miRNAs were calculated by RNAduplex and used to obtain average values for each miRNA family; DG[2:6], free energy of transitional nucleation (positions 2-6); DGt, free energy of total region (position 2-19). (B) Normalized abundance of top20 miRNA families associated with Ago were calculated as log2 value (log2(Ago-miRNA)family). For target sites, seed sites (seed) and nucleation bulge sites (nuc) identified by the Ago HITS-CLIP experiments were retrieved from miRTCat as high-confidence miRNA target sites. Average free energy of transitional nucleation (DG[2:6]) and total free energy (DGt) for each miRNA family were calculated (DG[2:6]avr , DGt). All of these values were used for linear regression analyses in Fig.1B-D and Supplementary Fig. S2. All DG values were indicated as kcal mol-1

## Slide 3
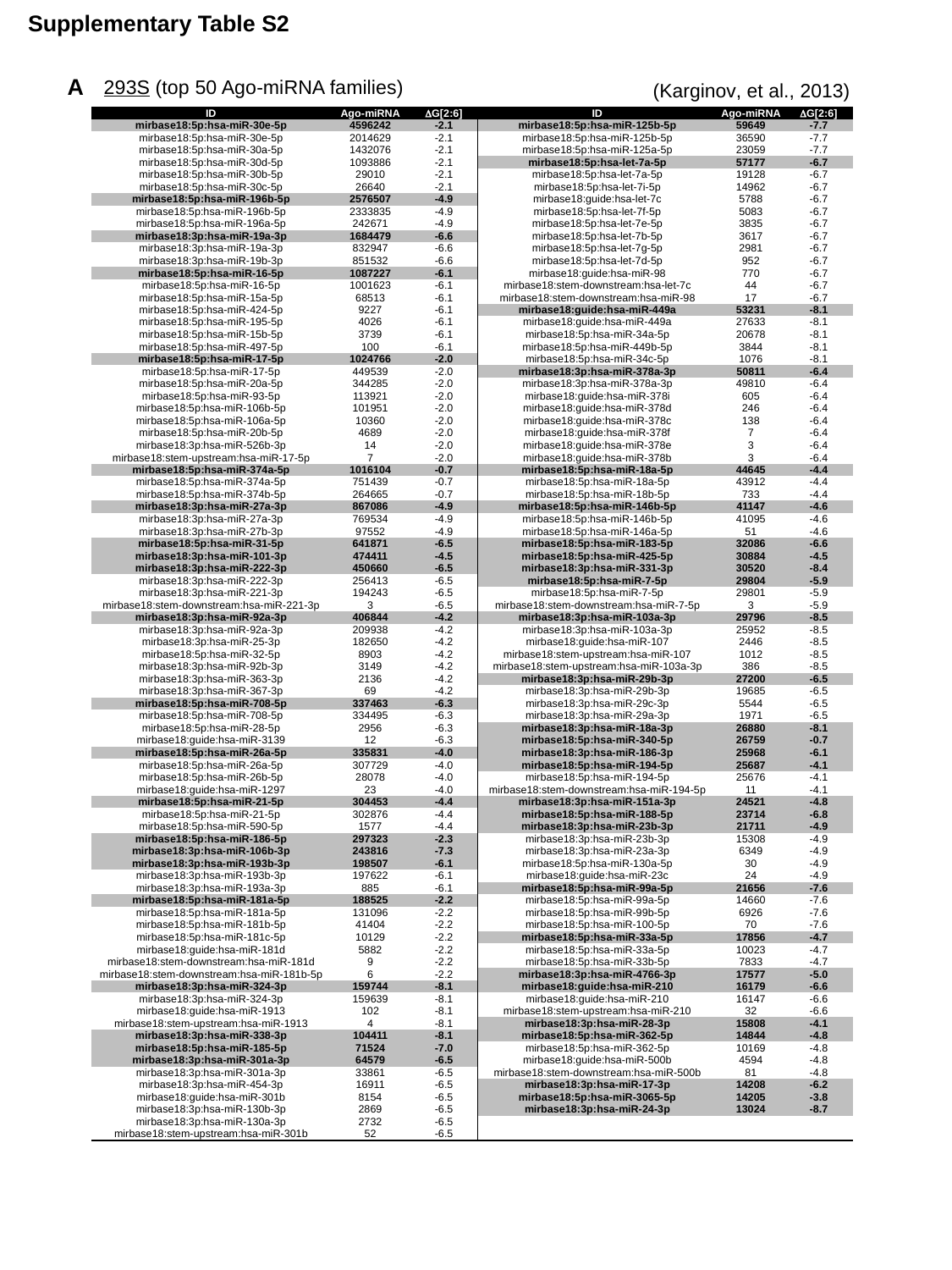

Supplementary Table S2
A
293S (top 50 Ago-miRNA families)
(Karginov, et al., 2013)
| ID | Ago-miRNA | DG[2:6] | ID | Ago-miRNA | DG[2:6] |
| --- | --- | --- | --- | --- | --- |
| mirbase18:5p:hsa-miR-30e-5p | 4596242 | -2.1 | mirbase18:5p:hsa-miR-125b-5p | 59649 | -7.7 |
| mirbase18:5p:hsa-miR-30e-5p | 2014629 | -2.1 | mirbase18:5p:hsa-miR-125b-5p | 36590 | -7.7 |
| mirbase18:5p:hsa-miR-30a-5p | 1432076 | -2.1 | mirbase18:5p:hsa-miR-125a-5p | 23059 | -7.7 |
| mirbase18:5p:hsa-miR-30d-5p | 1093886 | -2.1 | mirbase18:5p:hsa-let-7a-5p | 57177 | -6.7 |
| mirbase18:5p:hsa-miR-30b-5p | 29010 | -2.1 | mirbase18:5p:hsa-let-7a-5p | 19128 | -6.7 |
| mirbase18:5p:hsa-miR-30c-5p | 26640 | -2.1 | mirbase18:5p:hsa-let-7i-5p | 14962 | -6.7 |
| mirbase18:5p:hsa-miR-196b-5p | 2576507 | -4.9 | mirbase18:guide:hsa-let-7c | 5788 | -6.7 |
| mirbase18:5p:hsa-miR-196b-5p | 2333835 | -4.9 | mirbase18:5p:hsa-let-7f-5p | 5083 | -6.7 |
| mirbase18:5p:hsa-miR-196a-5p | 242671 | -4.9 | mirbase18:5p:hsa-let-7e-5p | 3835 | -6.7 |
| mirbase18:3p:hsa-miR-19a-3p | 1684479 | -6.6 | mirbase18:5p:hsa-let-7b-5p | 3617 | -6.7 |
| mirbase18:3p:hsa-miR-19a-3p | 832947 | -6.6 | mirbase18:5p:hsa-let-7g-5p | 2981 | -6.7 |
| mirbase18:3p:hsa-miR-19b-3p | 851532 | -6.6 | mirbase18:5p:hsa-let-7d-5p | 952 | -6.7 |
| mirbase18:5p:hsa-miR-16-5p | 1087227 | -6.1 | mirbase18:guide:hsa-miR-98 | 770 | -6.7 |
| mirbase18:5p:hsa-miR-16-5p | 1001623 | -6.1 | mirbase18:stem-downstream:hsa-let-7c | 44 | -6.7 |
| mirbase18:5p:hsa-miR-15a-5p | 68513 | -6.1 | mirbase18:stem-downstream:hsa-miR-98 | 17 | -6.7 |
| mirbase18:5p:hsa-miR-424-5p | 9227 | -6.1 | mirbase18:guide:hsa-miR-449a | 53231 | -8.1 |
| mirbase18:5p:hsa-miR-195-5p | 4026 | -6.1 | mirbase18:guide:hsa-miR-449a | 27633 | -8.1 |
| mirbase18:5p:hsa-miR-15b-5p | 3739 | -6.1 | mirbase18:5p:hsa-miR-34a-5p | 20678 | -8.1 |
| mirbase18:5p:hsa-miR-497-5p | 100 | -6.1 | mirbase18:5p:hsa-miR-449b-5p | 3844 | -8.1 |
| mirbase18:5p:hsa-miR-17-5p | 1024766 | -2.0 | mirbase18:5p:hsa-miR-34c-5p | 1076 | -8.1 |
| mirbase18:5p:hsa-miR-17-5p | 449539 | -2.0 | mirbase18:3p:hsa-miR-378a-3p | 50811 | -6.4 |
| mirbase18:5p:hsa-miR-20a-5p | 344285 | -2.0 | mirbase18:3p:hsa-miR-378a-3p | 49810 | -6.4 |
| mirbase18:5p:hsa-miR-93-5p | 113921 | -2.0 | mirbase18:guide:hsa-miR-378i | 605 | -6.4 |
| mirbase18:5p:hsa-miR-106b-5p | 101951 | -2.0 | mirbase18:guide:hsa-miR-378d | 246 | -6.4 |
| mirbase18:5p:hsa-miR-106a-5p | 10360 | -2.0 | mirbase18:guide:hsa-miR-378c | 138 | -6.4 |
| mirbase18:5p:hsa-miR-20b-5p | 4689 | -2.0 | mirbase18:guide:hsa-miR-378f | 7 | -6.4 |
| mirbase18:3p:hsa-miR-526b-3p | 14 | -2.0 | mirbase18:guide:hsa-miR-378e | 3 | -6.4 |
| mirbase18:stem-upstream:hsa-miR-17-5p | 7 | -2.0 | mirbase18:guide:hsa-miR-378b | 3 | -6.4 |
| mirbase18:5p:hsa-miR-374a-5p | 1016104 | -0.7 | mirbase18:5p:hsa-miR-18a-5p | 44645 | -4.4 |
| mirbase18:5p:hsa-miR-374a-5p | 751439 | -0.7 | mirbase18:5p:hsa-miR-18a-5p | 43912 | -4.4 |
| mirbase18:5p:hsa-miR-374b-5p | 264665 | -0.7 | mirbase18:5p:hsa-miR-18b-5p | 733 | -4.4 |
| mirbase18:3p:hsa-miR-27a-3p | 867086 | -4.9 | mirbase18:5p:hsa-miR-146b-5p | 41147 | -4.6 |
| mirbase18:3p:hsa-miR-27a-3p | 769534 | -4.9 | mirbase18:5p:hsa-miR-146b-5p | 41095 | -4.6 |
| mirbase18:3p:hsa-miR-27b-3p | 97552 | -4.9 | mirbase18:5p:hsa-miR-146a-5p | 51 | -4.6 |
| mirbase18:5p:hsa-miR-31-5p | 641871 | -6.5 | mirbase18:5p:hsa-miR-183-5p | 32086 | -6.6 |
| mirbase18:3p:hsa-miR-101-3p | 474411 | -4.5 | mirbase18:5p:hsa-miR-425-5p | 30884 | -4.5 |
| mirbase18:3p:hsa-miR-222-3p | 450660 | -6.5 | mirbase18:3p:hsa-miR-331-3p | 30520 | -8.4 |
| mirbase18:3p:hsa-miR-222-3p | 256413 | -6.5 | mirbase18:5p:hsa-miR-7-5p | 29804 | -5.9 |
| mirbase18:3p:hsa-miR-221-3p | 194243 | -6.5 | mirbase18:5p:hsa-miR-7-5p | 29801 | -5.9 |
| mirbase18:stem-downstream:hsa-miR-221-3p | 3 | -6.5 | mirbase18:stem-downstream:hsa-miR-7-5p | 3 | -5.9 |
| mirbase18:3p:hsa-miR-92a-3p | 406844 | -4.2 | mirbase18:3p:hsa-miR-103a-3p | 29796 | -8.5 |
| mirbase18:3p:hsa-miR-92a-3p | 209938 | -4.2 | mirbase18:3p:hsa-miR-103a-3p | 25952 | -8.5 |
| mirbase18:3p:hsa-miR-25-3p | 182650 | -4.2 | mirbase18:guide:hsa-miR-107 | 2446 | -8.5 |
| mirbase18:5p:hsa-miR-32-5p | 8903 | -4.2 | mirbase18:stem-upstream:hsa-miR-107 | 1012 | -8.5 |
| mirbase18:3p:hsa-miR-92b-3p | 3149 | -4.2 | mirbase18:stem-upstream:hsa-miR-103a-3p | 386 | -8.5 |
| mirbase18:3p:hsa-miR-363-3p | 2136 | -4.2 | mirbase18:3p:hsa-miR-29b-3p | 27200 | -6.5 |
| mirbase18:3p:hsa-miR-367-3p | 69 | -4.2 | mirbase18:3p:hsa-miR-29b-3p | 19685 | -6.5 |
| mirbase18:5p:hsa-miR-708-5p | 337463 | -6.3 | mirbase18:3p:hsa-miR-29c-3p | 5544 | -6.5 |
| mirbase18:5p:hsa-miR-708-5p | 334495 | -6.3 | mirbase18:3p:hsa-miR-29a-3p | 1971 | -6.5 |
| mirbase18:5p:hsa-miR-28-5p | 2956 | -6.3 | mirbase18:3p:hsa-miR-18a-3p | 26880 | -8.1 |
| mirbase18:guide:hsa-miR-3139 | 12 | -6.3 | mirbase18:5p:hsa-miR-340-5p | 26759 | -0.7 |
| mirbase18:5p:hsa-miR-26a-5p | 335831 | -4.0 | mirbase18:3p:hsa-miR-186-3p | 25968 | -6.1 |
| mirbase18:5p:hsa-miR-26a-5p | 307729 | -4.0 | mirbase18:5p:hsa-miR-194-5p | 25687 | -4.1 |
| mirbase18:5p:hsa-miR-26b-5p | 28078 | -4.0 | mirbase18:5p:hsa-miR-194-5p | 25676 | -4.1 |
| mirbase18:guide:hsa-miR-1297 | 23 | -4.0 | mirbase18:stem-downstream:hsa-miR-194-5p | 11 | -4.1 |
| mirbase18:5p:hsa-miR-21-5p | 304453 | -4.4 | mirbase18:3p:hsa-miR-151a-3p | 24521 | -4.8 |
| mirbase18:5p:hsa-miR-21-5p | 302876 | -4.4 | mirbase18:5p:hsa-miR-188-5p | 23714 | -6.8 |
| mirbase18:5p:hsa-miR-590-5p | 1577 | -4.4 | mirbase18:3p:hsa-miR-23b-3p | 21711 | -4.9 |
| mirbase18:5p:hsa-miR-186-5p | 297323 | -2.3 | mirbase18:3p:hsa-miR-23b-3p | 15308 | -4.9 |
| mirbase18:3p:hsa-miR-106b-3p | 243816 | -7.3 | mirbase18:3p:hsa-miR-23a-3p | 6349 | -4.9 |
| mirbase18:3p:hsa-miR-193b-3p | 198507 | -6.1 | mirbase18:5p:hsa-miR-130a-5p | 30 | -4.9 |
| mirbase18:3p:hsa-miR-193b-3p | 197622 | -6.1 | mirbase18:guide:hsa-miR-23c | 24 | -4.9 |
| mirbase18:3p:hsa-miR-193a-3p | 885 | -6.1 | mirbase18:5p:hsa-miR-99a-5p | 21656 | -7.6 |
| mirbase18:5p:hsa-miR-181a-5p | 188525 | -2.2 | mirbase18:5p:hsa-miR-99a-5p | 14660 | -7.6 |
| mirbase18:5p:hsa-miR-181a-5p | 131096 | -2.2 | mirbase18:5p:hsa-miR-99b-5p | 6926 | -7.6 |
| mirbase18:5p:hsa-miR-181b-5p | 41404 | -2.2 | mirbase18:5p:hsa-miR-100-5p | 70 | -7.6 |
| mirbase18:5p:hsa-miR-181c-5p | 10129 | -2.2 | mirbase18:5p:hsa-miR-33a-5p | 17856 | -4.7 |
| mirbase18:guide:hsa-miR-181d | 5882 | -2.2 | mirbase18:5p:hsa-miR-33a-5p | 10023 | -4.7 |
| mirbase18:stem-downstream:hsa-miR-181d | 9 | -2.2 | mirbase18:5p:hsa-miR-33b-5p | 7833 | -4.7 |
| mirbase18:stem-downstream:hsa-miR-181b-5p | 6 | -2.2 | mirbase18:3p:hsa-miR-4766-3p | 17577 | -5.0 |
| mirbase18:3p:hsa-miR-324-3p | 159744 | -8.1 | mirbase18:guide:hsa-miR-210 | 16179 | -6.6 |
| mirbase18:3p:hsa-miR-324-3p | 159639 | -8.1 | mirbase18:guide:hsa-miR-210 | 16147 | -6.6 |
| mirbase18:guide:hsa-miR-1913 | 102 | -8.1 | mirbase18:stem-upstream:hsa-miR-210 | 32 | -6.6 |
| mirbase18:stem-upstream:hsa-miR-1913 | 4 | -8.1 | mirbase18:3p:hsa-miR-28-3p | 15808 | -4.1 |
| mirbase18:3p:hsa-miR-338-3p | 104411 | -8.1 | mirbase18:5p:hsa-miR-362-5p | 14844 | -4.8 |
| mirbase18:5p:hsa-miR-185-5p | 71524 | -7.0 | mirbase18:5p:hsa-miR-362-5p | 10169 | -4.8 |
| mirbase18:3p:hsa-miR-301a-3p | 64579 | -6.5 | mirbase18:guide:hsa-miR-500b | 4594 | -4.8 |
| mirbase18:3p:hsa-miR-301a-3p | 33861 | -6.5 | mirbase18:stem-downstream:hsa-miR-500b | 81 | -4.8 |
| mirbase18:3p:hsa-miR-454-3p | 16911 | -6.5 | mirbase18:3p:hsa-miR-17-3p | 14208 | -6.2 |
| mirbase18:guide:hsa-miR-301b | 8154 | -6.5 | mirbase18:5p:hsa-miR-3065-5p | 14205 | -3.8 |
| mirbase18:3p:hsa-miR-130b-3p | 2869 | -6.5 | mirbase18:3p:hsa-miR-24-3p | 13024 | -8.7 |
| mirbase18:3p:hsa-miR-130a-3p | 2732 | -6.5 | | | |
| mirbase18:stem-upstream:hsa-miR-301b | 52 | -6.5 | | | |

## Slide 4
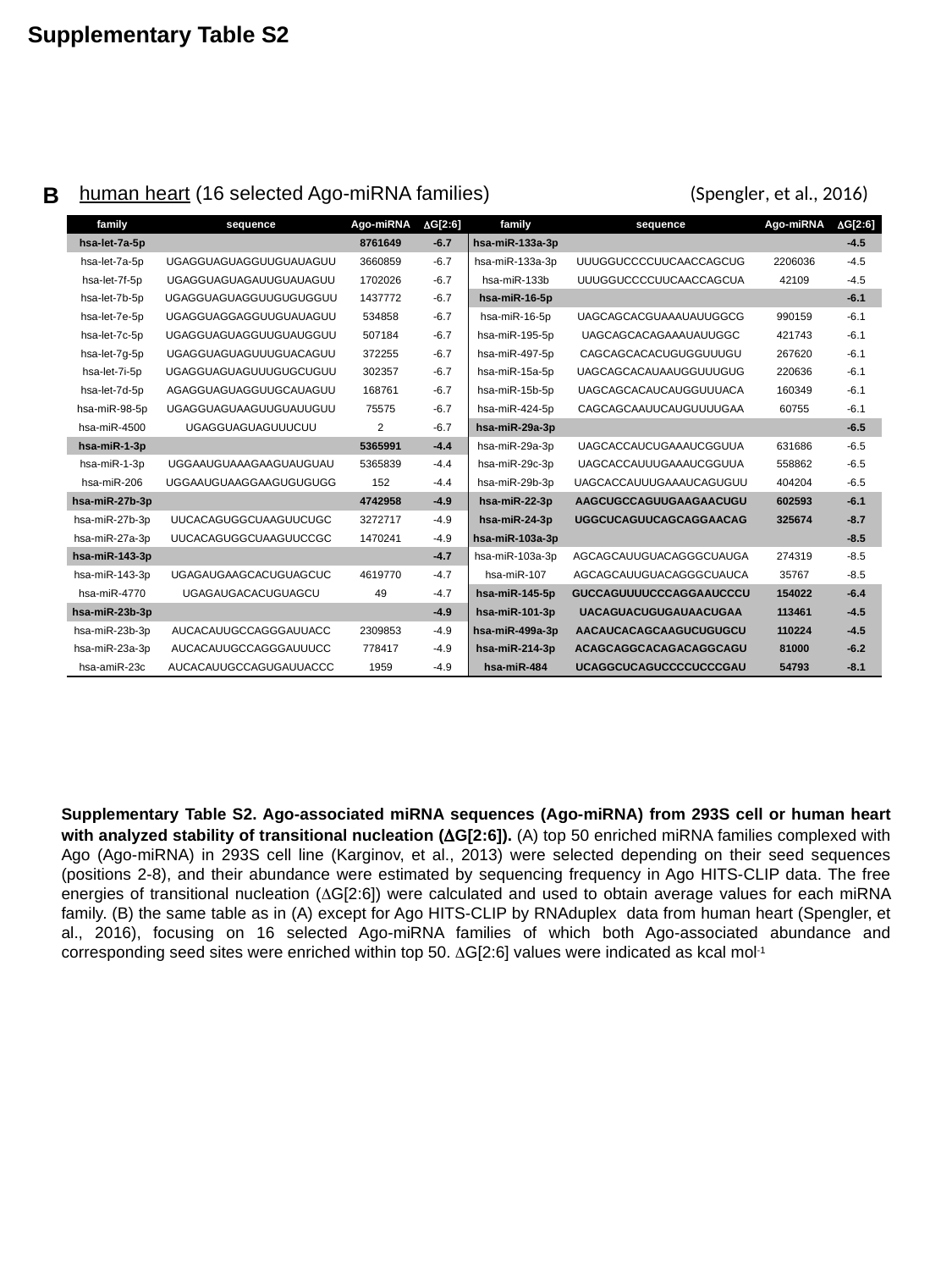

Supplementary Table S2
B
(Spengler, et al., 2016)
human heart (16 selected Ago-miRNA families)
| family | sequence | Ago-miRNA | DG[2:6] | family | sequence | Ago-miRNA | DG[2:6] |
| --- | --- | --- | --- | --- | --- | --- | --- |
| hsa-let-7a-5p | | 8761649 | -6.7 | hsa-miR-133a-3p | | | -4.5 |
| hsa-let-7a-5p | UGAGGUAGUAGGUUGUAUAGUU | 3660859 | -6.7 | hsa-miR-133a-3p | UUUGGUCCCCUUCAACCAGCUG | 2206036 | -4.5 |
| hsa-let-7f-5p | UGAGGUAGUAGAUUGUAUAGUU | 1702026 | -6.7 | hsa-miR-133b | UUUGGUCCCCUUCAACCAGCUA | 42109 | -4.5 |
| hsa-let-7b-5p | UGAGGUAGUAGGUUGUGUGGUU | 1437772 | -6.7 | hsa-miR-16-5p | | | -6.1 |
| hsa-let-7e-5p | UGAGGUAGGAGGUUGUAUAGUU | 534858 | -6.7 | hsa-miR-16-5p | UAGCAGCACGUAAAUAUUGGCG | 990159 | -6.1 |
| hsa-let-7c-5p | UGAGGUAGUAGGUUGUAUGGUU | 507184 | -6.7 | hsa-miR-195-5p | UAGCAGCACAGAAAUAUUGGC | 421743 | -6.1 |
| hsa-let-7g-5p | UGAGGUAGUAGUUUGUACAGUU | 372255 | -6.7 | hsa-miR-497-5p | CAGCAGCACACUGUGGUUUGU | 267620 | -6.1 |
| hsa-let-7i-5p | UGAGGUAGUAGUUUGUGCUGUU | 302357 | -6.7 | hsa-miR-15a-5p | UAGCAGCACAUAAUGGUUUGUG | 220636 | -6.1 |
| hsa-let-7d-5p | AGAGGUAGUAGGUUGCAUAGUU | 168761 | -6.7 | hsa-miR-15b-5p | UAGCAGCACAUCAUGGUUUACA | 160349 | -6.1 |
| hsa-miR-98-5p | UGAGGUAGUAAGUUGUAUUGUU | 75575 | -6.7 | hsa-miR-424-5p | CAGCAGCAAUUCAUGUUUUGAA | 60755 | -6.1 |
| hsa-miR-4500 | UGAGGUAGUAGUUUCUU | 2 | -6.7 | hsa-miR-29a-3p | | | -6.5 |
| hsa-miR-1-3p | | 5365991 | -4.4 | hsa-miR-29a-3p | UAGCACCAUCUGAAAUCGGUUA | 631686 | -6.5 |
| hsa-miR-1-3p | UGGAAUGUAAAGAAGUAUGUAU | 5365839 | -4.4 | hsa-miR-29c-3p | UAGCACCAUUUGAAAUCGGUUA | 558862 | -6.5 |
| hsa-miR-206 | UGGAAUGUAAGGAAGUGUGUGG | 152 | -4.4 | hsa-miR-29b-3p | UAGCACCAUUUGAAAUCAGUGUU | 404204 | -6.5 |
| hsa-miR-27b-3p | | 4742958 | -4.9 | hsa-miR-22-3p | AAGCUGCCAGUUGAAGAACUGU | 602593 | -6.1 |
| hsa-miR-27b-3p | UUCACAGUGGCUAAGUUCUGC | 3272717 | -4.9 | hsa-miR-24-3p | UGGCUCAGUUCAGCAGGAACAG | 325674 | -8.7 |
| hsa-miR-27a-3p | UUCACAGUGGCUAAGUUCCGC | 1470241 | -4.9 | hsa-miR-103a-3p | | | -8.5 |
| hsa-miR-143-3p | | | -4.7 | hsa-miR-103a-3p | AGCAGCAUUGUACAGGGCUAUGA | 274319 | -8.5 |
| hsa-miR-143-3p | UGAGAUGAAGCACUGUAGCUC | 4619770 | -4.7 | hsa-miR-107 | AGCAGCAUUGUACAGGGCUAUCA | 35767 | -8.5 |
| hsa-miR-4770 | UGAGAUGACACUGUAGCU | 49 | -4.7 | hsa-miR-145-5p | GUCCAGUUUUCCCAGGAAUCCCU | 154022 | -6.4 |
| hsa-miR-23b-3p | | | -4.9 | hsa-miR-101-3p | UACAGUACUGUGAUAACUGAA | 113461 | -4.5 |
| hsa-miR-23b-3p | AUCACAUUGCCAGGGAUUACC | 2309853 | -4.9 | hsa-miR-499a-3p | AACAUCACAGCAAGUCUGUGCU | 110224 | -4.5 |
| hsa-miR-23a-3p | AUCACAUUGCCAGGGAUUUCC | 778417 | -4.9 | hsa-miR-214-3p | ACAGCAGGCACAGACAGGCAGU | 81000 | -6.2 |
| hsa-amiR-23c | AUCACAUUGCCAGUGAUUACCC | 1959 | -4.9 | hsa-miR-484 | UCAGGCUCAGUCCCCUCCCGAU | 54793 | -8.1 |
Supplementary Table S2. Ago-associated miRNA sequences (Ago-miRNA) from 293S cell or human heart with analyzed stability of transitional nucleation (DG[2:6]). (A) top 50 enriched miRNA families complexed with Ago (Ago-miRNA) in 293S cell line (Karginov, et al., 2013) were selected depending on their seed sequences (positions 2-8), and their abundance were estimated by sequencing frequency in Ago HITS-CLIP data. The free energies of transitional nucleation (DG[2:6]) were calculated and used to obtain average values for each miRNA family. (B) the same table as in (A) except for Ago HITS-CLIP by RNAduplex data from human heart (Spengler, et al., 2016), focusing on 16 selected Ago-miRNA families of which both Ago-associated abundance and corresponding seed sites were enriched within top 50. DG[2:6] values were indicated as kcal mol-1

## Slide 5
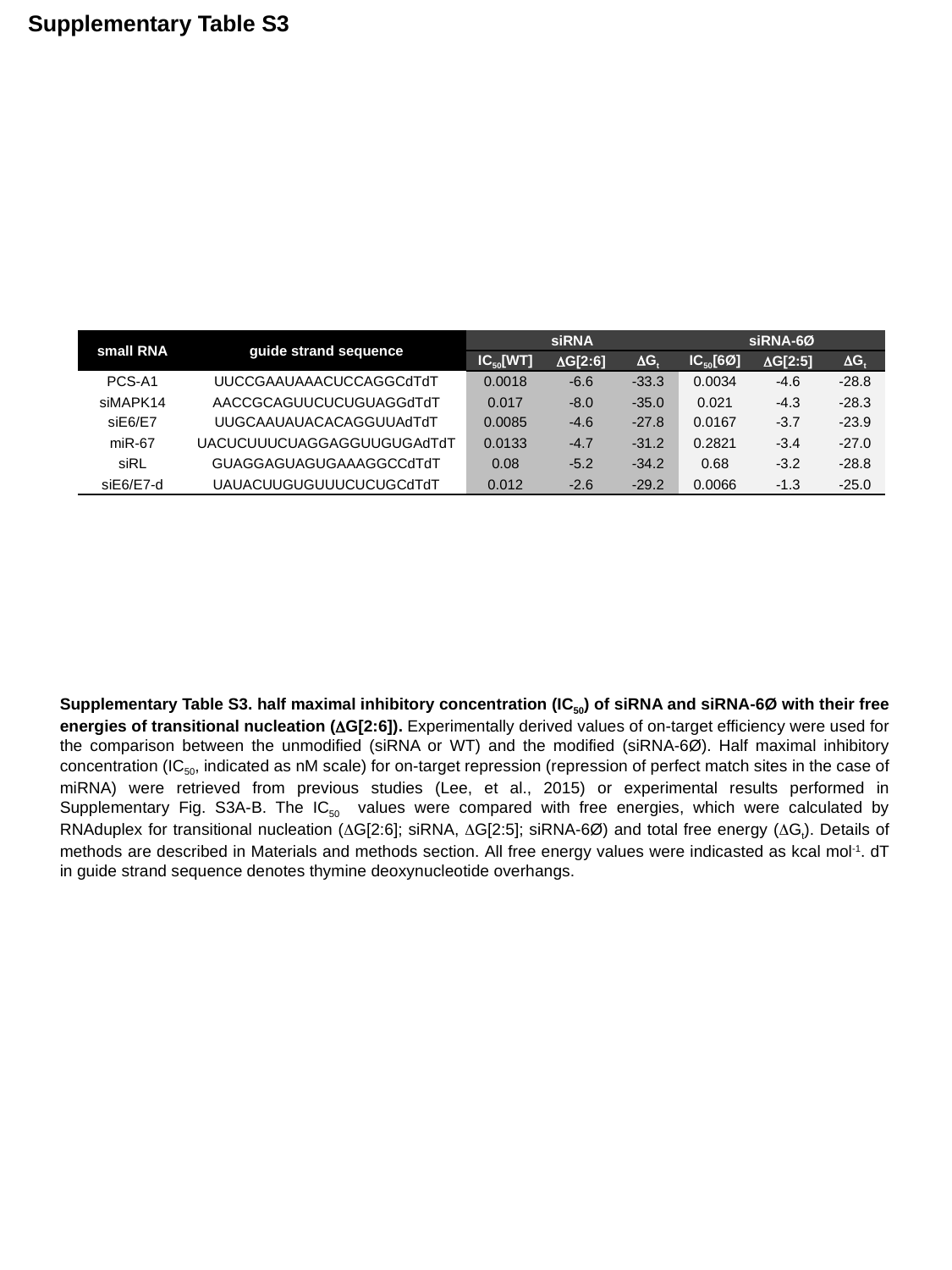

Supplementary Table S3
| small RNA | guide strand sequence | siRNA | | | siRNA-6Ø | | |
| --- | --- | --- | --- | --- | --- | --- | --- |
| | | IC50[WT] | DG[2:6] | DGt | IC50[6Ø] | DG[2:5] | DGt |
| PCS-A1 | UUCCGAAUAAACUCCAGGCdTdT | 0.0018 | -6.6 | -33.3 | 0.0034 | -4.6 | -28.8 |
| siMAPK14 | AACCGCAGUUCUCUGUAGGdTdT | 0.017 | -8.0 | -35.0 | 0.021 | -4.3 | -28.3 |
| siE6/E7 | UUGCAAUAUACACAGGUUAdTdT | 0.0085 | -4.6 | -27.8 | 0.0167 | -3.7 | -23.9 |
| miR-67 | UACUCUUUCUAGGAGGUUGUGAdTdT | 0.0133 | -4.7 | -31.2 | 0.2821 | -3.4 | -27.0 |
| siRL | GUAGGAGUAGUGAAAGGCCdTdT | 0.08 | -5.2 | -34.2 | 0.68 | -3.2 | -28.8 |
| siE6/E7-d | UAUACUUGUGUUUCUCUGCdTdT | 0.012 | -2.6 | -29.2 | 0.0066 | -1.3 | -25.0 |
Supplementary Table S3. half maximal inhibitory concentration (IC50) of siRNA and siRNA-6Ø with their free energies of transitional nucleation (DG[2:6]). Experimentally derived values of on-target efficiency were used for the comparison between the unmodified (siRNA or WT) and the modified (siRNA-6Ø). Half maximal inhibitory concentration (IC50, indicated as nM scale) for on-target repression (repression of perfect match sites in the case of miRNA) were retrieved from previous studies (Lee, et al., 2015) or experimental results performed in Supplementary Fig. S3A-B. The IC50 values were compared with free energies, which were calculated by RNAduplex for transitional nucleation (DG[2:6]; siRNA, DG[2:5]; siRNA-6Ø) and total free energy (DGt). Details of methods are described in Materials and methods section. All free energy values were indicasted as kcal mol-1. dT in guide strand sequence denotes thymine deoxynucleotide overhangs.

## Slide 6
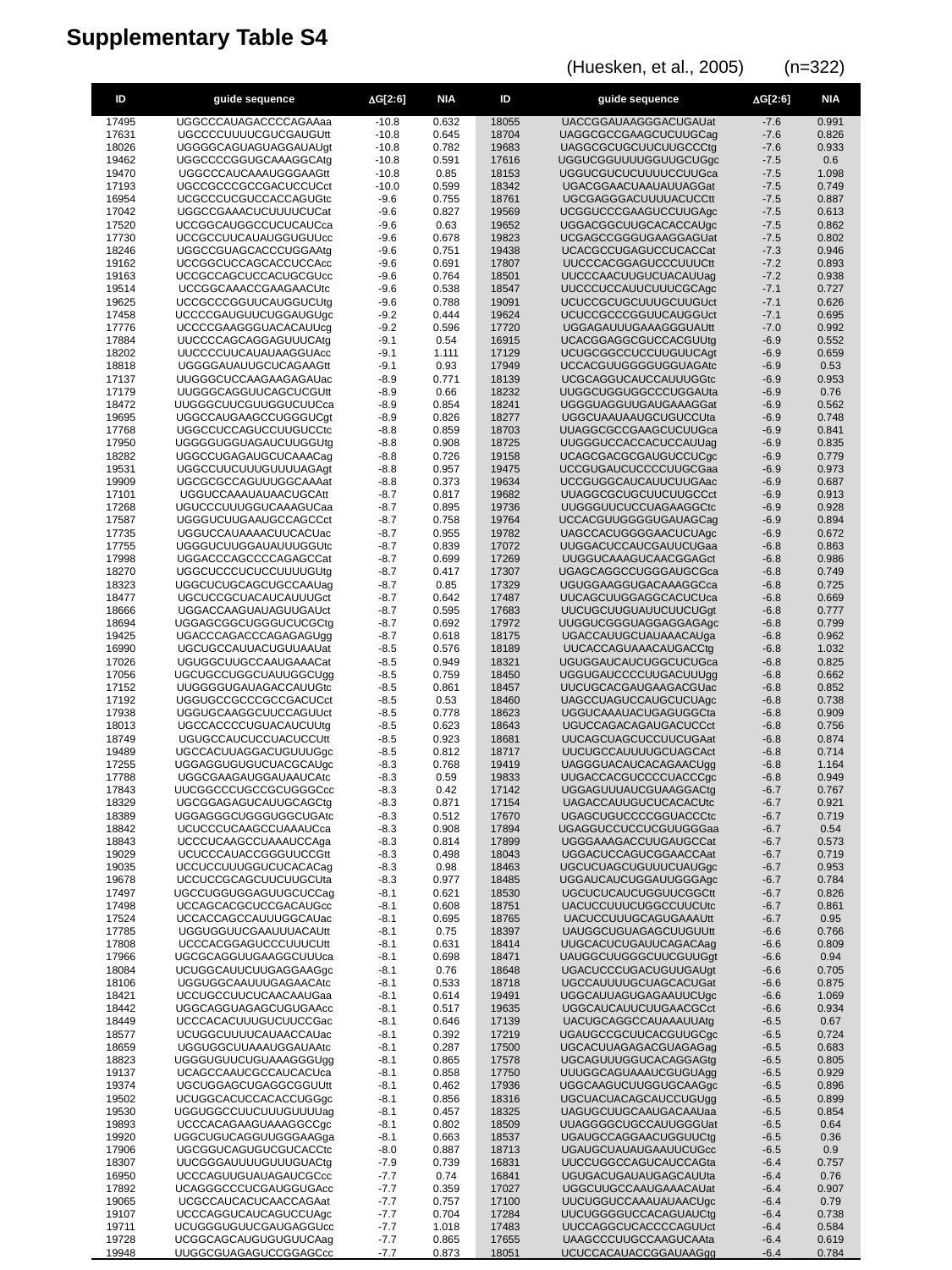

Supplementary Table S4
(Huesken, et al., 2005)
(n=322)
| ID | guide sequence | DG[2:6] | NIA | ID | guide sequence | DG[2:6] | NIA |
| --- | --- | --- | --- | --- | --- | --- | --- |
| 17495 | UGGCCCAUAGACCCCAGAAaa | -10.8 | 0.632 | 18055 | UACCGGAUAAGGGACUGAUat | -7.6 | 0.991 |
| 17631 | UGCCCCUUUUCGUCGAUGUtt | -10.8 | 0.645 | 18704 | UAGGCGCCGAAGCUCUUGCag | -7.6 | 0.826 |
| 18026 | UGGGGCAGUAGUAGGAUAUgt | -10.8 | 0.782 | 19683 | UAGGCGCUGCUUCUUGCCCtg | -7.6 | 0.933 |
| 19462 | UGGCCCCGGUGCAAAGGCAtg | -10.8 | 0.591 | 17616 | UGGUCGGUUUUGGUUGCUGgc | -7.5 | 0.6 |
| 19470 | UGGCCCAUCAAAUGGGAAGtt | -10.8 | 0.85 | 18153 | UGGUCGUCUCUUUUCCUUGca | -7.5 | 1.098 |
| 17193 | UGCCGCCCGCCGACUCCUCct | -10.0 | 0.599 | 18342 | UGACGGAACUAAUAUUAGGat | -7.5 | 0.749 |
| 16954 | UCGCCCUCGUCCACCAGUGtc | -9.6 | 0.755 | 18761 | UGCGAGGGACUUUUACUCCtt | -7.5 | 0.887 |
| 17042 | UGGCCGAAACUCUUUUCUCat | -9.6 | 0.827 | 19569 | UCGGUCCCGAAGUCCUUGAgc | -7.5 | 0.613 |
| 17520 | UCCGGCAUGGCCUCUCAUCca | -9.6 | 0.63 | 19652 | UGGACGGCUUGCACACCAUgc | -7.5 | 0.862 |
| 17730 | UCCGCCUUCAUAUGGUGUUcc | -9.6 | 0.678 | 19823 | UCGAGCCGGGUGAAGGAGUat | -7.5 | 0.802 |
| 18246 | UGGCCGUAGCACCCUGGAAtg | -9.6 | 0.751 | 19438 | UCACGCCUGAGUCCUCACCat | -7.3 | 0.946 |
| 19162 | UCCGGCUCCAGCACCUCCAcc | -9.6 | 0.691 | 17807 | UUCCCACGGAGUCCCUUUCtt | -7.2 | 0.893 |
| 19163 | UCCGCCAGCUCCACUGCGUcc | -9.6 | 0.764 | 18501 | UUCCCAACUUGUCUACAUUag | -7.2 | 0.938 |
| 19514 | UCCGGCAAACCGAAGAACUtc | -9.6 | 0.538 | 18547 | UUCCCUCCAUUCUUUCGCAgc | -7.1 | 0.727 |
| 19625 | UCCGCCCGGUUCAUGGUCUtg | -9.6 | 0.788 | 19091 | UCUCCGCUGCUUUGCUUGUct | -7.1 | 0.626 |
| 17458 | UCCCCGAUGUUCUGGAUGUgc | -9.2 | 0.444 | 19624 | UCUCCGCCCGGUUCAUGGUct | -7.1 | 0.695 |
| 17776 | UCCCCGAAGGGUACACAUUcg | -9.2 | 0.596 | 17720 | UGGAGAUUUGAAAGGGUAUtt | -7.0 | 0.992 |
| 17884 | UUCCCCAGCAGGAGUUUCAtg | -9.1 | 0.54 | 16915 | UCACGGAGGCGUCCACGUUtg | -6.9 | 0.552 |
| 18202 | UUCCCCUUCAUAUAAGGUAcc | -9.1 | 1.111 | 17129 | UCUGCGGCCUCCUUGUUCAgt | -6.9 | 0.659 |
| 18818 | UGGGGAUAUUGCUCAGAAGtt | -9.1 | 0.93 | 17949 | UCCACGUUGGGGUGGUAGAtc | -6.9 | 0.53 |
| 17137 | UUGGGCUCCAAGAAGAGAUac | -8.9 | 0.771 | 18139 | UCGCAGGUCAUCCAUUUGGtc | -6.9 | 0.953 |
| 17179 | UUGGGCAGGUUCAGCUCGUtt | -8.9 | 0.66 | 18232 | UUGGCUGGUGGCCCUGGAUta | -6.9 | 0.76 |
| 18472 | UUGGGCUUCGUUGGUCUUCca | -8.9 | 0.854 | 18241 | UGGGUAGGUUGAUGAAAGGat | -6.9 | 0.562 |
| 19695 | UGGCCAUGAAGCCUGGGUCgt | -8.9 | 0.826 | 18277 | UGGCUAAUAAUGCUGUCCUta | -6.9 | 0.748 |
| 17768 | UGGCCUCCAGUCCUUGUCCtc | -8.8 | 0.859 | 18703 | UUAGGCGCCGAAGCUCUUGca | -6.9 | 0.841 |
| 17950 | UGGGGUGGUAGAUCUUGGUtg | -8.8 | 0.908 | 18725 | UUGGGUCCACCACUCCAUUag | -6.9 | 0.835 |
| 18282 | UGGCCUGAGAUGCUCAAACag | -8.8 | 0.726 | 19158 | UCAGCGACGCGAUGUCCUCgc | -6.9 | 0.779 |
| 19531 | UGGCCUUCUUUGUUUUAGAgt | -8.8 | 0.957 | 19475 | UCCGUGAUCUCCCCUUGCGaa | -6.9 | 0.973 |
| 19909 | UGCGCGCCAGUUUGGCAAAat | -8.8 | 0.373 | 19634 | UCCGUGGCAUCAUUCUUGAac | -6.9 | 0.687 |
| 17101 | UGGUCCAAAUAUAACUGCAtt | -8.7 | 0.817 | 19682 | UUAGGCGCUGCUUCUUGCCct | -6.9 | 0.913 |
| 17268 | UGUCCCUUUGGUCAAAGUCaa | -8.7 | 0.895 | 19736 | UUGGGUUCUCCUAGAAGGCtc | -6.9 | 0.928 |
| 17587 | UGGGUCUUGAAUGCCAGCCct | -8.7 | 0.758 | 19764 | UCCACGUUGGGGUGAUAGCag | -6.9 | 0.894 |
| 17735 | UGGUCCAUAAAACUUCACUac | -8.7 | 0.955 | 19782 | UAGCCACUGGGGAACUCUAgc | -6.9 | 0.672 |
| 17755 | UGGGUCUUGGAUAUUUGGUtc | -8.7 | 0.839 | 17072 | UUGGACUCCAUCGAUUCUGaa | -6.8 | 0.863 |
| 17998 | UGGACCCAGCCCCAGAGCCat | -8.7 | 0.699 | 17269 | UUGGUCAAAGUCAACGGAGct | -6.8 | 0.986 |
| 18270 | UGGCUCCCUCUCCUUUUGUtg | -8.7 | 0.417 | 17307 | UGAGCAGGCCUGGGAUGCGca | -6.8 | 0.749 |
| 18323 | UGGCUCUGCAGCUGCCAAUag | -8.7 | 0.85 | 17329 | UGUGGAAGGUGACAAAGGCca | -6.8 | 0.725 |
| 18477 | UGCUCCGCUACAUCAUUUGct | -8.7 | 0.642 | 17487 | UUCAGCUUGGAGGCACUCUca | -6.8 | 0.669 |
| 18666 | UGGACCAAGUAUAGUUGAUct | -8.7 | 0.595 | 17683 | UUCUGCUUGUAUUCUUCUGgt | -6.8 | 0.777 |
| 18694 | UGGAGCGGCUGGGUCUCGCtg | -8.7 | 0.692 | 17972 | UUGGUCGGGUAGGAGGAGAgc | -6.8 | 0.799 |
| 19425 | UGACCCAGACCCAGAGAGUgg | -8.7 | 0.618 | 18175 | UGACCAUUGCUAUAAACAUga | -6.8 | 0.962 |
| 16990 | UGCUGCCAUUACUGUUAAUat | -8.5 | 0.576 | 18189 | UUCACCAGUAAACAUGACCtg | -6.8 | 1.032 |
| 17026 | UGUGGCUUGCCAAUGAAACat | -8.5 | 0.949 | 18321 | UGUGGAUCAUCUGGCUCUGca | -6.8 | 0.825 |
| 17056 | UGCUGCCUGGCUAUUGGCUgg | -8.5 | 0.759 | 18450 | UGGUGAUCCCCUUGACUUUgg | -6.8 | 0.662 |
| 17152 | UUGGGGUGAUAGACCAUUGtc | -8.5 | 0.861 | 18457 | UUCUGCACGAUGAAGACGUac | -6.8 | 0.852 |
| 17192 | UGGUGCCGCCCGCCGACUCct | -8.5 | 0.53 | 18460 | UAGCCUAGUCCAUGCUCUAgc | -6.8 | 0.738 |
| 17938 | UGGUGCAAGGCUUCCAGUUct | -8.5 | 0.778 | 18623 | UGGUCAAAUACUGAGUGGCta | -6.8 | 0.909 |
| 18013 | UGCCACCCCUGUACAUCUUtg | -8.5 | 0.623 | 18643 | UGUCCAGACAGAUGACUCCct | -6.8 | 0.756 |
| 18749 | UGUGCCAUCUCCUACUCCUtt | -8.5 | 0.923 | 18681 | UUCAGCUAGCUCCUUCUGAat | -6.8 | 0.874 |
| 19489 | UGCCACUUAGGACUGUUUGgc | -8.5 | 0.812 | 18717 | UUCUGCCAUUUUGCUAGCAct | -6.8 | 0.714 |
| 17255 | UGGAGGUGUGUCUACGCAUgc | -8.3 | 0.768 | 19419 | UAGGGUACAUCACAGAACUgg | -6.8 | 1.164 |
| 17788 | UGGCGAAGAUGGAUAAUCAtc | -8.3 | 0.59 | 19833 | UUGACCACGUCCCCUACCCgc | -6.8 | 0.949 |
| 17843 | UUCGGCCCUGCCGCUGGGCcc | -8.3 | 0.42 | 17142 | UGGAGUUUAUCGUAAGGACtg | -6.7 | 0.767 |
| 18329 | UGCGGAGAGUCAUUGCAGCtg | -8.3 | 0.871 | 17154 | UAGACCAUUGUCUCACACUtc | -6.7 | 0.921 |
| 18389 | UGGAGGGCUGGGUGGCUGAtc | -8.3 | 0.512 | 17670 | UGAGCUGUCCCCGGUACCCtc | -6.7 | 0.719 |
| 18842 | UCUCCCUCAAGCCUAAAUCca | -8.3 | 0.908 | 17894 | UGAGGUCCUCCUCGUUGGGaa | -6.7 | 0.54 |
| 18843 | UCCCUCAAGCCUAAAUCCAga | -8.3 | 0.814 | 17899 | UGGGAAAGACCUUGAUGCCat | -6.7 | 0.573 |
| 19029 | UCUCCCAUACCGGGUUCCGtt | -8.3 | 0.498 | 18043 | UGGACUCCAGUCGGAACCAat | -6.7 | 0.719 |
| 19035 | UCCUCCUUUGGUCUCACACag | -8.3 | 0.98 | 18463 | UGCUCUAGCUGUUUCUAUGgc | -6.7 | 0.953 |
| 19678 | UCCUCCGCAGCUUCUUGCUta | -8.3 | 0.977 | 18485 | UGGAUCAUCUGGAUUGGGAgc | -6.7 | 0.784 |
| 17497 | UGCCUGGUGGAGUUGCUCCag | -8.1 | 0.621 | 18530 | UGCUCUCAUCUGGUUCGGCtt | -6.7 | 0.826 |
| 17498 | UCCAGCACGCUCCGACAUGcc | -8.1 | 0.608 | 18751 | UACUCCUUUCUGGCCUUCUtc | -6.7 | 0.861 |
| 17524 | UCCACCAGCCAUUUGGCAUac | -8.1 | 0.695 | 18765 | UACUCCUUUGCAGUGAAAUtt | -6.7 | 0.95 |
| 17785 | UGGUGGUUCGAAUUUACAUtt | -8.1 | 0.75 | 18397 | UAUGGCUGUAGAGCUUGUUtt | -6.6 | 0.766 |
| 17808 | UCCCACGGAGUCCCUUUCUtt | -8.1 | 0.631 | 18414 | UUGCACUCUGAUUCAGACAag | -6.6 | 0.809 |
| 17966 | UGCGCAGGUUGAAGGCUUUca | -8.1 | 0.698 | 18471 | UAUGGCUUGGGCUUCGUUGgt | -6.6 | 0.94 |
| 18084 | UCUGGCAUUCUUGAGGAAGgc | -8.1 | 0.76 | 18648 | UGACUCCCUGACUGUUGAUgt | -6.6 | 0.705 |
| 18106 | UGGUGGCAAUUUGAGAACAtc | -8.1 | 0.533 | 18718 | UGCCAUUUUGCUAGCACUGat | -6.6 | 0.875 |
| 18421 | UCCUGCCUUCUCAACAAUGaa | -8.1 | 0.614 | 19491 | UGGCAUUAGUGAGAAUUCUgc | -6.6 | 1.069 |
| 18442 | UGGCAGGUAGAGCUGUGAAcc | -8.1 | 0.517 | 19635 | UGGCAUCAUUCUUGAACGCct | -6.6 | 0.934 |
| 18449 | UCCCACACUUUGUCUUCCGac | -8.1 | 0.646 | 17139 | UACUGCAGGCCAUAAAUUAtg | -6.5 | 0.67 |
| 18577 | UCUGGCUUUUCAUAACCAUac | -8.1 | 0.392 | 17219 | UGAUGCCGCUUCACGUUGCgc | -6.5 | 0.724 |
| 18659 | UGGUGGCUUAAAUGGAUAAtc | -8.1 | 0.287 | 17500 | UGCACUUAGAGACGUAGAGag | -6.5 | 0.683 |
| 18823 | UGGGUGUUCUGUAAAGGGUgg | -8.1 | 0.865 | 17578 | UGCAGUUUGGUCACAGGAGtg | -6.5 | 0.805 |
| 19137 | UCAGCCAAUCGCCAUCACUca | -8.1 | 0.858 | 17750 | UUUGGCAGUAAAUCGUGUAgg | -6.5 | 0.929 |
| 19374 | UGCUGGAGCUGAGGCGGUUtt | -8.1 | 0.462 | 17936 | UGGCAAGUCUUGGUGCAAGgc | -6.5 | 0.896 |
| 19502 | UCUGGCACUCCACACCUGGgc | -8.1 | 0.856 | 18316 | UGCUACUACAGCAUCCUGUgg | -6.5 | 0.899 |
| 19530 | UGGUGGCCUUCUUUGUUUUag | -8.1 | 0.457 | 18325 | UAGUGCUUGCAAUGACAAUaa | -6.5 | 0.854 |
| 19893 | UCCCACAGAAGUAAAGGCCgc | -8.1 | 0.802 | 18509 | UUAGGGGCUGCCAUUGGGUat | -6.5 | 0.64 |
| 19920 | UGGCUGUCAGGUUGGGAAGga | -8.1 | 0.663 | 18537 | UGAUGCCAGGAACUGGUUCtg | -6.5 | 0.36 |
| 17906 | UGCGGUCAGUGUCGUCACCtc | -8.0 | 0.887 | 18713 | UGAUGCUAUAUGAAUUCUGcc | -6.5 | 0.9 |
| 18307 | UUCGGGAUUUUGUUUGUACtg | -7.9 | 0.739 | 16831 | UUCCUGGCCAGUCAUCCAGta | -6.4 | 0.757 |
| 16950 | UCCCAGUUGUAUAGAUCGCcc | -7.7 | 0.74 | 16841 | UGUGACUGAUAUGAGCAUUta | -6.4 | 0.76 |
| 17892 | UCAGGGCCCUCGAUGGUGAcc | -7.7 | 0.359 | 17027 | UGGCUUGCCAAUGAAACAUat | -6.4 | 0.907 |
| 19065 | UCGCCAUCACUCAACCAGAat | -7.7 | 0.757 | 17100 | UUCUGGUCCAAAUAUAACUgc | -6.4 | 0.79 |
| 19107 | UCCCAGGUCAUCAGUCCUAgc | -7.7 | 0.704 | 17284 | UUCUGGGGUCCACAGUAUCtg | -6.4 | 0.738 |
| 19711 | UCUGGGUGUUCGAUGAGGUcc | -7.7 | 1.018 | 17483 | UUCCAGGCUCACCCCAGUUct | -6.4 | 0.584 |
| 19728 | UCGGCAGCAUGUGUGUUCAag | -7.7 | 0.865 | 17655 | UAAGCCCUUGCCAAGUCAAta | -6.4 | 0.619 |
| 19948 | UUGGCGUAGAGUCCGGAGCcc | -7.7 | 0.873 | 18051 | UCUCCACAUACCGGAUAAGgg | -6.4 | 0.784 |

## Slide 7
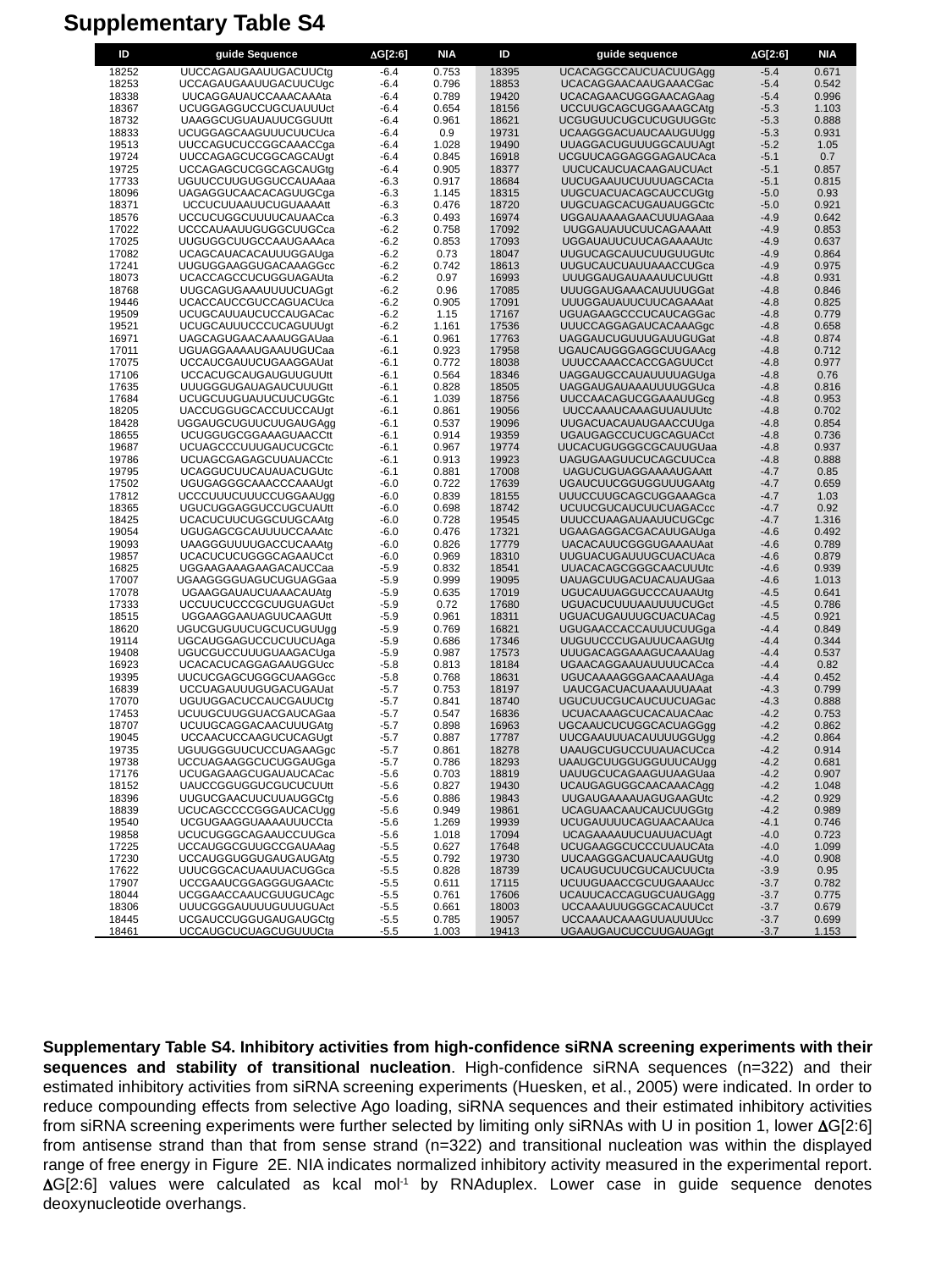

Supplementary Table S4
| ID | guide Sequence | DG[2:6] | NIA | ID | guide sequence | DG[2:6] | NIA |
| --- | --- | --- | --- | --- | --- | --- | --- |
| 18252 | UUCCAGAUGAAUUGACUUCtg | -6.4 | 0.753 | 18395 | UCACAGGCCAUCUACUUGAgg | -5.4 | 0.671 |
| 18253 | UCCAGAUGAAUUGACUUCUgc | -6.4 | 0.796 | 18853 | UCACAGGAACAAUGAAACGac | -5.4 | 0.542 |
| 18338 | UUCAGGAUAUCCAAACAAAta | -6.4 | 0.789 | 19420 | UCACAGAACUGGGAACAGAag | -5.4 | 0.996 |
| 18367 | UCUGGAGGUCCUGCUAUUUct | -6.4 | 0.654 | 18156 | UCCUUGCAGCUGGAAAGCAtg | -5.3 | 1.103 |
| 18732 | UAAGGCUGUAUAUUCGGUUtt | -6.4 | 0.961 | 18621 | UCGUGUUCUGCUCUGUUGGtc | -5.3 | 0.888 |
| 18833 | UCUGGAGCAAGUUUCUUCUca | -6.4 | 0.9 | 19731 | UCAAGGGACUAUCAAUGUUgg | -5.3 | 0.931 |
| 19513 | UUCCAGUCUCCGGCAAACCga | -6.4 | 1.028 | 19490 | UUAGGACUGUUUGGCAUUAgt | -5.2 | 1.05 |
| 19724 | UUCCAGAGCUCGGCAGCAUgt | -6.4 | 0.845 | 16918 | UCGUUCAGGAGGGAGAUCAca | -5.1 | 0.7 |
| 19725 | UCCAGAGCUCGGCAGCAUGtg | -6.4 | 0.905 | 18377 | UUCUCAUCUACAAGAUCUAct | -5.1 | 0.857 |
| 17733 | UGUUCCUUGUGGUCCAUAAaa | -6.3 | 0.917 | 18684 | UUCUGAAUUCUUUUAGCACta | -5.1 | 0.815 |
| 18096 | UAGAGGUCAACACAGUUGCga | -6.3 | 1.145 | 18315 | UUGCUACUACAGCAUCCUGtg | -5.0 | 0.93 |
| 18371 | UCCUCUUAAUUCUGUAAAAtt | -6.3 | 0.476 | 18720 | UUGCUAGCACUGAUAUGGCtc | -5.0 | 0.921 |
| 18576 | UCCUCUGGCUUUUCAUAACca | -6.3 | 0.493 | 16974 | UGGAUAAAAGAACUUUAGAaa | -4.9 | 0.642 |
| 17022 | UCCCAUAAUUGUGGCUUGCca | -6.2 | 0.758 | 17092 | UUGGAUAUUCUUCAGAAAAtt | -4.9 | 0.853 |
| 17025 | UUGUGGCUUGCCAAUGAAAca | -6.2 | 0.853 | 17093 | UGGAUAUUCUUCAGAAAAUtc | -4.9 | 0.637 |
| 17082 | UCAGCAUACACAUUUGGAUga | -6.2 | 0.73 | 18047 | UUGUCAGCAUUCUUGUUGUtc | -4.9 | 0.864 |
| 17241 | UUGUGGAAGGUGACAAAGGcc | -6.2 | 0.742 | 18613 | UUGUCAUCUAUUAAACCUGca | -4.9 | 0.975 |
| 18073 | UCACCAGCCUCUGGUAGAUta | -6.2 | 0.97 | 16993 | UUUGGAUGAUAAAUUCUUGtt | -4.8 | 0.931 |
| 18768 | UUGCAGUGAAAUUUUCUAGgt | -6.2 | 0.96 | 17085 | UUUGGAUGAAACAUUUUGGat | -4.8 | 0.846 |
| 19446 | UCACCAUCCGUCCAGUACUca | -6.2 | 0.905 | 17091 | UUUGGAUAUUCUUCAGAAAat | -4.8 | 0.825 |
| 19509 | UCUGCAUUAUCUCCAUGACac | -6.2 | 1.15 | 17167 | UGUAGAAGCCCUCAUCAGGac | -4.8 | 0.779 |
| 19521 | UCUGCAUUUCCCUCAGUUUgt | -6.2 | 1.161 | 17536 | UUUCCAGGAGAUCACAAAGgc | -4.8 | 0.658 |
| 16971 | UAGCAGUGAACAAAUGGAUaa | -6.1 | 0.961 | 17763 | UAGGAUCUGUUUGAUUGUGat | -4.8 | 0.874 |
| 17011 | UGUAGGAAAAUGAAUUGUCaa | -6.1 | 0.923 | 17958 | UGAUCAUGGGAGGCUUGAAcg | -4.8 | 0.712 |
| 17075 | UCCAUCGAUUCUGAAGGAUat | -6.1 | 0.772 | 18038 | UUUCCAAACCACCGAGUUCct | -4.8 | 0.977 |
| 17106 | UCCACUGCAUGAUGUUGUUtt | -6.1 | 0.564 | 18346 | UAGGAUGCCAUAUUUUAGUga | -4.8 | 0.76 |
| 17635 | UUUGGGUGAUAGAUCUUUGtt | -6.1 | 0.828 | 18505 | UAGGAUGAUAAAUUUUGGUca | -4.8 | 0.816 |
| 17684 | UCUGCUUGUAUUCUUCUGGtc | -6.1 | 1.039 | 18756 | UUCCAACAGUCGGAAAUUGcg | -4.8 | 0.953 |
| 18205 | UACCUGGUGCACCUUCCAUgt | -6.1 | 0.861 | 19056 | UUCCAAAUCAAAGUUAUUUtc | -4.8 | 0.702 |
| 18428 | UGGAUGCUGUUCUUGAUGAgg | -6.1 | 0.537 | 19096 | UUGACUACAUAUGAACCUUga | -4.8 | 0.854 |
| 18655 | UCUGGUGCGGAAAGUAACCtt | -6.1 | 0.914 | 19359 | UGAUGAGCCUCUGCAGUACct | -4.8 | 0.736 |
| 19687 | UCUAGCCCUUUGAUCUCGCtc | -6.1 | 0.967 | 19774 | UUCACUGUGGGCGCAUUGUaa | -4.8 | 0.937 |
| 19786 | UCUAGCGAGAGCUUAUACCtc | -6.1 | 0.913 | 19923 | UAGUGAAGUUCUCAGCUUCca | -4.8 | 0.888 |
| 19795 | UCAGGUCUUCAUAUACUGUtc | -6.1 | 0.881 | 17008 | UAGUCUGUAGGAAAAUGAAtt | -4.7 | 0.85 |
| 17502 | UGUGAGGGCAAACCCAAAUgt | -6.0 | 0.722 | 17639 | UGAUCUUCGGUGGUUUGAAtg | -4.7 | 0.659 |
| 17812 | UCCCUUUCUUUCCUGGAAUgg | -6.0 | 0.839 | 18155 | UUUCCUUGCAGCUGGAAAGca | -4.7 | 1.03 |
| 18365 | UGUCUGGAGGUCCUGCUAUtt | -6.0 | 0.698 | 18742 | UCUUCGUCAUCUUCUAGACcc | -4.7 | 0.92 |
| 18425 | UCACUCUUCUGGCUUGCAAtg | -6.0 | 0.728 | 19545 | UUUCCUAAGAUAAUUCUGCgc | -4.7 | 1.316 |
| 19054 | UGUGAGCGCAUUUUCCAAAtc | -6.0 | 0.476 | 17321 | UGAAGAGGACGACAUUGAUga | -4.6 | 0.492 |
| 19093 | UAAGGGUUUUGACCUCAAAtg | -6.0 | 0.826 | 17779 | UACACAUUCGGGUGAAAUAat | -4.6 | 0.789 |
| 19857 | UCACUCUCUGGGCAGAAUCct | -6.0 | 0.969 | 18310 | UUGUACUGAUUUGCUACUAca | -4.6 | 0.879 |
| 16825 | UGGAAGAAAGAAGACAUCCaa | -5.9 | 0.832 | 18541 | UUACACAGCGGGCAACUUUtc | -4.6 | 0.939 |
| 17007 | UGAAGGGGUAGUCUGUAGGaa | -5.9 | 0.999 | 19095 | UAUAGCUUGACUACAUAUGaa | -4.6 | 1.013 |
| 17078 | UGAAGGAUAUCUAAACAUAtg | -5.9 | 0.635 | 17019 | UGUCAUUAGGUCCCAUAAUtg | -4.5 | 0.641 |
| 17333 | UCCUUCUCCCGCUUGUAGUct | -5.9 | 0.72 | 17680 | UGUACUCUUUAAUUUUCUGct | -4.5 | 0.786 |
| 18515 | UGGAAGGAAUAGUUCAAGUtt | -5.9 | 0.961 | 18311 | UGUACUGAUUUGCUACUACag | -4.5 | 0.921 |
| 18620 | UGUCGUGUUCUGCUCUGUUgg | -5.9 | 0.769 | 16821 | UGUGAACCACCAUUUCUUGga | -4.4 | 0.849 |
| 19114 | UGCAUGGAGUCCUCUUCUAga | -5.9 | 0.686 | 17346 | UUGUUCCCUGAUUUCAAGUtg | -4.4 | 0.344 |
| 19408 | UGUCGUCCUUUGUAAGACUga | -5.9 | 0.987 | 17573 | UUUGACAGGAAAGUCAAAUag | -4.4 | 0.537 |
| 16923 | UCACACUCAGGAGAAUGGUcc | -5.8 | 0.813 | 18184 | UGAACAGGAAUAUUUUCACca | -4.4 | 0.82 |
| 19395 | UUCUCGAGCUGGGCUAAGGcc | -5.8 | 0.768 | 18631 | UGUCAAAAGGGAACAAAUAga | -4.4 | 0.452 |
| 16839 | UCCUAGAUUUGUGACUGAUat | -5.7 | 0.753 | 18197 | UAUCGACUACUAAAUUUAAat | -4.3 | 0.799 |
| 17070 | UGUUGGACUCCAUCGAUUCtg | -5.7 | 0.841 | 18740 | UGUCUUCGUCAUCUUCUAGac | -4.3 | 0.888 |
| 17453 | UCUUGCUUGGUACGAUCAGaa | -5.7 | 0.547 | 16836 | UCUACAAAGCUCACAUACAac | -4.2 | 0.753 |
| 18707 | UCUUGCAGGACAACUUUGAtg | -5.7 | 0.898 | 16963 | UGCAAUCUCUGGCACUAGGgg | -4.2 | 0.862 |
| 19045 | UCCAACUCCAAGUCUCAGUgt | -5.7 | 0.887 | 17787 | UUCGAAUUUACAUUUUGGUgg | -4.2 | 0.864 |
| 19735 | UGUUGGGUUCUCCUAGAAGgc | -5.7 | 0.861 | 18278 | UAAUGCUGUCCUUAUACUCca | -4.2 | 0.914 |
| 19738 | UCCUAGAAGGCUCUGGAUGga | -5.7 | 0.786 | 18293 | UAAUGCUUGGUGGUUUCAUgg | -4.2 | 0.681 |
| 17176 | UCUGAGAAGCUGAUAUCACac | -5.6 | 0.703 | 18819 | UAUUGCUCAGAAGUUAAGUaa | -4.2 | 0.907 |
| 18152 | UAUCCGGUGGUCGUCUCUUtt | -5.6 | 0.827 | 19430 | UCAUGAGUGGCAACAAACAgg | -4.2 | 1.048 |
| 18396 | UUGUCGAACUUCUUAUGGCtg | -5.6 | 0.886 | 19843 | UUGAUGAAAAUAGUGAAGUtc | -4.2 | 0.929 |
| 18839 | UCUCAGCCCCGGGAUCACUgg | -5.6 | 0.949 | 19861 | UCAGUAACAAUCAUCUUGGtg | -4.2 | 0.989 |
| 19540 | UCGUGAAGGUAAAAUUUCCta | -5.6 | 1.269 | 19939 | UCUGAUUUUCAGUAACAAUca | -4.1 | 0.746 |
| 19858 | UCUCUGGGCAGAAUCCUUGca | -5.6 | 1.018 | 17094 | UCAGAAAAUUCUAUUACUAgt | -4.0 | 0.723 |
| 17225 | UCCAUGGCGUUGCCGAUAAag | -5.5 | 0.627 | 17648 | UCUGAAGGCUCCCUUAUCAta | -4.0 | 1.099 |
| 17230 | UCCAUGGUGGUGAUGAUGAtg | -5.5 | 0.792 | 19730 | UUCAAGGGACUAUCAAUGUtg | -4.0 | 0.908 |
| 17622 | UUUCGGCACUAAUUACUGGca | -5.5 | 0.828 | 18739 | UCAUGUCUUCGUCAUCUUCta | -3.9 | 0.95 |
| 17907 | UCCGAAUCGGAGGGUGAACtc | -5.5 | 0.611 | 17115 | UCUUGUAACCGCUUGAAAUcc | -3.7 | 0.782 |
| 18044 | UCGGAACCAAUCGUUGUCAgc | -5.5 | 0.761 | 17606 | UCAUUCACCAGUGCUAUGAgg | -3.7 | 0.775 |
| 18306 | UUUCGGGAUUUUGUUUGUAct | -5.5 | 0.661 | 18003 | UCCAAAUUUGGGCACAUUCct | -3.7 | 0.679 |
| 18445 | UCGAUCCUGGUGAUGAUGCtg | -5.5 | 0.785 | 19057 | UCCAAAUCAAAGUUAUUUUcc | -3.7 | 0.699 |
| 18461 | UCCAUGCUCUAGCUGUUUCta | -5.5 | 1.003 | 19413 | UGAAUGAUCUCCUUGAUAGgt | -3.7 | 1.153 |
Supplementary Table S4. Inhibitory activities from high-confidence siRNA screening experiments with their sequences and stability of transitional nucleation. High-confidence siRNA sequences (n=322) and their estimated inhibitory activities from siRNA screening experiments (Huesken, et al., 2005) were indicated. In order to reduce compounding effects from selective Ago loading, siRNA sequences and their estimated inhibitory activities from siRNA screening experiments were further selected by limiting only siRNAs with U in position 1, lower DG[2:6] from antisense strand than that from sense strand (n=322) and transitional nucleation was within the displayed range of free energy in Figure 2E. NIA indicates normalized inhibitory activity measured in the experimental report. DG[2:6] values were calculated as kcal mol-1 by RNAduplex. Lower case in guide sequence denotes deoxynucleotide overhangs.

## Slide 8
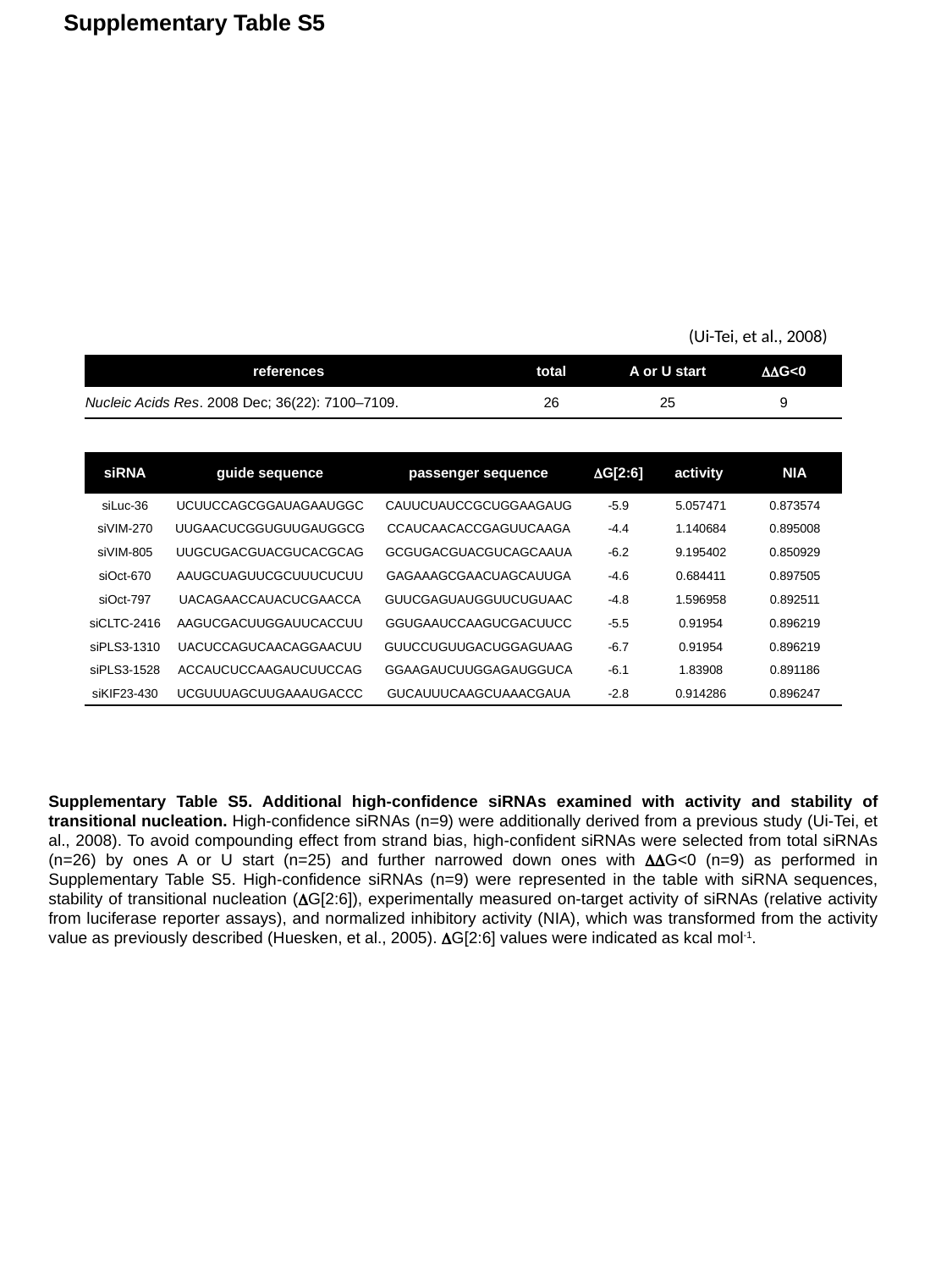

Supplementary Table S5
(Ui-Tei, et al., 2008)
| references | total | A or U start | DDG<0 |
| --- | --- | --- | --- |
| Nucleic Acids Res. 2008 Dec; 36(22): 7100–7109. | 26 | 25 | 9 |
| siRNA | guide sequence | passenger sequence | DG[2:6] | activity | NIA |
| --- | --- | --- | --- | --- | --- |
| siLuc-36 | UCUUCCAGCGGAUAGAAUGGC | CAUUCUAUCCGCUGGAAGAUG | -5.9 | 5.057471 | 0.873574 |
| siVIM-270 | UUGAACUCGGUGUUGAUGGCG | CCAUCAACACCGAGUUCAAGA | -4.4 | 1.140684 | 0.895008 |
| siVIM-805 | UUGCUGACGUACGUCACGCAG | GCGUGACGUACGUCAGCAAUA | -6.2 | 9.195402 | 0.850929 |
| siOct-670 | AAUGCUAGUUCGCUUUCUCUU | GAGAAAGCGAACUAGCAUUGA | -4.6 | 0.684411 | 0.897505 |
| siOct-797 | UACAGAACCAUACUCGAACCA | GUUCGAGUAUGGUUCUGUAAC | -4.8 | 1.596958 | 0.892511 |
| siCLTC-2416 | AAGUCGACUUGGAUUCACCUU | GGUGAAUCCAAGUCGACUUCC | -5.5 | 0.91954 | 0.896219 |
| siPLS3-1310 | UACUCCAGUCAACAGGAACUU | GUUCCUGUUGACUGGAGUAAG | -6.7 | 0.91954 | 0.896219 |
| siPLS3-1528 | ACCAUCUCCAAGAUCUUCCAG | GGAAGAUCUUGGAGAUGGUCA | -6.1 | 1.83908 | 0.891186 |
| siKIF23-430 | UCGUUUAGCUUGAAAUGACCC | GUCAUUUCAAGCUAAACGAUA | -2.8 | 0.914286 | 0.896247 |
Supplementary Table S5. Additional high-confidence siRNAs examined with activity and stability of transitional nucleation. High-confidence siRNAs (n=9) were additionally derived from a previous study (Ui-Tei, et al., 2008). To avoid compounding effect from strand bias, high-confident siRNAs were selected from total siRNAs (n=26) by ones A or U start (n=25) and further narrowed down ones with DDG<0 (n=9) as performed in Supplementary Table S5. High-confidence siRNAs (n=9) were represented in the table with siRNA sequences, stability of transitional nucleation (DG[2:6]), experimentally measured on-target activity of siRNAs (relative activity from luciferase reporter assays), and normalized inhibitory activity (NIA), which was transformed from the activity value as previously described (Huesken, et al., 2005). DG[2:6] values were indicated as kcal mol-1.

## Slide 9
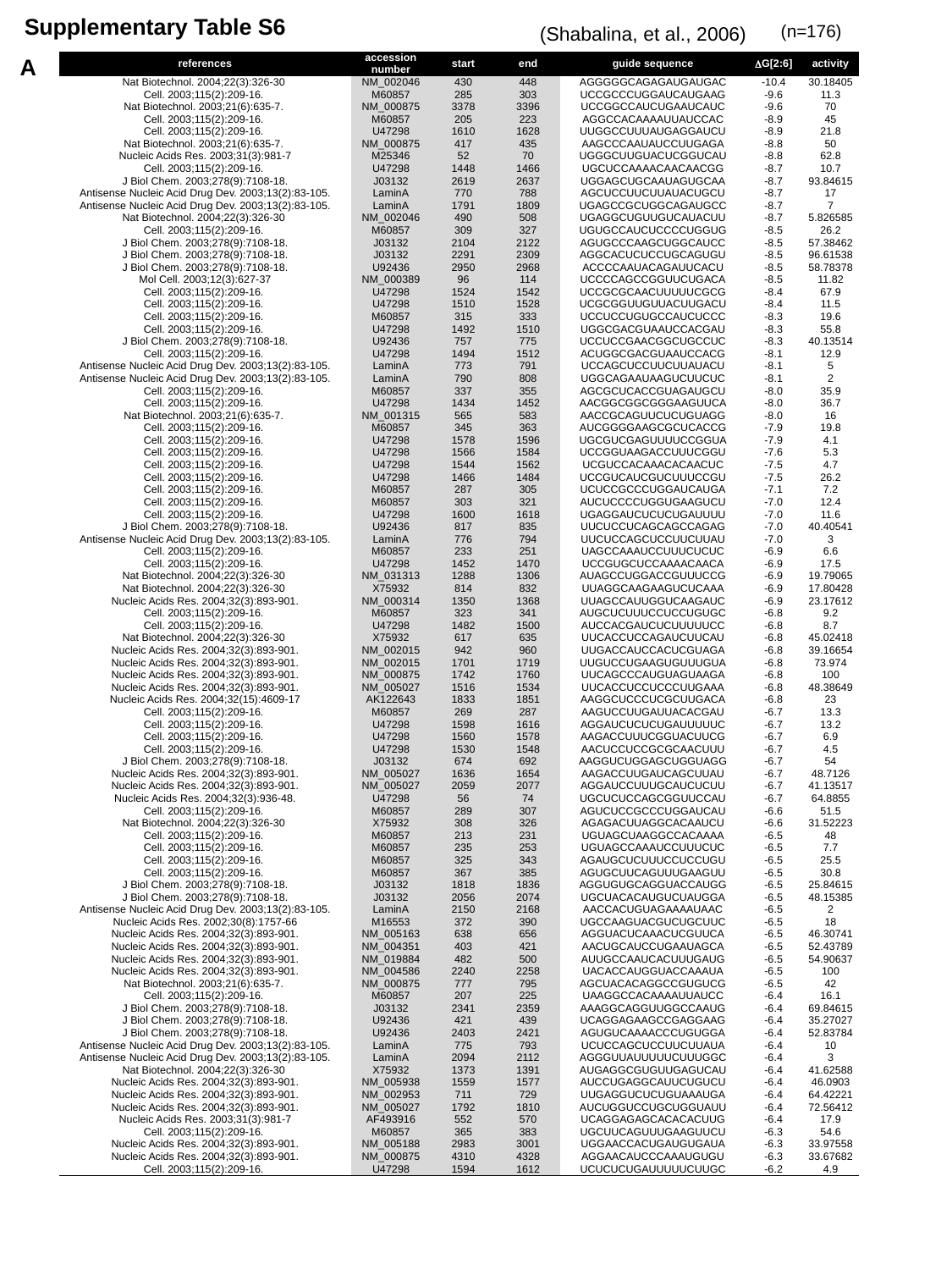

Supplementary Table S6
(n=176)
(Shabalina, et al., 2006)
A
| references | accession number | start | end | guide sequence | DG[2:6] | activity |
| --- | --- | --- | --- | --- | --- | --- |
| Nat Biotechnol. 2004;22(3):326-30 | NM\_002046 | 430 | 448 | AGGGGGCAGAGAUGAUGAC | -10.4 | 30.18405 |
| Cell. 2003;115(2):209-16. | M60857 | 285 | 303 | UCCGCCCUGGAUCAUGAAG | -9.6 | 11.3 |
| Nat Biotechnol. 2003;21(6):635-7. | NM\_000875 | 3378 | 3396 | UCCGGCCAUCUGAAUCAUC | -9.6 | 70 |
| Cell. 2003;115(2):209-16. | M60857 | 205 | 223 | AGGCCACAAAAUUAUCCAC | -8.9 | 45 |
| Cell. 2003;115(2):209-16. | U47298 | 1610 | 1628 | UUGGCCUUUAUGAGGAUCU | -8.9 | 21.8 |
| Nat Biotechnol. 2003;21(6):635-7. | NM\_000875 | 417 | 435 | AAGCCCAAUAUCCUUGAGA | -8.8 | 50 |
| Nucleic Acids Res. 2003;31(3):981-7 | M25346 | 52 | 70 | UGGGCUUGUACUCGGUCAU | -8.8 | 62.8 |
| Cell. 2003;115(2):209-16. | U47298 | 1448 | 1466 | UGCUCCAAAACAACAACGG | -8.7 | 10.7 |
| J Biol Chem. 2003;278(9):7108-18. | J03132 | 2619 | 2637 | UGGAGCUGCAAUAGUGCAA | -8.7 | 93.84615 |
| Antisense Nucleic Acid Drug Dev. 2003;13(2):83-105. | LaminA | 770 | 788 | AGCUCCUUCUUAUACUGCU | -8.7 | 17 |
| Antisense Nucleic Acid Drug Dev. 2003;13(2):83-105. | LaminA | 1791 | 1809 | UGAGCCGCUGGCAGAUGCC | -8.7 | 7 |
| Nat Biotechnol. 2004;22(3):326-30 | NM\_002046 | 490 | 508 | UGAGGCUGUUGUCAUACUU | -8.7 | 5.826585 |
| Cell. 2003;115(2):209-16. | M60857 | 309 | 327 | UGUGCCAUCUCCCCUGGUG | -8.5 | 26.2 |
| J Biol Chem. 2003;278(9):7108-18. | J03132 | 2104 | 2122 | AGUGCCCAAGCUGGCAUCC | -8.5 | 57.38462 |
| J Biol Chem. 2003;278(9):7108-18. | J03132 | 2291 | 2309 | AGGCACUCUCCUGCAGUGU | -8.5 | 96.61538 |
| J Biol Chem. 2003;278(9):7108-18. | U92436 | 2950 | 2968 | ACCCCAAUACAGAUUCACU | -8.5 | 58.78378 |
| Mol Cell. 2003;12(3):627-37 | NM\_000389 | 96 | 114 | UCCCCAGCCGGUUCUGACA | -8.5 | 11.82 |
| Cell. 2003;115(2):209-16. | U47298 | 1524 | 1542 | UCCGCGCAACUUUUUCGCG | -8.4 | 67.9 |
| Cell. 2003;115(2):209-16. | U47298 | 1510 | 1528 | UCGCGGUUGUUACUUGACU | -8.4 | 11.5 |
| Cell. 2003;115(2):209-16. | M60857 | 315 | 333 | UCCUCCUGUGCCAUCUCCC | -8.3 | 19.6 |
| Cell. 2003;115(2):209-16. | U47298 | 1492 | 1510 | UGGCGACGUAAUCCACGAU | -8.3 | 55.8 |
| J Biol Chem. 2003;278(9):7108-18. | U92436 | 757 | 775 | UCCUCCGAACGGCUGCCUC | -8.3 | 40.13514 |
| Cell. 2003;115(2):209-16. | U47298 | 1494 | 1512 | ACUGGCGACGUAAUCCACG | -8.1 | 12.9 |
| Antisense Nucleic Acid Drug Dev. 2003;13(2):83-105. | LaminA | 773 | 791 | UCCAGCUCCUUCUUAUACU | -8.1 | 5 |
| Antisense Nucleic Acid Drug Dev. 2003;13(2):83-105. | LaminA | 790 | 808 | UGGCAGAAUAAGUCUUCUC | -8.1 | 2 |
| Cell. 2003;115(2):209-16. | M60857 | 337 | 355 | AGCGCUCACCGUAGAUGCU | -8.0 | 35.9 |
| Cell. 2003;115(2):209-16. | U47298 | 1434 | 1452 | AACGGCGGCGGGAAGUUCA | -8.0 | 36.7 |
| Nat Biotechnol. 2003;21(6):635-7. | NM\_001315 | 565 | 583 | AACCGCAGUUCUCUGUAGG | -8.0 | 16 |
| Cell. 2003;115(2):209-16. | M60857 | 345 | 363 | AUCGGGGAAGCGCUCACCG | -7.9 | 19.8 |
| Cell. 2003;115(2):209-16. | U47298 | 1578 | 1596 | UGCGUCGAGUUUUCCGGUA | -7.9 | 4.1 |
| Cell. 2003;115(2):209-16. | U47298 | 1566 | 1584 | UCCGGUAAGACCUUUCGGU | -7.6 | 5.3 |
| Cell. 2003;115(2):209-16. | U47298 | 1544 | 1562 | UCGUCCACAAACACAACUC | -7.5 | 4.7 |
| Cell. 2003;115(2):209-16. | U47298 | 1466 | 1484 | UCCGUCAUCGUCUUUCCGU | -7.5 | 26.2 |
| Cell. 2003;115(2):209-16. | M60857 | 287 | 305 | UCUCCGCCCUGGAUCAUGA | -7.1 | 7.2 |
| Cell. 2003;115(2):209-16. | M60857 | 303 | 321 | AUCUCCCCUGGUGAAGUCU | -7.0 | 12.4 |
| Cell. 2003;115(2):209-16. | U47298 | 1600 | 1618 | UGAGGAUCUCUCUGAUUUU | -7.0 | 11.6 |
| J Biol Chem. 2003;278(9):7108-18. | U92436 | 817 | 835 | UUCUCCUCAGCAGCCAGAG | -7.0 | 40.40541 |
| Antisense Nucleic Acid Drug Dev. 2003;13(2):83-105. | LaminA | 776 | 794 | UUCUCCAGCUCCUUCUUAU | -7.0 | 3 |
| Cell. 2003;115(2):209-16. | M60857 | 233 | 251 | UAGCCAAAUCCUUUCUCUC | -6.9 | 6.6 |
| Cell. 2003;115(2):209-16. | U47298 | 1452 | 1470 | UCCGUGCUCCAAAACAACA | -6.9 | 17.5 |
| Nat Biotechnol. 2004;22(3):326-30 | NM\_031313 | 1288 | 1306 | AUAGCCUGGACCGUUUCCG | -6.9 | 19.79065 |
| Nat Biotechnol. 2004;22(3):326-30 | X75932 | 814 | 832 | UUAGGCAAGAAGUCUCAAA | -6.9 | 17.80428 |
| Nucleic Acids Res. 2004;32(3):893-901. | NM\_000314 | 1350 | 1368 | UUAGCCAUUGGUCAAGAUC | -6.9 | 23.17612 |
| Cell. 2003;115(2):209-16. | M60857 | 323 | 341 | AUGCUCUUUCCUCCUGUGC | -6.8 | 9.2 |
| Cell. 2003;115(2):209-16. | U47298 | 1482 | 1500 | AUCCACGAUCUCUUUUUCC | -6.8 | 8.7 |
| Nat Biotechnol. 2004;22(3):326-30 | X75932 | 617 | 635 | UUCACCUCCAGAUCUUCAU | -6.8 | 45.02418 |
| Nucleic Acids Res. 2004;32(3):893-901. | NM\_002015 | 942 | 960 | UUGACCAUCCACUCGUAGA | -6.8 | 39.16654 |
| Nucleic Acids Res. 2004;32(3):893-901. | NM\_002015 | 1701 | 1719 | UUGUCCUGAAGUGUUUGUA | -6.8 | 73.974 |
| Nucleic Acids Res. 2004;32(3):893-901. | NM\_000875 | 1742 | 1760 | UUCAGCCCAUGUAGUAAGA | -6.8 | 100 |
| Nucleic Acids Res. 2004;32(3):893-901. | NM\_005027 | 1516 | 1534 | UUCACCUCCUCCCUUGAAA | -6.8 | 48.38649 |
| Nucleic Acids Res. 2004;32(15):4609-17 | AK122643 | 1833 | 1851 | AAGGCUCCCUCGCUUGACA | -6.8 | 23 |
| Cell. 2003;115(2):209-16. | M60857 | 269 | 287 | AAGUCCUUGAUUACACGAU | -6.7 | 13.3 |
| Cell. 2003;115(2):209-16. | U47298 | 1598 | 1616 | AGGAUCUCUCUGAUUUUUC | -6.7 | 13.2 |
| Cell. 2003;115(2):209-16. | U47298 | 1560 | 1578 | AAGACCUUUCGGUACUUCG | -6.7 | 6.9 |
| Cell. 2003;115(2):209-16. | U47298 | 1530 | 1548 | AACUCCUCCGCGCAACUUU | -6.7 | 4.5 |
| J Biol Chem. 2003;278(9):7108-18. | J03132 | 674 | 692 | AAGGUCUGGAGCUGGUAGG | -6.7 | 54 |
| Nucleic Acids Res. 2004;32(3):893-901. | NM\_005027 | 1636 | 1654 | AAGACCUUGAUCAGCUUAU | -6.7 | 48.7126 |
| Nucleic Acids Res. 2004;32(3):893-901. | NM\_005027 | 2059 | 2077 | AGGAUCCUUUGCAUCUCUU | -6.7 | 41.13517 |
| Nucleic Acids Res. 2004;32(3):936-48. | U47298 | 56 | 74 | UGCUCUCCAGCGGUUCCAU | -6.7 | 64.8855 |
| Cell. 2003;115(2):209-16. | M60857 | 289 | 307 | AGUCUCCGCCCUGGAUCAU | -6.6 | 51.5 |
| Nat Biotechnol. 2004;22(3):326-30 | X75932 | 308 | 326 | AGAGACUUAGGCACAAUCU | -6.6 | 31.52223 |
| Cell. 2003;115(2):209-16. | M60857 | 213 | 231 | UGUAGCUAAGGCCACAAAA | -6.5 | 48 |
| Cell. 2003;115(2):209-16. | M60857 | 235 | 253 | UGUAGCCAAAUCCUUUCUC | -6.5 | 7.7 |
| Cell. 2003;115(2):209-16. | M60857 | 325 | 343 | AGAUGCUCUUUCCUCCUGU | -6.5 | 25.5 |
| Cell. 2003;115(2):209-16. | M60857 | 367 | 385 | AGUGCUUCAGUUUGAAGUU | -6.5 | 30.8 |
| J Biol Chem. 2003;278(9):7108-18. | J03132 | 1818 | 1836 | AGGUGUGCAGGUACCAUGG | -6.5 | 25.84615 |
| J Biol Chem. 2003;278(9):7108-18. | J03132 | 2056 | 2074 | UGCUACACAUGUCUAUGGA | -6.5 | 48.15385 |
| Antisense Nucleic Acid Drug Dev. 2003;13(2):83-105. | LaminA | 2150 | 2168 | AACCACUGUAGAAAAUAAC | -6.5 | 2 |
| Nucleic Acids Res. 2002;30(8):1757-66 | M16553 | 372 | 390 | UGCCAAGUACGUCUGCUUC | -6.5 | 18 |
| Nucleic Acids Res. 2004;32(3):893-901. | NM\_005163 | 638 | 656 | AGGUACUCAAACUCGUUCA | -6.5 | 46.30741 |
| Nucleic Acids Res. 2004;32(3):893-901. | NM\_004351 | 403 | 421 | AACUGCAUCCUGAAUAGCA | -6.5 | 52.43789 |
| Nucleic Acids Res. 2004;32(3):893-901. | NM\_019884 | 482 | 500 | AUUGCCAAUCACUUUGAUG | -6.5 | 54.90637 |
| Nucleic Acids Res. 2004;32(3):893-901. | NM\_004586 | 2240 | 2258 | UACACCAUGGUACCAAAUA | -6.5 | 100 |
| Nat Biotechnol. 2003;21(6):635-7. | NM\_000875 | 777 | 795 | AGCUACACAGGCCGUGUCG | -6.5 | 42 |
| Cell. 2003;115(2):209-16. | M60857 | 207 | 225 | UAAGGCCACAAAAUUAUCC | -6.4 | 16.1 |
| J Biol Chem. 2003;278(9):7108-18. | J03132 | 2341 | 2359 | AAAGGCAGGUUGGCCAAUG | -6.4 | 69.84615 |
| J Biol Chem. 2003;278(9):7108-18. | U92436 | 421 | 439 | UCAGGAGAAGCCGAGGAAG | -6.4 | 35.27027 |
| J Biol Chem. 2003;278(9):7108-18. | U92436 | 2403 | 2421 | AGUGUCAAAACCCUGUGGA | -6.4 | 52.83784 |
| Antisense Nucleic Acid Drug Dev. 2003;13(2):83-105. | LaminA | 775 | 793 | UCUCCAGCUCCUUCUUAUA | -6.4 | 10 |
| Antisense Nucleic Acid Drug Dev. 2003;13(2):83-105. | LaminA | 2094 | 2112 | AGGGUUAUUUUUCUUUGGC | -6.4 | 3 |
| Nat Biotechnol. 2004;22(3):326-30 | X75932 | 1373 | 1391 | AUGAGGCGUGUUGAGUCAU | -6.4 | 41.62588 |
| Nucleic Acids Res. 2004;32(3):893-901. | NM\_005938 | 1559 | 1577 | AUCCUGAGGCAUUCUGUCU | -6.4 | 46.0903 |
| Nucleic Acids Res. 2004;32(3):893-901. | NM\_002953 | 711 | 729 | UUGAGGUCUCUGUAAAUGA | -6.4 | 64.42221 |
| Nucleic Acids Res. 2004;32(3):893-901. | NM\_005027 | 1792 | 1810 | AUCUGGUCCUGCUGGUAUU | -6.4 | 72.56412 |
| Nucleic Acids Res. 2003;31(3):981-7 | AF493916 | 552 | 570 | UCAGGAGAGCACACACUUG | -6.4 | 17.9 |
| Cell. 2003;115(2):209-16. | M60857 | 365 | 383 | UGCUUCAGUUUGAAGUUCU | -6.3 | 54.6 |
| Nucleic Acids Res. 2004;32(3):893-901. | NM\_005188 | 2983 | 3001 | UGGAACCACUGAUGUGAUA | -6.3 | 33.97558 |
| Nucleic Acids Res. 2004;32(3):893-901. | NM\_000875 | 4310 | 4328 | AGGAACAUCCCAAAUGUGU | -6.3 | 33.67682 |
| Cell. 2003;115(2):209-16. | U47298 | 1594 | 1612 | UCUCUCUGAUUUUUCUUGC | -6.2 | 4.9 |

## Slide 10
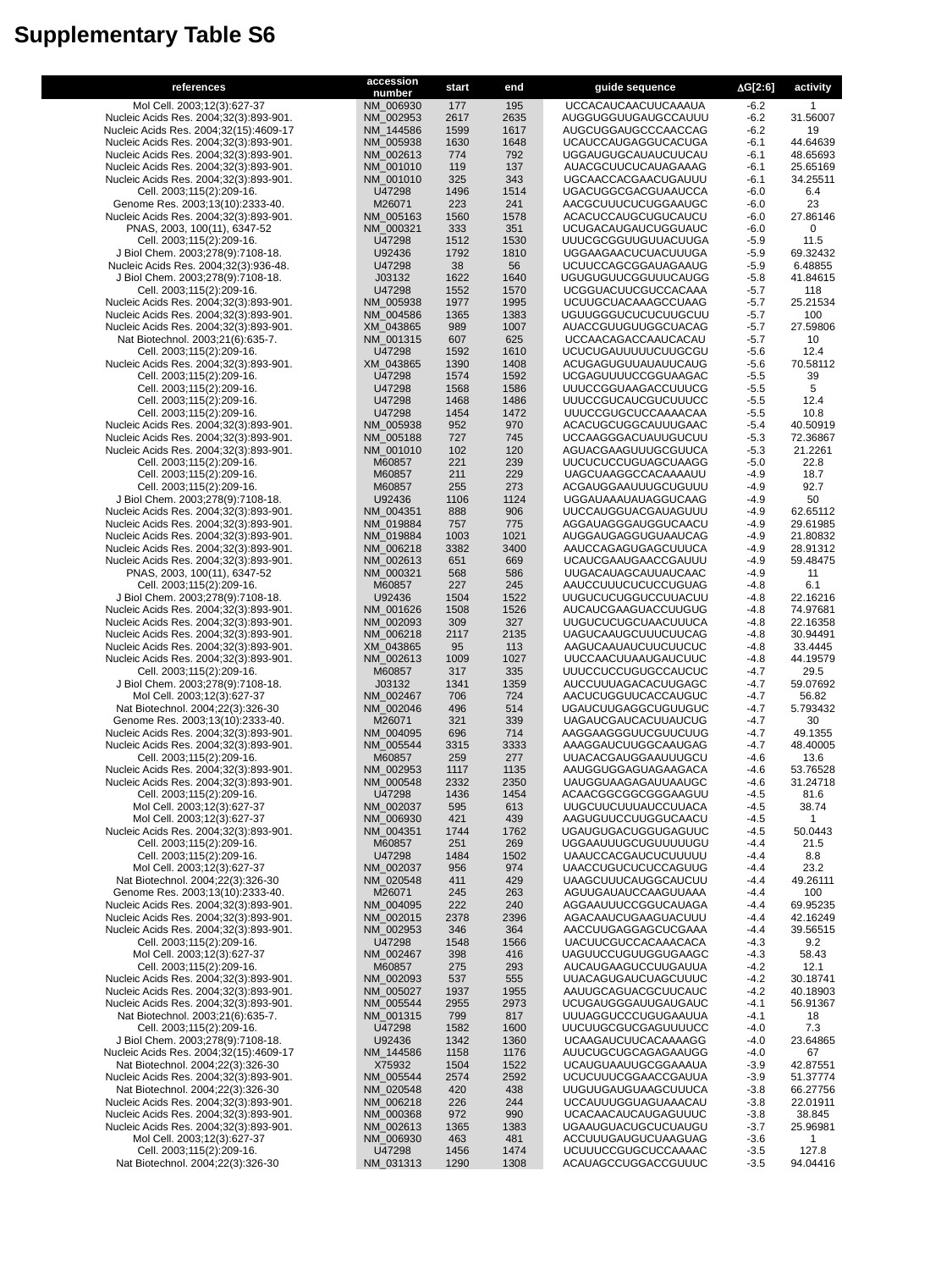

Supplementary Table S6
| references | accession number | start | end | guide sequence | DG[2:6] | activity |
| --- | --- | --- | --- | --- | --- | --- |
| Mol Cell. 2003;12(3):627-37 | NM\_006930 | 177 | 195 | UCCACAUCAACUUCAAAUA | -6.2 | 1 |
| Nucleic Acids Res. 2004;32(3):893-901. | NM\_002953 | 2617 | 2635 | AUGGUGGUUGAUGCCAUUU | -6.2 | 31.56007 |
| Nucleic Acids Res. 2004;32(15):4609-17 | NM\_144586 | 1599 | 1617 | AUGCUGGAUGCCCAACCAG | -6.2 | 19 |
| Nucleic Acids Res. 2004;32(3):893-901. | NM\_005938 | 1630 | 1648 | UCAUCCAUGAGGUCACUGA | -6.1 | 44.64639 |
| Nucleic Acids Res. 2004;32(3):893-901. | NM\_002613 | 774 | 792 | UGGAUGUGCAUAUCUUCAU | -6.1 | 48.65693 |
| Nucleic Acids Res. 2004;32(3):893-901. | NM\_001010 | 119 | 137 | AUACGCUUCUCAUAGAAAG | -6.1 | 25.65169 |
| Nucleic Acids Res. 2004;32(3):893-901. | NM\_001010 | 325 | 343 | UGCAACCACGAACUGAUUU | -6.1 | 34.25511 |
| Cell. 2003;115(2):209-16. | U47298 | 1496 | 1514 | UGACUGGCGACGUAAUCCA | -6.0 | 6.4 |
| Genome Res. 2003;13(10):2333-40. | M26071 | 223 | 241 | AACGCUUUCUCUGGAAUGC | -6.0 | 23 |
| Nucleic Acids Res. 2004;32(3):893-901. | NM\_005163 | 1560 | 1578 | ACACUCCAUGCUGUCAUCU | -6.0 | 27.86146 |
| PNAS, 2003, 100(11), 6347-52 | NM\_000321 | 333 | 351 | UCUGACAUGAUCUGGUAUC | -6.0 | 0 |
| Cell. 2003;115(2):209-16. | U47298 | 1512 | 1530 | UUUCGCGGUUGUUACUUGA | -5.9 | 11.5 |
| J Biol Chem. 2003;278(9):7108-18. | U92436 | 1792 | 1810 | UGGAAGAACUCUACUUUGA | -5.9 | 69.32432 |
| Nucleic Acids Res. 2004;32(3):936-48. | U47298 | 38 | 56 | UCUUCCAGCGGAUAGAAUG | -5.9 | 6.48855 |
| J Biol Chem. 2003;278(9):7108-18. | J03132 | 1622 | 1640 | UGUGUGUUCGGUUUCAUGG | -5.8 | 41.84615 |
| Cell. 2003;115(2):209-16. | U47298 | 1552 | 1570 | UCGGUACUUCGUCCACAAA | -5.7 | 118 |
| Nucleic Acids Res. 2004;32(3):893-901. | NM\_005938 | 1977 | 1995 | UCUUGCUACAAAGCCUAAG | -5.7 | 25.21534 |
| Nucleic Acids Res. 2004;32(3):893-901. | NM\_004586 | 1365 | 1383 | UGUUGGGUCUCUCUUGCUU | -5.7 | 100 |
| Nucleic Acids Res. 2004;32(3):893-901. | XM\_043865 | 989 | 1007 | AUACCGUUGUUGGCUACAG | -5.7 | 27.59806 |
| Nat Biotechnol. 2003;21(6):635-7. | NM\_001315 | 607 | 625 | UCCAACAGACCAAUCACAU | -5.7 | 10 |
| Cell. 2003;115(2):209-16. | U47298 | 1592 | 1610 | UCUCUGAUUUUUCUUGCGU | -5.6 | 12.4 |
| Nucleic Acids Res. 2004;32(3):893-901. | XM\_043865 | 1390 | 1408 | ACUGAGUGUUAUAUUCAUG | -5.6 | 70.58112 |
| Cell. 2003;115(2):209-16. | U47298 | 1574 | 1592 | UCGAGUUUUCCGGUAAGAC | -5.5 | 39 |
| Cell. 2003;115(2):209-16. | U47298 | 1568 | 1586 | UUUCCGGUAAGACCUUUCG | -5.5 | 5 |
| Cell. 2003;115(2):209-16. | U47298 | 1468 | 1486 | UUUCCGUCAUCGUCUUUCC | -5.5 | 12.4 |
| Cell. 2003;115(2):209-16. | U47298 | 1454 | 1472 | UUUCCGUGCUCCAAAACAA | -5.5 | 10.8 |
| Nucleic Acids Res. 2004;32(3):893-901. | NM\_005938 | 952 | 970 | ACACUGCUGGCAUUUGAAC | -5.4 | 40.50919 |
| Nucleic Acids Res. 2004;32(3):893-901. | NM\_005188 | 727 | 745 | UCCAAGGGACUAUUGUCUU | -5.3 | 72.36867 |
| Nucleic Acids Res. 2004;32(3):893-901. | NM\_001010 | 102 | 120 | AGUACGAAGUUUGCGUUCA | -5.3 | 21.2261 |
| Cell. 2003;115(2):209-16. | M60857 | 221 | 239 | UUCUCUCCUGUAGCUAAGG | -5.0 | 22.8 |
| Cell. 2003;115(2):209-16. | M60857 | 211 | 229 | UAGCUAAGGCCACAAAAUU | -4.9 | 18.7 |
| Cell. 2003;115(2):209-16. | M60857 | 255 | 273 | ACGAUGGAAUUUGCUGUUU | -4.9 | 92.7 |
| J Biol Chem. 2003;278(9):7108-18. | U92436 | 1106 | 1124 | UGGAUAAAUAUAGGUCAAG | -4.9 | 50 |
| Nucleic Acids Res. 2004;32(3):893-901. | NM\_004351 | 888 | 906 | UUCCAUGGUACGAUAGUUU | -4.9 | 62.65112 |
| Nucleic Acids Res. 2004;32(3):893-901. | NM\_019884 | 757 | 775 | AGGAUAGGGAUGGUCAACU | -4.9 | 29.61985 |
| Nucleic Acids Res. 2004;32(3):893-901. | NM\_019884 | 1003 | 1021 | AUGGAUGAGGUGUAAUCAG | -4.9 | 21.80832 |
| Nucleic Acids Res. 2004;32(3):893-901. | NM\_006218 | 3382 | 3400 | AAUCCAGAGUGAGCUUUCA | -4.9 | 28.91312 |
| Nucleic Acids Res. 2004;32(3):893-901. | NM\_002613 | 651 | 669 | UCAUCGAAUGAACCGAUUU | -4.9 | 59.48475 |
| PNAS, 2003, 100(11), 6347-52 | NM\_000321 | 568 | 586 | UUGACAUAGCAUUAUCAAC | -4.9 | 11 |
| Cell. 2003;115(2):209-16. | M60857 | 227 | 245 | AAUCCUUUCUCUCCUGUAG | -4.8 | 6.1 |
| J Biol Chem. 2003;278(9):7108-18. | U92436 | 1504 | 1522 | UUGUCUCUGGUCCUUACUU | -4.8 | 22.16216 |
| Nucleic Acids Res. 2004;32(3):893-901. | NM\_001626 | 1508 | 1526 | AUCAUCGAAGUACCUUGUG | -4.8 | 74.97681 |
| Nucleic Acids Res. 2004;32(3):893-901. | NM\_002093 | 309 | 327 | UUGUCUCUGCUAACUUUCA | -4.8 | 22.16358 |
| Nucleic Acids Res. 2004;32(3):893-901. | NM\_006218 | 2117 | 2135 | UAGUCAAUGCUUUCUUCAG | -4.8 | 30.94491 |
| Nucleic Acids Res. 2004;32(3):893-901. | XM\_043865 | 95 | 113 | AAGUCAAUAUCUUCUUCUC | -4.8 | 33.4445 |
| Nucleic Acids Res. 2004;32(3):893-901. | NM\_002613 | 1009 | 1027 | UUCCAACUUAAUGAUCUUC | -4.8 | 44.19579 |
| Cell. 2003;115(2):209-16. | M60857 | 317 | 335 | UUUCCUCCUGUGCCAUCUC | -4.7 | 29.5 |
| J Biol Chem. 2003;278(9):7108-18. | J03132 | 1341 | 1359 | AUCCUUUAGACACUUGAGC | -4.7 | 59.07692 |
| Mol Cell. 2003;12(3):627-37 | NM\_002467 | 706 | 724 | AACUCUGGUUCACCAUGUC | -4.7 | 56.82 |
| Nat Biotechnol. 2004;22(3):326-30 | NM\_002046 | 496 | 514 | UGAUCUUGAGGCUGUUGUC | -4.7 | 5.793432 |
| Genome Res. 2003;13(10):2333-40. | M26071 | 321 | 339 | UAGAUCGAUCACUUAUCUG | -4.7 | 30 |
| Nucleic Acids Res. 2004;32(3):893-901. | NM\_004095 | 696 | 714 | AAGGAAGGGUUCGUUCUUG | -4.7 | 49.1355 |
| Nucleic Acids Res. 2004;32(3):893-901. | NM\_005544 | 3315 | 3333 | AAAGGAUCUUGGCAAUGAG | -4.7 | 48.40005 |
| Cell. 2003;115(2):209-16. | M60857 | 259 | 277 | UUACACGAUGGAAUUUGCU | -4.6 | 13.6 |
| Nucleic Acids Res. 2004;32(3):893-901. | NM\_002953 | 1117 | 1135 | AAUGGUGGAGUAGAAGACA | -4.6 | 53.76528 |
| Nucleic Acids Res. 2004;32(3):893-901. | NM\_000548 | 2332 | 2350 | UAUGGUAAGAGAUUAAUGC | -4.6 | 31.24718 |
| Cell. 2003;115(2):209-16. | U47298 | 1436 | 1454 | ACAACGGCGGCGGGAAGUU | -4.5 | 81.6 |
| Mol Cell. 2003;12(3):627-37 | NM\_002037 | 595 | 613 | UUGCUUCUUUAUCCUUACA | -4.5 | 38.74 |
| Mol Cell. 2003;12(3):627-37 | NM\_006930 | 421 | 439 | AAGUGUUCCUUGGUCAACU | -4.5 | 1 |
| Nucleic Acids Res. 2004;32(3):893-901. | NM\_004351 | 1744 | 1762 | UGAUGUGACUGGUGAGUUC | -4.5 | 50.0443 |
| Cell. 2003;115(2):209-16. | M60857 | 251 | 269 | UGGAAUUUGCUGUUUUUGU | -4.4 | 21.5 |
| Cell. 2003;115(2):209-16. | U47298 | 1484 | 1502 | UAAUCCACGAUCUCUUUUU | -4.4 | 8.8 |
| Mol Cell. 2003;12(3):627-37 | NM\_002037 | 956 | 974 | UAACCUGUCUCUCCAGUUG | -4.4 | 23.2 |
| Nat Biotechnol. 2004;22(3):326-30 | NM\_020548 | 411 | 429 | UAAGCUUUCAUGGCAUCUU | -4.4 | 49.26111 |
| Genome Res. 2003;13(10):2333-40. | M26071 | 245 | 263 | AGUUGAUAUCCAAGUUAAA | -4.4 | 100 |
| Nucleic Acids Res. 2004;32(3):893-901. | NM\_004095 | 222 | 240 | AGGAAUUUCCGGUCAUAGA | -4.4 | 69.95235 |
| Nucleic Acids Res. 2004;32(3):893-901. | NM\_002015 | 2378 | 2396 | AGACAAUCUGAAGUACUUU | -4.4 | 42.16249 |
| Nucleic Acids Res. 2004;32(3):893-901. | NM\_002953 | 346 | 364 | AACCUUGAGGAGCUCGAAA | -4.4 | 39.56515 |
| Cell. 2003;115(2):209-16. | U47298 | 1548 | 1566 | UACUUCGUCCACAAACACA | -4.3 | 9.2 |
| Mol Cell. 2003;12(3):627-37 | NM\_002467 | 398 | 416 | UAGUUCCUGUUGGUGAAGC | -4.3 | 58.43 |
| Cell. 2003;115(2):209-16. | M60857 | 275 | 293 | AUCAUGAAGUCCUUGAUUA | -4.2 | 12.1 |
| Nucleic Acids Res. 2004;32(3):893-901. | NM\_002093 | 537 | 555 | UUACAGUGAUCUAGCUUUC | -4.2 | 30.18741 |
| Nucleic Acids Res. 2004;32(3):893-901. | NM\_005027 | 1937 | 1955 | AAUUGCAGUACGCUUCAUC | -4.2 | 40.18903 |
| Nucleic Acids Res. 2004;32(3):893-901. | NM\_005544 | 2955 | 2973 | UCUGAUGGGAUUGAUGAUC | -4.1 | 56.91367 |
| Nat Biotechnol. 2003;21(6):635-7. | NM\_001315 | 799 | 817 | UUUAGGUCCCUGUGAAUUA | -4.1 | 18 |
| Cell. 2003;115(2):209-16. | U47298 | 1582 | 1600 | UUCUUGCGUCGAGUUUUCC | -4.0 | 7.3 |
| J Biol Chem. 2003;278(9):7108-18. | U92436 | 1342 | 1360 | UCAAGAUCUUCACAAAAGG | -4.0 | 23.64865 |
| Nucleic Acids Res. 2004;32(15):4609-17 | NM\_144586 | 1158 | 1176 | AUUCUGCUGCAGAGAAUGG | -4.0 | 67 |
| Nat Biotechnol. 2004;22(3):326-30 | X75932 | 1504 | 1522 | UCAUGUAAUUGCGGAAAUA | -3.9 | 42.87551 |
| Nucleic Acids Res. 2004;32(3):893-901. | NM\_005544 | 2574 | 2592 | UCUCUUUCGGAACCGAUUA | -3.9 | 51.37774 |
| Nat Biotechnol. 2004;22(3):326-30 | NM\_020548 | 420 | 438 | UUGUUGAUGUAAGCUUUCA | -3.8 | 66.27756 |
| Nucleic Acids Res. 2004;32(3):893-901. | NM\_006218 | 226 | 244 | UCCAUUUGGUAGUAAACAU | -3.8 | 22.01911 |
| Nucleic Acids Res. 2004;32(3):893-901. | NM\_000368 | 972 | 990 | UCACAACAUCAUGAGUUUC | -3.8 | 38.845 |
| Nucleic Acids Res. 2004;32(3):893-901. | NM\_002613 | 1365 | 1383 | UGAAUGUACUGCUCUAUGU | -3.7 | 25.96981 |
| Mol Cell. 2003;12(3):627-37 | NM\_006930 | 463 | 481 | ACCUUUGAUGUCUAAGUAG | -3.6 | 1 |
| Cell. 2003;115(2):209-16. | U47298 | 1456 | 1474 | UCUUUCCGUGCUCCAAAAC | -3.5 | 127.8 |
| Nat Biotechnol. 2004;22(3):326-30 | NM\_031313 | 1290 | 1308 | ACAUAGCCUGGACCGUUUC | -3.5 | 94.04416 |

## Slide 11
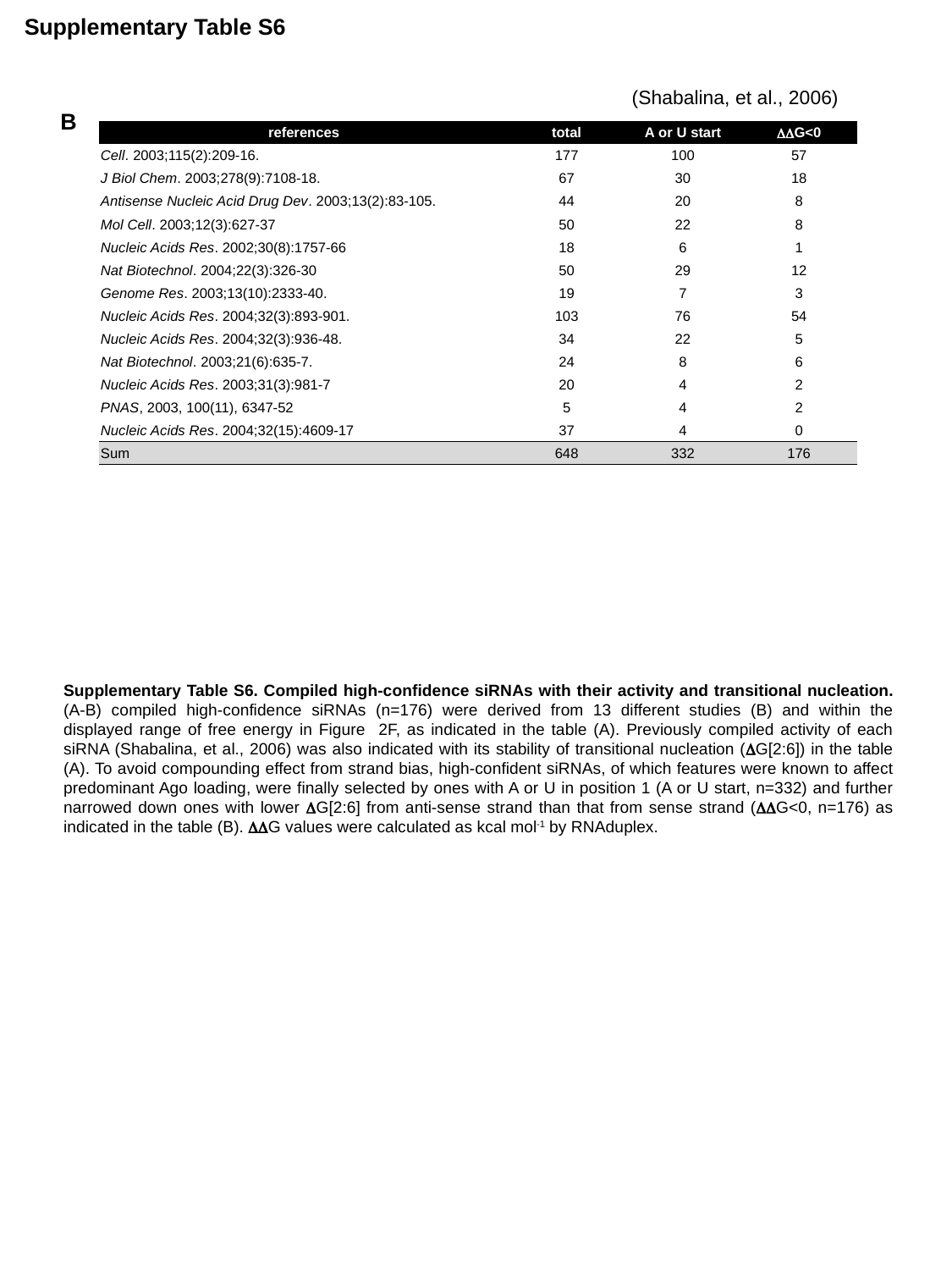

Supplementary Table S6
(Shabalina, et al., 2006)
B
| references | total | A or U start | DDG<0 |
| --- | --- | --- | --- |
| Cell. 2003;115(2):209-16. | 177 | 100 | 57 |
| J Biol Chem. 2003;278(9):7108-18. | 67 | 30 | 18 |
| Antisense Nucleic Acid Drug Dev. 2003;13(2):83-105. | 44 | 20 | 8 |
| Mol Cell. 2003;12(3):627-37 | 50 | 22 | 8 |
| Nucleic Acids Res. 2002;30(8):1757-66 | 18 | 6 | 1 |
| Nat Biotechnol. 2004;22(3):326-30 | 50 | 29 | 12 |
| Genome Res. 2003;13(10):2333-40. | 19 | 7 | 3 |
| Nucleic Acids Res. 2004;32(3):893-901. | 103 | 76 | 54 |
| Nucleic Acids Res. 2004;32(3):936-48. | 34 | 22 | 5 |
| Nat Biotechnol. 2003;21(6):635-7. | 24 | 8 | 6 |
| Nucleic Acids Res. 2003;31(3):981-7 | 20 | 4 | 2 |
| PNAS, 2003, 100(11), 6347-52 | 5 | 4 | 2 |
| Nucleic Acids Res. 2004;32(15):4609-17 | 37 | 4 | 0 |
| Sum | 648 | 332 | 176 |
Supplementary Table S6. Compiled high-confidence siRNAs with their activity and transitional nucleation. (A-B) compiled high-confidence siRNAs (n=176) were derived from 13 different studies (B) and within the displayed range of free energy in Figure 2F, as indicated in the table (A). Previously compiled activity of each siRNA (Shabalina, et al., 2006) was also indicated with its stability of transitional nucleation (DG[2:6]) in the table (A). To avoid compounding effect from strand bias, high-confident siRNAs, of which features were known to affect predominant Ago loading, were finally selected by ones with A or U in position 1 (A or U start, n=332) and further narrowed down ones with lower DG[2:6] from anti-sense strand than that from sense strand (DDG<0, n=176) as indicated in the table (B). DDG values were calculated as kcal mol-1 by RNAduplex.

## Slide 12
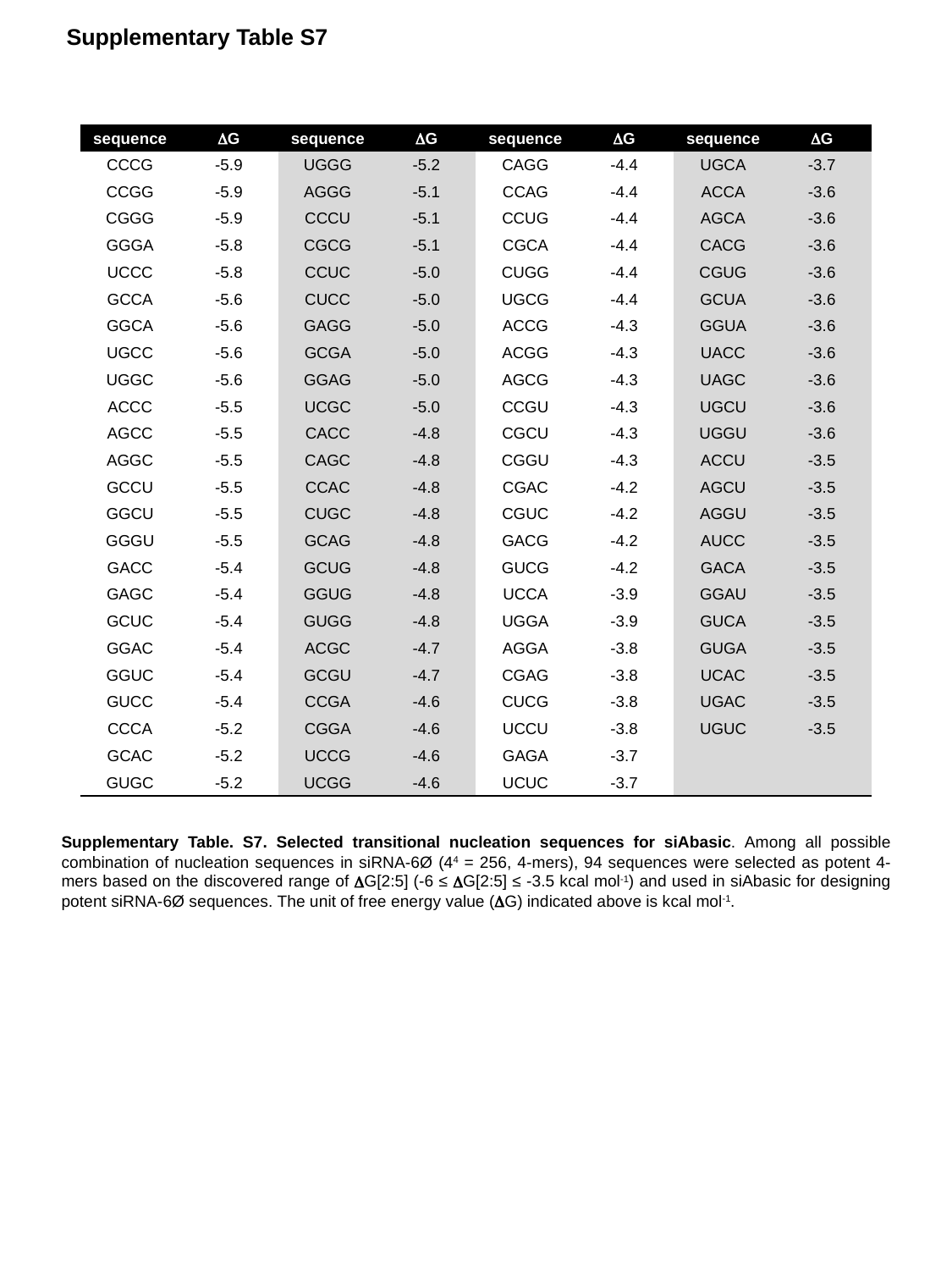

Supplementary Table S7
| sequence | DG | sequence | DG | sequence | DG | sequence | DG |
| --- | --- | --- | --- | --- | --- | --- | --- |
| CCCG | -5.9 | UGGG | -5.2 | CAGG | -4.4 | UGCA | -3.7 |
| CCGG | -5.9 | AGGG | -5.1 | CCAG | -4.4 | ACCA | -3.6 |
| CGGG | -5.9 | CCCU | -5.1 | CCUG | -4.4 | AGCA | -3.6 |
| GGGA | -5.8 | CGCG | -5.1 | CGCA | -4.4 | CACG | -3.6 |
| UCCC | -5.8 | CCUC | -5.0 | CUGG | -4.4 | CGUG | -3.6 |
| GCCA | -5.6 | CUCC | -5.0 | UGCG | -4.4 | GCUA | -3.6 |
| GGCA | -5.6 | GAGG | -5.0 | ACCG | -4.3 | GGUA | -3.6 |
| UGCC | -5.6 | GCGA | -5.0 | ACGG | -4.3 | UACC | -3.6 |
| UGGC | -5.6 | GGAG | -5.0 | AGCG | -4.3 | UAGC | -3.6 |
| ACCC | -5.5 | UCGC | -5.0 | CCGU | -4.3 | UGCU | -3.6 |
| AGCC | -5.5 | CACC | -4.8 | CGCU | -4.3 | UGGU | -3.6 |
| AGGC | -5.5 | CAGC | -4.8 | CGGU | -4.3 | ACCU | -3.5 |
| GCCU | -5.5 | CCAC | -4.8 | CGAC | -4.2 | AGCU | -3.5 |
| GGCU | -5.5 | CUGC | -4.8 | CGUC | -4.2 | AGGU | -3.5 |
| GGGU | -5.5 | GCAG | -4.8 | GACG | -4.2 | AUCC | -3.5 |
| GACC | -5.4 | GCUG | -4.8 | GUCG | -4.2 | GACA | -3.5 |
| GAGC | -5.4 | GGUG | -4.8 | UCCA | -3.9 | GGAU | -3.5 |
| GCUC | -5.4 | GUGG | -4.8 | UGGA | -3.9 | GUCA | -3.5 |
| GGAC | -5.4 | ACGC | -4.7 | AGGA | -3.8 | GUGA | -3.5 |
| GGUC | -5.4 | GCGU | -4.7 | CGAG | -3.8 | UCAC | -3.5 |
| GUCC | -5.4 | CCGA | -4.6 | CUCG | -3.8 | UGAC | -3.5 |
| CCCA | -5.2 | CGGA | -4.6 | UCCU | -3.8 | UGUC | -3.5 |
| GCAC | -5.2 | UCCG | -4.6 | GAGA | -3.7 | | |
| GUGC | -5.2 | UCGG | -4.6 | UCUC | -3.7 | | |
Supplementary Table. S7. Selected transitional nucleation sequences for siAbasic. Among all possible combination of nucleation sequences in siRNA-6Ø (44 = 256, 4-mers), 94 sequences were selected as potent 4-mers based on the discovered range of DG[2:5] (-6 ≤ DG[2:5] ≤ -3.5 kcal mol-1) and used in siAbasic for designing potent siRNA-6Ø sequences. The unit of free energy value (DG) indicated above is kcal mol-1.

## Slide 13
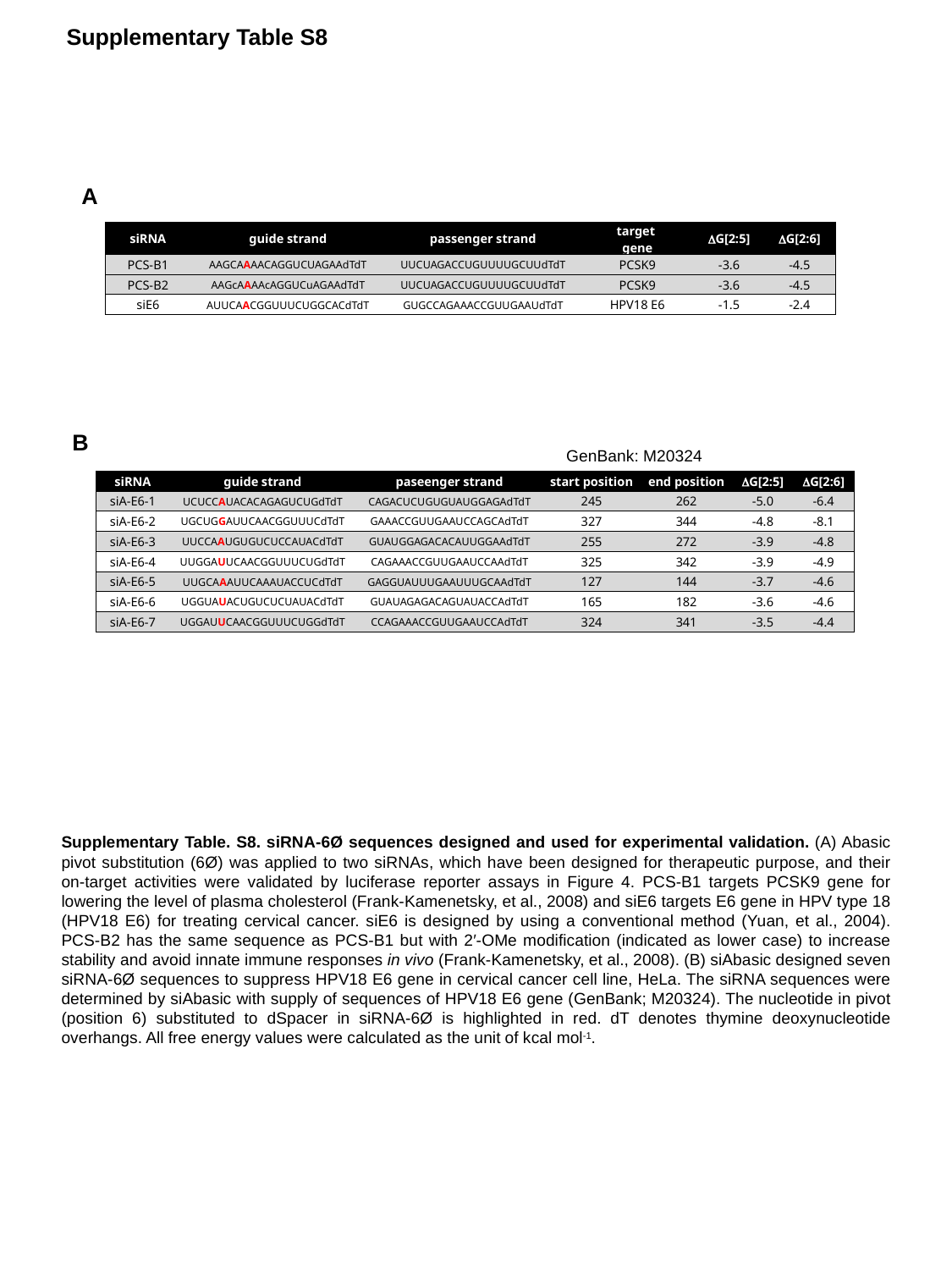

Supplementary Table S8
A
| siRNA | guide strand | passenger strand | target gene | DG[2:5] | DG[2:6] |
| --- | --- | --- | --- | --- | --- |
| PCS-B1 | AAGCAAAACAGGUCUAGAAdTdT | UUCUAGACCUGUUUUGCUUdTdT | PCSK9 | -3.6 | -4.5 |
| PCS-B2 | AAGcAAAAcAGGUCuAGAAdTdT | UUCUAGACCUGUUUUGCUUdTdT | PCSK9 | -3.6 | -4.5 |
| siE6 | AUUCAACGGUUUCUGGCACdTdT | GUGCCAGAAACCGUUGAAUdTdT | HPV18 E6 | -1.5 | -2.4 |
B
GenBank: M20324
| siRNA | guide strand | paseenger strand | start position | end position | DG[2:5] | DG[2:6] |
| --- | --- | --- | --- | --- | --- | --- |
| siA-E6-1 | UCUCCAUACACAGAGUCUGdTdT | CAGACUCUGUGUAUGGAGAdTdT | 245 | 262 | -5.0 | -6.4 |
| siA-E6-2 | UGCUGGAUUCAACGGUUUCdTdT | GAAACCGUUGAAUCCAGCAdTdT | 327 | 344 | -4.8 | -8.1 |
| siA-E6-3 | UUCCAAUGUGUCUCCAUACdTdT | GUAUGGAGACACAUUGGAAdTdT | 255 | 272 | -3.9 | -4.8 |
| siA-E6-4 | UUGGAUUCAACGGUUUCUGdTdT | CAGAAACCGUUGAAUCCAAdTdT | 325 | 342 | -3.9 | -4.9 |
| siA-E6-5 | UUGCAAAUUCAAAUACCUCdTdT | GAGGUAUUUGAAUUUGCAAdTdT | 127 | 144 | -3.7 | -4.6 |
| siA-E6-6 | UGGUAUACUGUCUCUAUACdTdT | GUAUAGAGACAGUAUACCAdTdT | 165 | 182 | -3.6 | -4.6 |
| siA-E6-7 | UGGAUUCAACGGUUUCUGGdTdT | CCAGAAACCGUUGAAUCCAdTdT | 324 | 341 | -3.5 | -4.4 |
Supplementary Table. S8. siRNA-6Ø sequences designed and used for experimental validation. (A) Abasic pivot substitution (6Ø) was applied to two siRNAs, which have been designed for therapeutic purpose, and their on-target activities were validated by luciferase reporter assays in Figure 4. PCS-B1 targets PCSK9 gene for lowering the level of plasma cholesterol (Frank-Kamenetsky, et al., 2008) and siE6 targets E6 gene in HPV type 18 (HPV18 E6) for treating cervical cancer. siE6 is designed by using a conventional method (Yuan, et al., 2004). PCS-B2 has the same sequence as PCS-B1 but with 2′-OMe modification (indicated as lower case) to increase stability and avoid innate immune responses in vivo (Frank-Kamenetsky, et al., 2008). (B) siAbasic designed seven siRNA-6Ø sequences to suppress HPV18 E6 gene in cervical cancer cell line, HeLa. The siRNA sequences were determined by siAbasic with supply of sequences of HPV18 E6 gene (GenBank; M20324). The nucleotide in pivot (position 6) substituted to dSpacer in siRNA-6Ø is highlighted in red. dT denotes thymine deoxynucleotide overhangs. All free energy values were calculated as the unit of kcal mol-1.

## Slide 14
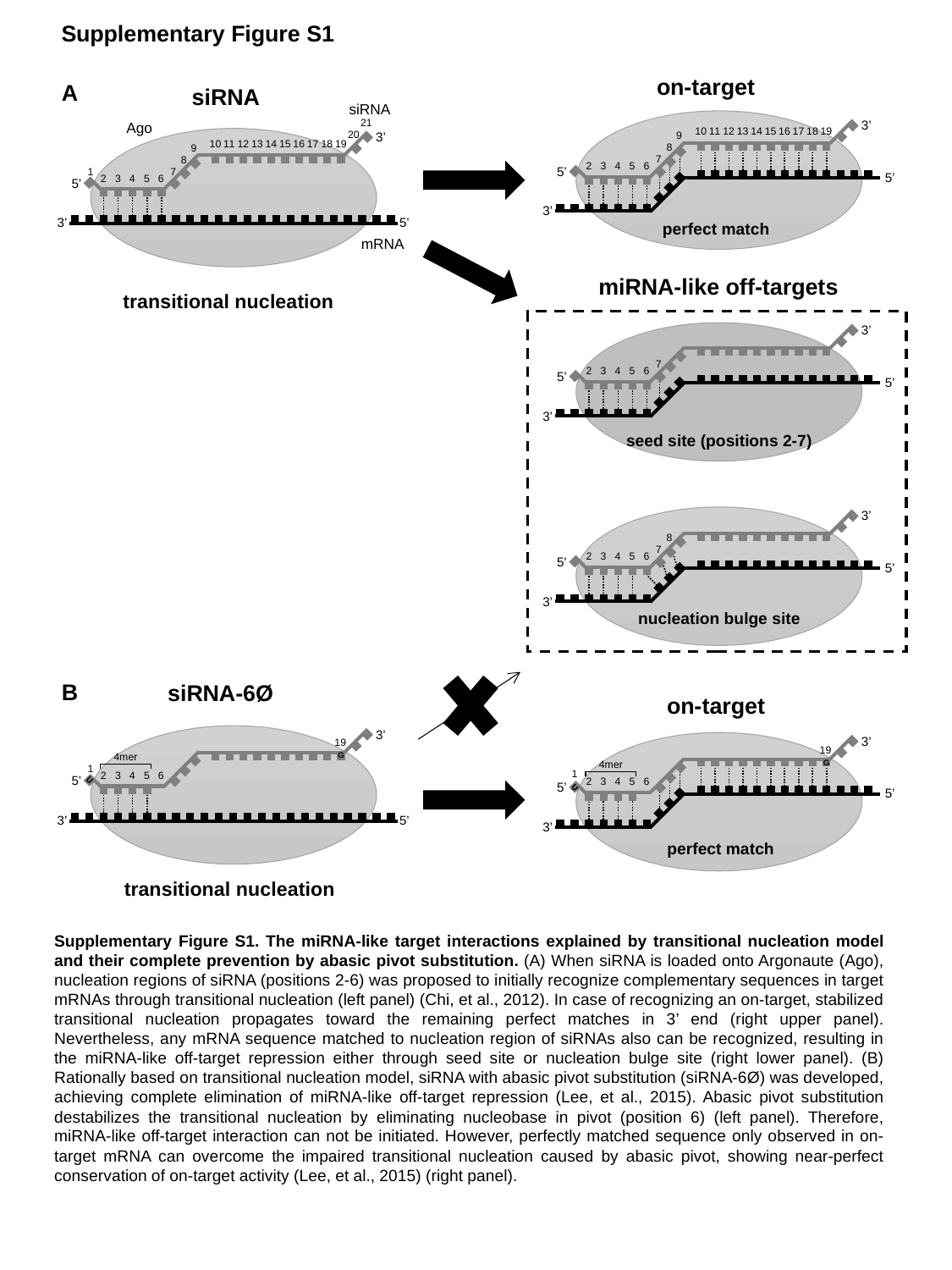

Supplementary Figure S1
on-target
A
siRNA
siRNA
Ago
perfect match
mRNA
miRNA-like off-targets
transitional nucleation
seed site (positions 2-7)
nucleation bulge site
B
siRNA-6Ø
on-target
perfect match
transitional nucleation
Supplementary Figure S1. The miRNA-like target interactions explained by transitional nucleation model and their complete prevention by abasic pivot substitution. (A) When siRNA is loaded onto Argonaute (Ago), nucleation regions of siRNA (positions 2-6) was proposed to initially recognize complementary sequences in target mRNAs through transitional nucleation (left panel) (Chi, et al., 2012). In case of recognizing an on-target, stabilized transitional nucleation propagates toward the remaining perfect matches in 3’ end (right upper panel). Nevertheless, any mRNA sequence matched to nucleation region of siRNAs also can be recognized, resulting in the miRNA-like off-target repression either through seed site or nucleation bulge site (right lower panel). (B) Rationally based on transitional nucleation model, siRNA with abasic pivot substitution (siRNA-6Ø) was developed, achieving complete elimination of miRNA-like off-target repression (Lee, et al., 2015). Abasic pivot substitution destabilizes the transitional nucleation by eliminating nucleobase in pivot (position 6) (left panel). Therefore, miRNA-like off-target interaction can not be initiated. However, perfectly matched sequence only observed in on-target mRNA can overcome the impaired transitional nucleation caused by abasic pivot, showing near-perfect conservation of on-target activity (Lee, et al., 2015) (right panel).

## Slide 15
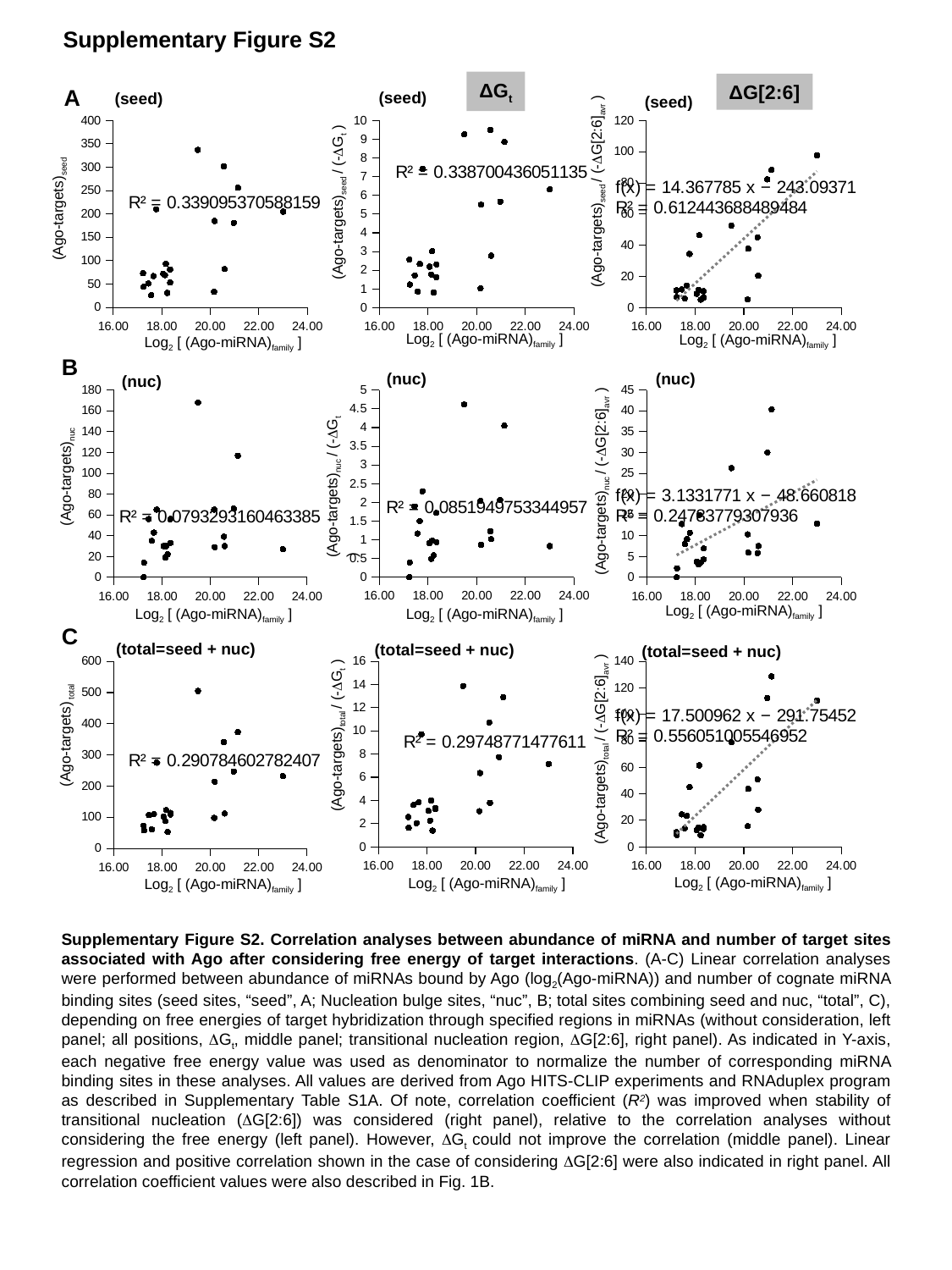

Supplementary Figure S2
ΔGt
ΔG[2:6]
A
(Ago-targets)seed / (-DG[2:6]avr )
(seed)
(seed)
(seed)
(Ago-targets)seed / (-DGt )
### Chart
| Category | |
|---|---|
### Chart
| Category | |
|---|---|
### Chart
| Category | |
|---|---|(Ago-targets)seed
Log2 [ (Ago-miRNA)family ]
Log2 [ (Ago-miRNA)family ]
Log2 [ (Ago-miRNA)family ]
B
(nuc)
(nuc)
(nuc)
(Ago-targets)nuc / (-DG[2:6]avr )
### Chart
| Category | |
|---|---|
### Chart
| Category | |
|---|---|
### Chart
| Category | |
|---|---|(Ago-targets)nuc / (-DGt )
(Ago-targets)nuc
Log2 [ (Ago-miRNA)family ]
Log2 [ (Ago-miRNA)family ]
Log2 [ (Ago-miRNA)family ]
C
(Ago-targets)total / (-DGt )
(total=seed + nuc)
(total=seed + nuc)
(Ago-targets)total / (-DG[2:6]avr )
(total=seed + nuc)
### Chart
| Category | |
|---|---|
### Chart
| Category | |
|---|---|
### Chart
| Category | |
|---|---|(Ago-targets) total
Log2 [ (Ago-miRNA)family ]
Log2 [ (Ago-miRNA)family ]
Log2 [ (Ago-miRNA)family ]
Supplementary Figure S2. Correlation analyses between abundance of miRNA and number of target sites associated with Ago after considering free energy of target interactions. (A-C) Linear correlation analyses were performed between abundance of miRNAs bound by Ago (log2(Ago-miRNA)) and number of cognate miRNA binding sites (seed sites, “seed”, A; Nucleation bulge sites, “nuc”, B; total sites combining seed and nuc, “total”, C), depending on free energies of target hybridization through specified regions in miRNAs (without consideration, left panel; all positions, DGt, middle panel; transitional nucleation region, DG[2:6], right panel). As indicated in Y-axis, each negative free energy value was used as denominator to normalize the number of corresponding miRNA binding sites in these analyses. All values are derived from Ago HITS-CLIP experiments and RNAduplex program as described in Supplementary Table S1A. Of note, correlation coefficient (R2) was improved when stability of transitional nucleation (DG[2:6]) was considered (right panel), relative to the correlation analyses without considering the free energy (left panel). However, DGt could not improve the correlation (middle panel). Linear regression and positive correlation shown in the case of considering DG[2:6] were also indicated in right panel. All correlation coefficient values were also described in Fig. 1B.

## Slide 16
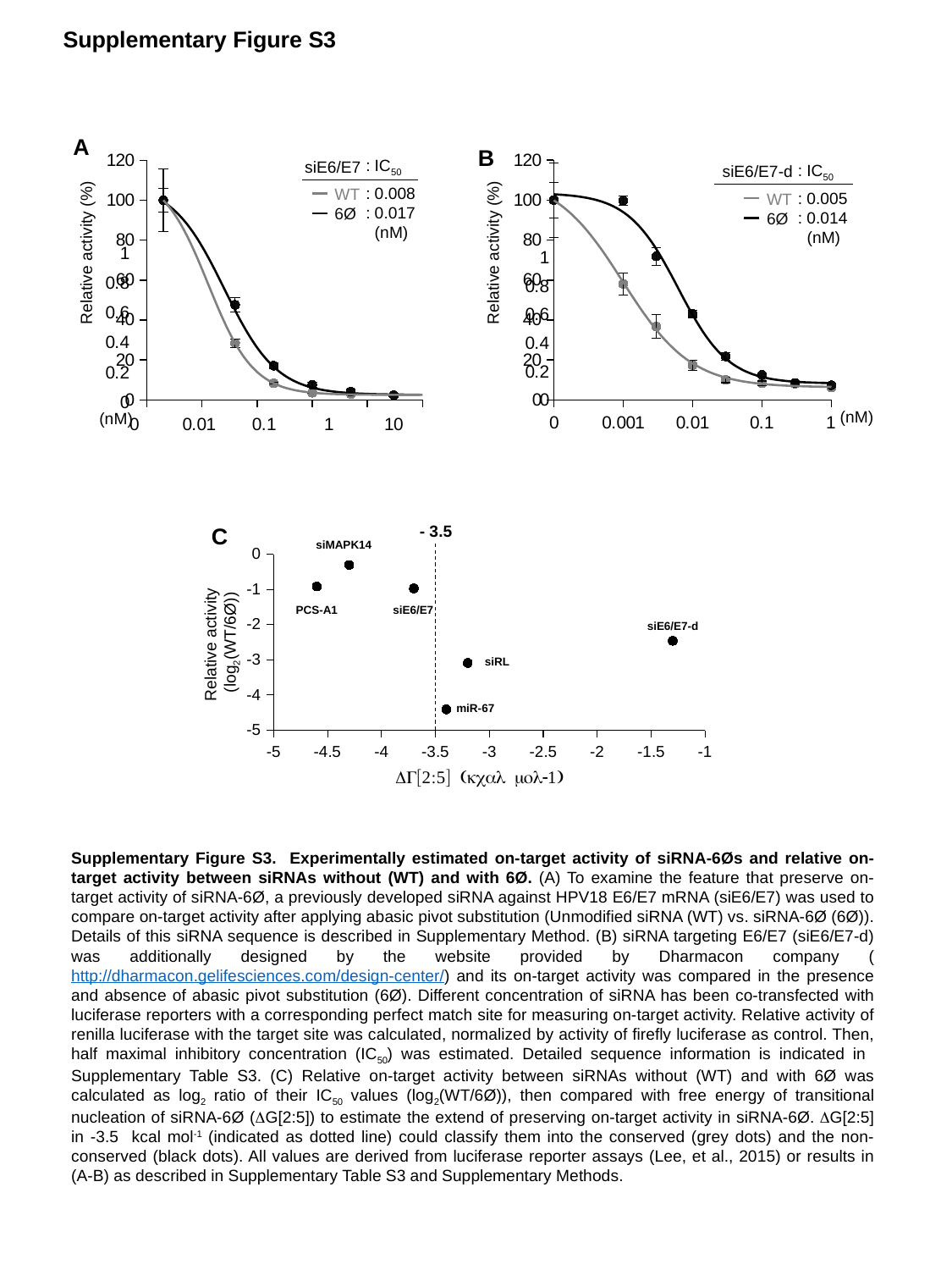

Supplementary Figure S3
A
B
### Chart
| Category | | WT | 6pi | |
|---|---|---|---|---|siE6/E7
: IC50
: 0.008
WT
: 0.017
6Ø
: (nM)
### Chart
| Category | | WT | 6pi | |
|---|---|---|---|---|siE6/E7-d
: IC50
: 0.005
WT
: 0.014
6Ø
: (nM)
Relative activity (%)
Relative activity (%)
### Chart
| Category | |
|---|---|
| 0 | 0.0 |
| 1.0000000000000004E-2 | 0.0 |
| 0.1 | 0.0 |
| 1 | 0.0 |
| 10 | None |
### Chart
| Category | |
|---|---|
| 0 | 0.0 |
| 1.0000000000000005E-3 | 0.0 |
| 1.0000000000000004E-2 | 0.0 |
| 0.1 | 0.0 |
| 1 | 0.0 |(nM)
(nM)
- 3.5
siMAPK14
### Chart
| Category | |
|---|---|PCS-A1
siE6/E7
siE6/E7-d
siRL
miR-67
C
Relative activity
(log2(WT/6Ø))
Supplementary Figure S3. Experimentally estimated on-target activity of siRNA-6Øs and relative on-target activity between siRNAs without (WT) and with 6Ø. (A) To examine the feature that preserve on-target activity of siRNA-6Ø, a previously developed siRNA against HPV18 E6/E7 mRNA (siE6/E7) was used to compare on-target activity after applying abasic pivot substitution (Unmodified siRNA (WT) vs. siRNA-6Ø (6Ø)). Details of this siRNA sequence is described in Supplementary Method. (B) siRNA targeting E6/E7 (siE6/E7-d) was additionally designed by the website provided by Dharmacon company (http://dharmacon.gelifesciences.com/design-center/) and its on-target activity was compared in the presence and absence of abasic pivot substitution (6Ø). Different concentration of siRNA has been co-transfected with luciferase reporters with a corresponding perfect match site for measuring on-target activity. Relative activity of renilla luciferase with the target site was calculated, normalized by activity of firefly luciferase as control. Then, half maximal inhibitory concentration (IC50) was estimated. Detailed sequence information is indicated in Supplementary Table S3. (C) Relative on-target activity between siRNAs without (WT) and with 6Ø was calculated as log2 ratio of their IC50 values (log2(WT/6Ø)), then compared with free energy of transitional nucleation of siRNA-6Ø (DG[2:5]) to estimate the extend of preserving on-target activity in siRNA-6Ø. DG[2:5] in -3.5 kcal mol-1 (indicated as dotted line) could classify them into the conserved (grey dots) and the non-conserved (black dots). All values are derived from luciferase reporter assays (Lee, et al., 2015) or results in (A-B) as described in Supplementary Table S3 and Supplementary Methods.

## Slide 17
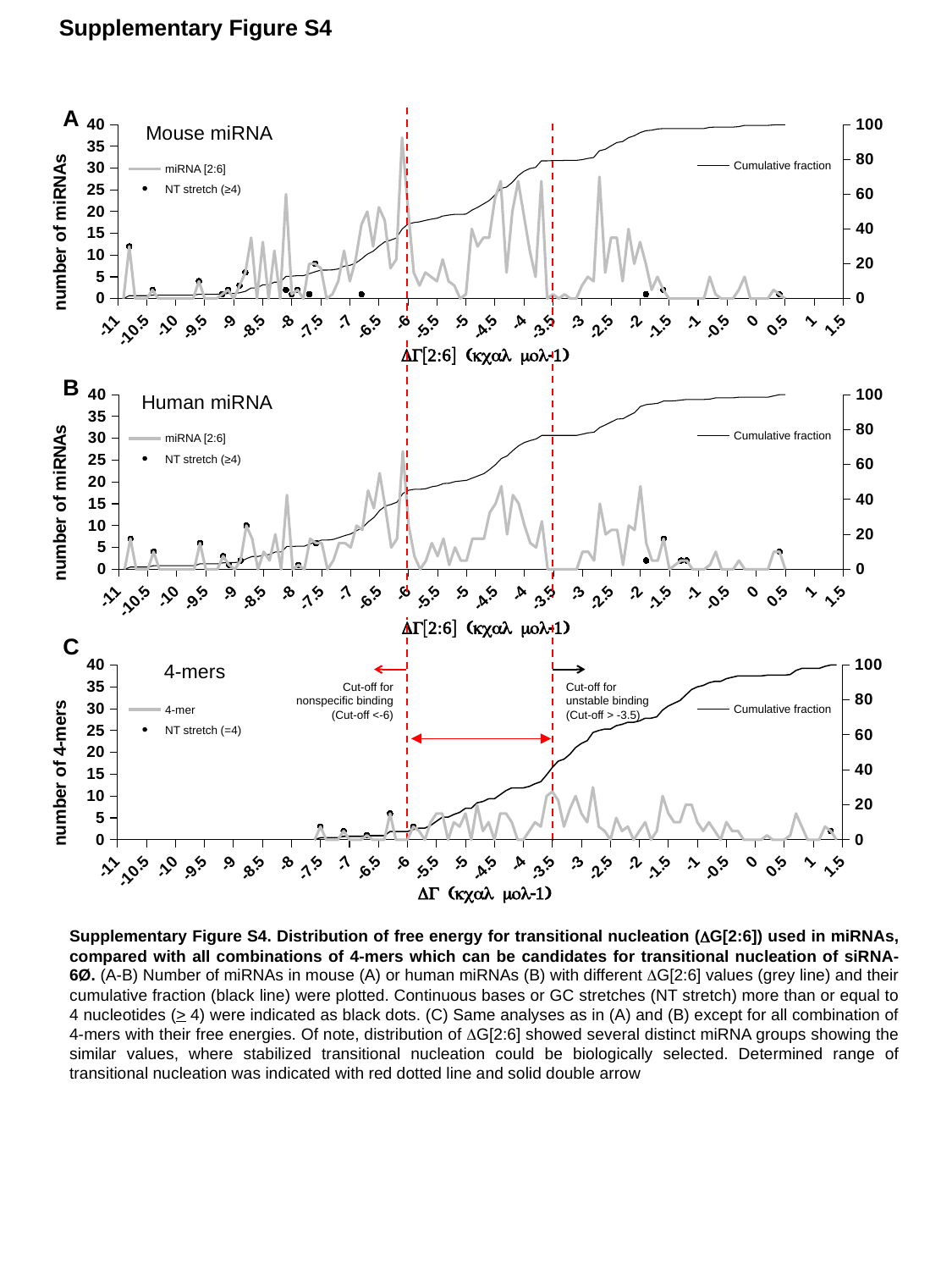

Supplementary Figure S4
A
### Chart
| Category | | | |
|---|---|---|---|Mouse miRNA
Cut-off for
unstable binding
(Cut-off > -3.5)
Cumulative fraction
miRNA [2:6]
NT stretch (≥4)
B
### Chart
| Category | | | |
|---|---|---|---|Human miRNA
Cumulative fraction
miRNA [2:6]
NT stretch (≥4)
C
### Chart
| Category | | | |
|---|---|---|---|4-mers
Cut-off for
nonspecific binding
(Cut-off <-6)
Cumulative fraction
4-mer
NT stretch (=4)
Supplementary Figure S4. Distribution of free energy for transitional nucleation (DG[2:6]) used in miRNAs, compared with all combinations of 4-mers which can be candidates for transitional nucleation of siRNA-6Ø. (A-B) Number of miRNAs in mouse (A) or human miRNAs (B) with different DG[2:6] values (grey line) and their cumulative fraction (black line) were plotted. Continuous bases or GC stretches (NT stretch) more than or equal to 4 nucleotides (> 4) were indicated as black dots. (C) Same analyses as in (A) and (B) except for all combination of 4-mers with their free energies. Of note, distribution of DG[2:6] showed several distinct miRNA groups showing the similar values, where stabilized transitional nucleation could be biologically selected. Determined range of transitional nucleation was indicated with red dotted line and solid double arrow

## Slide 18
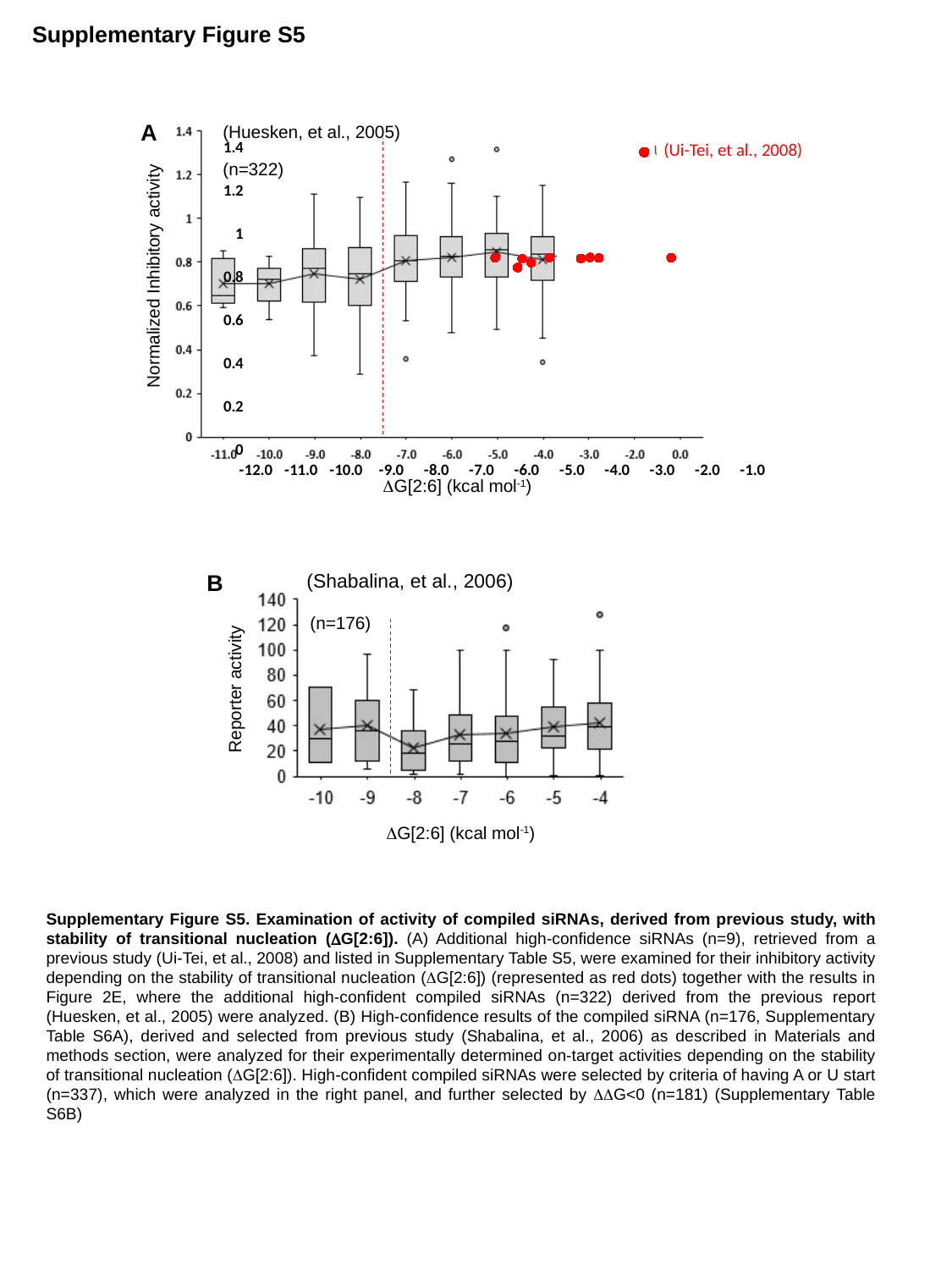

Supplementary Figure S5
A
(Huesken, et al., 2005)
(Ui-Tei, et al., 2008)
### Chart
| Category | |
|---|---|(n=322)
Normalized Inhibitory activity
DG[2:6] (kcal mol-1)
B
(Shabalina, et al., 2006)
(n=176)
Reporter activity
DG[2:6] (kcal mol-1)
Supplementary Figure S5. Examination of activity of compiled siRNAs, derived from previous study, with stability of transitional nucleation (DG[2:6]). (A) Additional high-confidence siRNAs (n=9), retrieved from a previous study (Ui-Tei, et al., 2008) and listed in Supplementary Table S5, were examined for their inhibitory activity depending on the stability of transitional nucleation (DG[2:6]) (represented as red dots) together with the results in Figure 2E, where the additional high-confident compiled siRNAs (n=322) derived from the previous report (Huesken, et al., 2005) were analyzed. (B) High-confidence results of the compiled siRNA (n=176, Supplementary Table S6A), derived and selected from previous study (Shabalina, et al., 2006) as described in Materials and methods section, were analyzed for their experimentally determined on-target activities depending on the stability of transitional nucleation (DG[2:6]). High-confident compiled siRNAs were selected by criteria of having A or U start (n=337), which were analyzed in the right panel, and further selected by DDG<0 (n=181) (Supplementary Table S6B)

## Slide 19
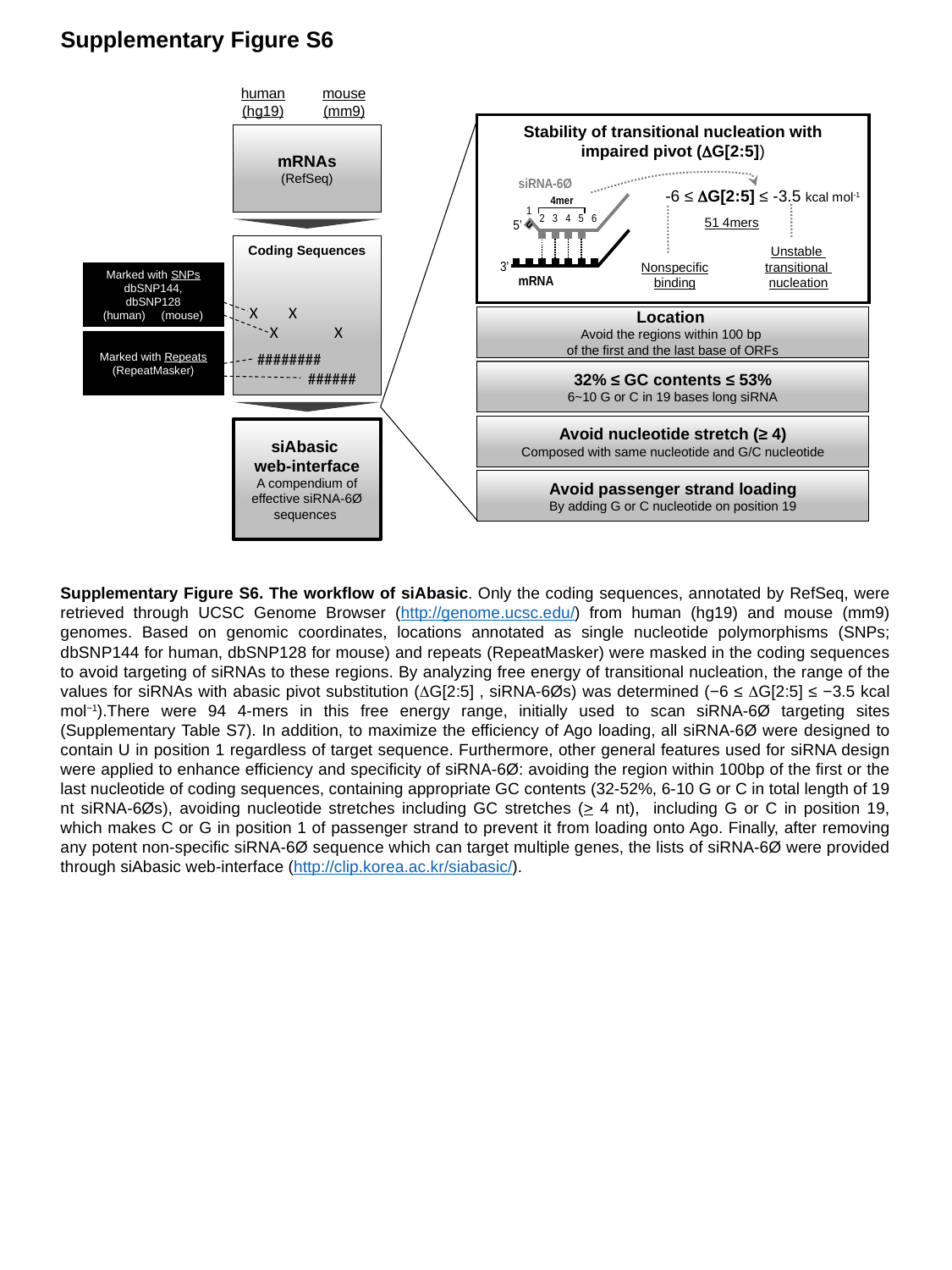

Supplementary Figure S6
mouse
(mm9)
human
(hg19)
Stability of transitional nucleation with impaired pivot (DG[2:5])
mRNAs
(RefSeq)
-6 ≤ DG[2:5] ≤ -3.5 kcal mol-1
51 4mers
Coding Sequences
Unstable
transitional
nucleation
Nonspecific binding
Marked with SNPs
dbSNP144, dbSNP128
(human) (mouse)
X
X
Location
Avoid the regions within 100 bp
of the first and the last base of ORFs
X
X
Marked with Repeats
(RepeatMasker)
########
32% ≤ GC contents ≤ 53%
6~10 G or C in 19 bases long siRNA
######
Avoid nucleotide stretch (≥ 4)
Composed with same nucleotide and G/C nucleotide
siAbasic
web-interface
A compendium of effective siRNA-6Ø sequences
Avoid passenger strand loading
By adding G or C nucleotide on position 19
Supplementary Figure S6. The workflow of siAbasic. Only the coding sequences, annotated by RefSeq, were retrieved through UCSC Genome Browser (http://genome.ucsc.edu/) from human (hg19) and mouse (mm9) genomes. Based on genomic coordinates, locations annotated as single nucleotide polymorphisms (SNPs; dbSNP144 for human, dbSNP128 for mouse) and repeats (RepeatMasker) were masked in the coding sequences to avoid targeting of siRNAs to these regions. By analyzing free energy of transitional nucleation, the range of the values for siRNAs with abasic pivot substitution (DG[2:5] , siRNA-6Øs) was determined (−6 ≤ DG[2:5] ≤ −3.5 kcal mol−1).There were 94 4-mers in this free energy range, initially used to scan siRNA-6Ø targeting sites (Supplementary Table S7). In addition, to maximize the efficiency of Ago loading, all siRNA-6Ø were designed to contain U in position 1 regardless of target sequence. Furthermore, other general features used for siRNA design were applied to enhance efficiency and specificity of siRNA-6Ø: avoiding the region within 100bp of the first or the last nucleotide of coding sequences, containing appropriate GC contents (32-52%, 6-10 G or C in total length of 19 nt siRNA-6Øs), avoiding nucleotide stretches including GC stretches (> 4 nt), including G or C in position 19, which makes C or G in position 1 of passenger strand to prevent it from loading onto Ago. Finally, after removing any potent non-specific siRNA-6Ø sequence which can target multiple genes, the lists of siRNA-6Ø were provided through siAbasic web-interface (http://clip.korea.ac.kr/siabasic/).

## Slide 20
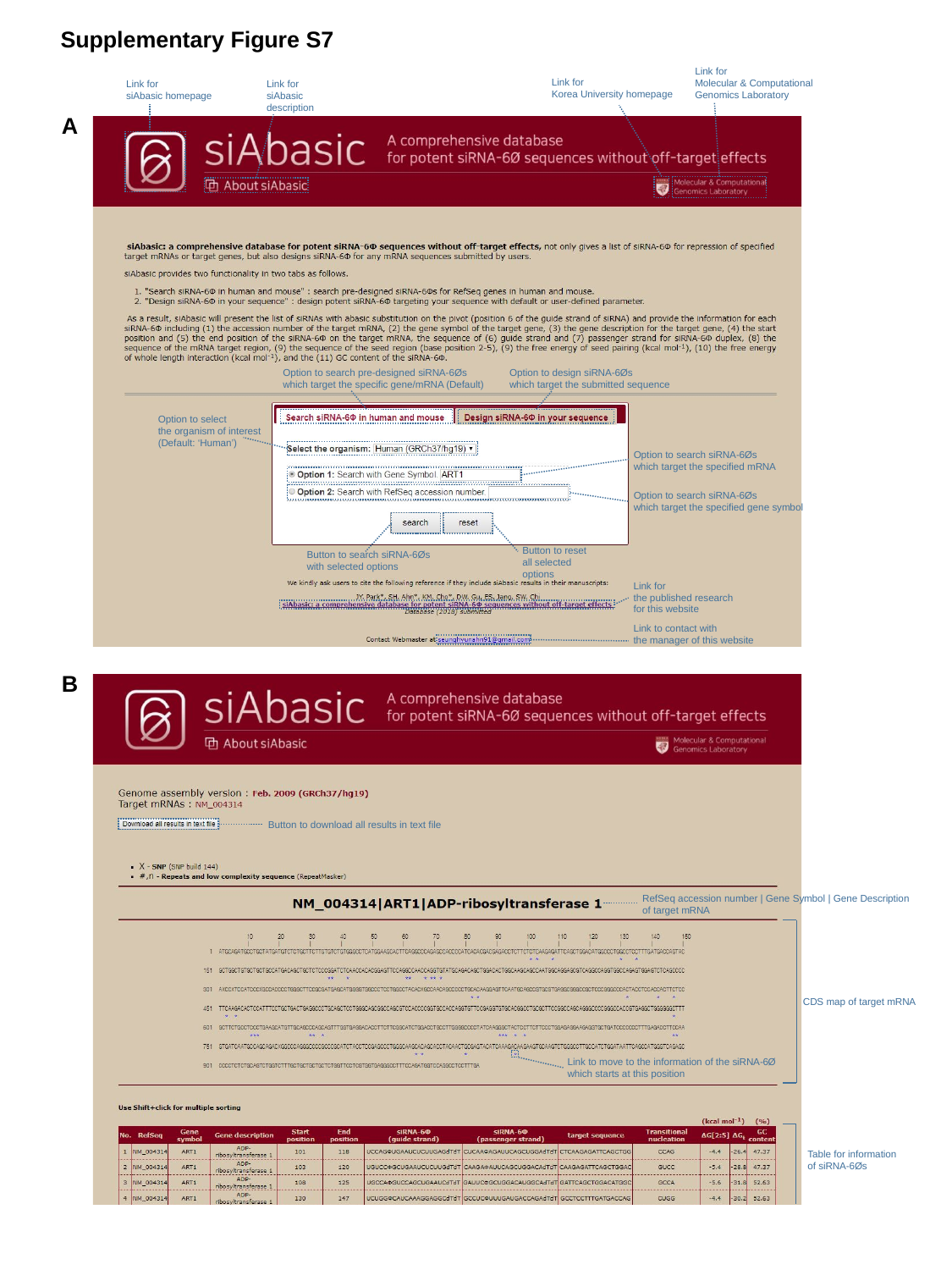

Supplementary Figure S7
Link for
Molecular & Computational
Genomics Laboratory
Link for
Korea University homepage
Link for
siAbasic description
Link for
siAbasic homepage
Option to search pre-designed siRNA-6Øs
which target the specific gene/mRNA (Default)
Option to design siRNA-6Øs
which target the submitted sequence
Option to select
the organism of interest
(Default: ‘Human’)
Option to search siRNA-6Øs
which target the specified mRNA
Option to search siRNA-6Øs
which target the specified gene symbol
Button to reset
all selected options
Button to search siRNA-6Øs
with selected options
Link for
the published research
for this website
Link to contact with
the manager of this website
A
B
Button to download all results in text file
RefSeq accession number | Gene Symbol | Gene Description
of target mRNA
CDS map of target mRNA
Link to move to the information of the siRNA-6Ø
which starts at this position
Table for information
of siRNA-6Øs

## Slide 21
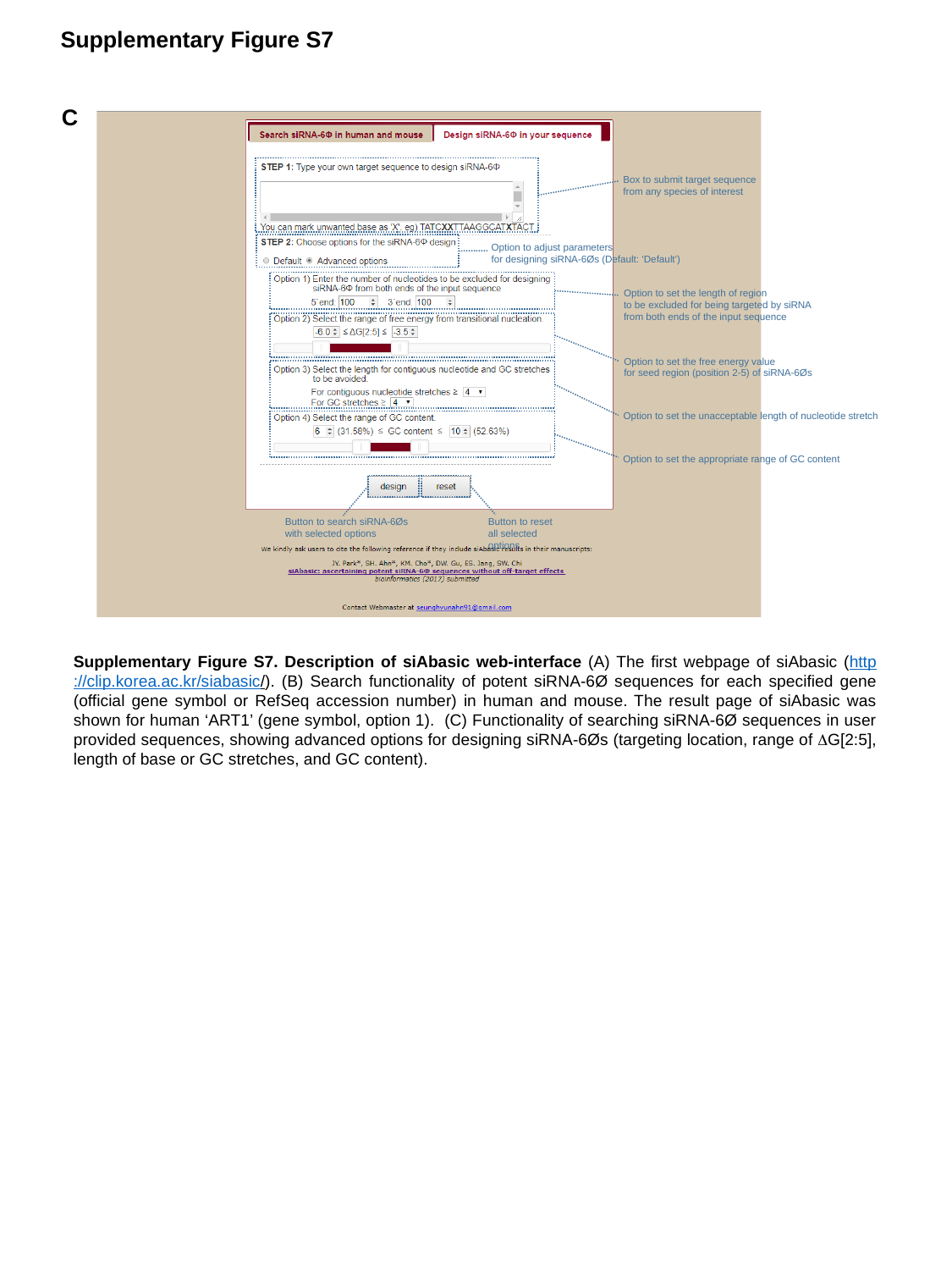

Supplementary Figure S7
C
Box to submit target sequence
from any species of interest
Option to adjust parameters
for designing siRNA-6Øs (Default: ‘Default’)
Option to set the length of region
to be excluded for being targeted by siRNA
from both ends of the input sequence
Option to set the free energy value
for seed region (position 2-5) of siRNA-6Øs
Option to set the unacceptable length of nucleotide stretch
Option to set the appropriate range of GC content
Button to reset
all selected options
Button to search siRNA-6Øs
with selected options
Supplementary Figure S7. Description of siAbasic web-interface (A) The first webpage of siAbasic (http://clip.korea.ac.kr/siabasic/). (B) Search functionality of potent siRNA-6Ø sequences for each specified gene (official gene symbol or RefSeq accession number) in human and mouse. The result page of siAbasic was shown for human ‘ART1’ (gene symbol, option 1). (C) Functionality of searching siRNA-6Ø sequences in user provided sequences, showing advanced options for designing siRNA-6Øs (targeting location, range of DG[2:5], length of base or GC stretches, and GC content).

## Slide 22
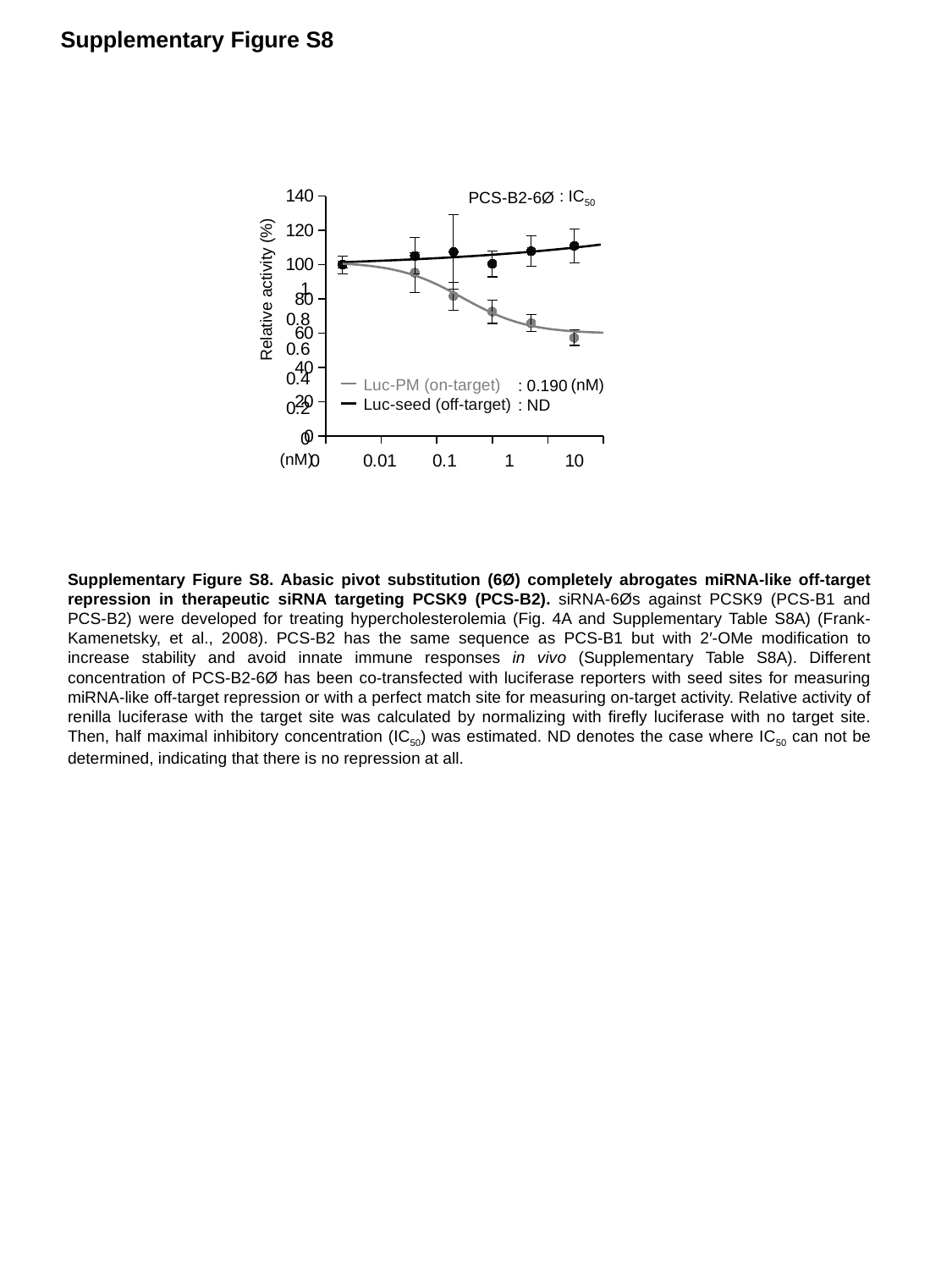

Supplementary Figure S8
### Chart
| Category | | 6pi_on-target | 6pi_off-target | |
|---|---|---|---|---|PCS-B2-6Ø
: IC50
Relative activity (%)
### Chart
| Category | |
|---|---|
| 0 | 0.0 |
| 1.0000000000000004E-2 | 0.0 |
| 0.1 | 0.0 |
| 1 | 0.0 |
| 10 | None |Luc-PM (on-target)
(nM)
: 0.190
Luc-seed (off-target)
: ND
(nM)
Supplementary Figure S8. Abasic pivot substitution (6Ø) completely abrogates miRNA-like off-target repression in therapeutic siRNA targeting PCSK9 (PCS-B2). siRNA-6Øs against PCSK9 (PCS-B1 and PCS-B2) were developed for treating hypercholesterolemia (Fig. 4A and Supplementary Table S8A) (Frank-Kamenetsky, et al., 2008). PCS-B2 has the same sequence as PCS-B1 but with 2′-OMe modification to increase stability and avoid innate immune responses in vivo (Supplementary Table S8A). Different concentration of PCS-B2-6Ø has been co-transfected with luciferase reporters with seed sites for measuring miRNA-like off-target repression or with a perfect match site for measuring on-target activity. Relative activity of renilla luciferase with the target site was calculated by normalizing with firefly luciferase with no target site. Then, half maximal inhibitory concentration (IC50) was estimated. ND denotes the case where IC50 can not be determined, indicating that there is no repression at all.

## Slide 23
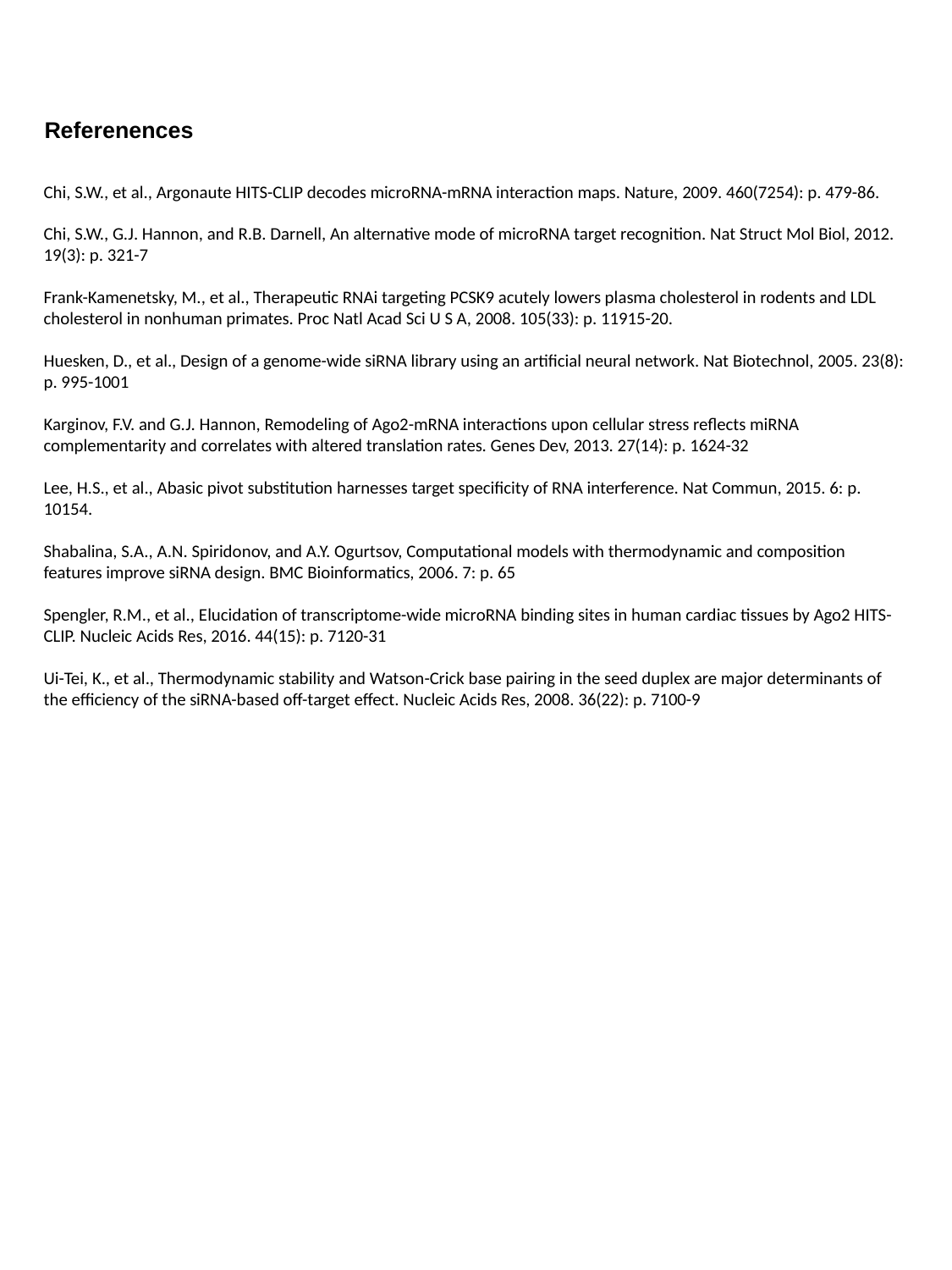

Referenences
Chi, S.W., et al., Argonaute HITS-CLIP decodes microRNA-mRNA interaction maps. Nature, 2009. 460(7254): p. 479-86.
Chi, S.W., G.J. Hannon, and R.B. Darnell, An alternative mode of microRNA target recognition. Nat Struct Mol Biol, 2012. 19(3): p. 321-7
Frank-Kamenetsky, M., et al., Therapeutic RNAi targeting PCSK9 acutely lowers plasma cholesterol in rodents and LDL cholesterol in nonhuman primates. Proc Natl Acad Sci U S A, 2008. 105(33): p. 11915-20.
Huesken, D., et al., Design of a genome-wide siRNA library using an artificial neural network. Nat Biotechnol, 2005. 23(8): p. 995-1001
Karginov, F.V. and G.J. Hannon, Remodeling of Ago2-mRNA interactions upon cellular stress reflects miRNA complementarity and correlates with altered translation rates. Genes Dev, 2013. 27(14): p. 1624-32
Lee, H.S., et al., Abasic pivot substitution harnesses target specificity of RNA interference. Nat Commun, 2015. 6: p. 10154.
Shabalina, S.A., A.N. Spiridonov, and A.Y. Ogurtsov, Computational models with thermodynamic and composition features improve siRNA design. BMC Bioinformatics, 2006. 7: p. 65
Spengler, R.M., et al., Elucidation of transcriptome-wide microRNA binding sites in human cardiac tissues by Ago2 HITS-CLIP. Nucleic Acids Res, 2016. 44(15): p. 7120-31
Ui-Tei, K., et al., Thermodynamic stability and Watson-Crick base pairing in the seed duplex are major determinants of the efficiency of the siRNA-based off-target effect. Nucleic Acids Res, 2008. 36(22): p. 7100-9
